# Insulin resistance is associated with mammary mitochondrial dysfunction at the onset of human lactation

**DOI:** 10.1172/JCI197311

**Published:** 2026-08-13

**Authors:** Xin Meng, Taha Elajnaf, Hussam Rostom, Michelle Ma, Annalee Furst, Bryony R. Davies, Isabella R. Honess, Gaurav Pandey, Isadora C. Furigo, Craig L. Doig, Jayne F. Martin Carli, Rajesh V. Thakker, Lars Bode, Kelsey E. Johnson, Fadil M. Hannan

**Affiliations:** 1 Larsson-Rosenquist Foundation Oxford Centre for the Endocrinology of Human Lactation (LRF-OCEHL), Nuffield Department of Women’s & Reproductive Health, University of Oxford, Oxford, United Kingdom.; 2 Department of Pediatrics, Larsson-Rosenquist Foundation Mother-Milk-Infant Center of Research Excellence (MOMI CORE), and the Human Milk Institute (HMI), UCSD, La Jolla, California, USA.; 3 Center for Discoveries in Life Sciences (DLS), Coventry University, Coventry, United Kingdom.; 4 Centre for Systems Health and integrated Metabolic Research (SHiMR), Department of Biosciences, School of Science and Technology, Nottingham Trent University, Nottingham, United Kingdom.; 5 Department of Obstetrics and Gynecology, Division of Reproductive Sciences, University of Colorado Anschutz Medical Campus, Aurora, Colorado, USA.; 6 Academic Endocrine Unit, Radcliffe Department of Medicine, Oxford Centre for Diabetes, Endocrinology and Metabolism (OCDEM), University of Oxford, Oxford, United Kingdom.; 7 National Institute for Health Research Oxford Biomedical Research Centre, Oxford, United Kingdom.; 8 Centre for Endocrinology, William Harvey Research Institute, Barts and the London School of Medicine, Queen Mary University of London, London, United Kingdom.; 9 Center for Genetic Epidemiology, Keck School of Medicine, University of Southern California, Los Angeles, California, USA.

**Keywords:** Endocrinology, Metabolism, Reproductive biology, Insulin, Mitochondria, Transcriptomics

## Abstract

Insulin resistance (IR) has emerged as a risk factor for lactation insufficiency and delays the onset of milk secretion after childbirth, termed secretory activation (SA). This may cause inadequate infant weight gain and early breastfeeding cessation. However, the mechanisms underlying delayed SA in insulin-resistant women are unknown. To investigate this, we characterized the mammary transcriptomes and IR-related hormones of 75 breastfeeding women with healthy term infants during postpartum days 1–5. Participants were divided into IR tertiles based on plasma leptin-to-adiponectin ratio measurements. Those in the highest tertile had later SA onset with greater neonatal weight loss during postpartum days 1–5. Transcriptomic analysis on postpartum day 2 (*n* = 4 high IR vs. *n* = 8 low IR participants) showed transient suppression of mammary insulin and prolactin signaling genes, increased pro-inflammatory gene expression, and altered expression of more than 200 mammary mitochondrial genes. These alterations were absent on postpartum days 3–5. Cultured mammary epithelial cells treated with insulin showed upregulation of prolactin signaling and oxidative phosphorylation (OXPHOS) genes, with imaging and bioenergetic studies demonstrating that insulin promotes mitochondrial biogenesis and OXPHOS. Thus, our findings delineate roles for insulin in mammary bioenergetics and highlight mitochondrial dysfunction as a mechanism for delayed SA in insulin-resistant women.

## Abstract 2

Insulin resistance causes widespread dysregulation of the mammary transcriptome at the onset of lactation. This includes downregulation of prolactin signaling, mitochondrial biogenesis and oxidative phosphorylation.

## Introduction

Insulin resistance (IR) affects around 40% of women of childbearing age in the United States and has emerged as a lactation insufficiency risk factor (1, 2). Indeed, breastfeeding women with IR-related disorders, such as type 2 diabetes mellitus (T2DM), gestational diabetes mellitus (GDM), obesity, or polyendocrine metabolic ovarian syndrome (PMOS), are reported to experience a delay in the initiation of copious milk secretion after childbirth, which is termed secretory activation (SA) (3–7). Physiological SA occurs within 24–72 hours after childbirth (8), and it is critical for successful lactation, with later onset increasing the risk of low milk supply and inadequate infant weight gain (9, 10). During SA, human alveolar mammary epithelial cells (MECs) transition from producing 30–40 mL of colostrum on postpartum day 1 to ~600 mL milk daily by postpartum day 4 (11, 12). This transition is mediated by an increase in mammary lactose synthesis and the closure of MEC paracellular tight junctions, which establish an osmotic gradient that drives water into milk (13). The marked upregulation of mammary synthetic capacity during SA is also characterized by increased MEC gene transcription and translation along with enlargement of cell organelles such as the nucleus, ER, and Golgi (14).

Human SA is triggered by physiological hyperprolactinemia combined with the abrupt decline of circulating progesterone by 30–40 hours following placental delivery (8, 13). However, a comprehensive understanding of the endocrine basis of human SA is lacking, and clinical studies combined with transcriptomic analyses have highlighted the potential importance of insulin. Indeed, transcriptomic characterization of milk fat globules (MFGs), which provide an abundant source of lactating MEC RNA, have shown early postpartum increases in insulin signaling gene expression (15). Thus, MEC genes encoding the insulin receptor (*INSR*) and downstream insulin receptor substrate 2 (*IRS2*), as well as the PI3K regulatory subunit 2 and Akt serine/threonine kinase 1 (*AKT1*) signaling proteins, which mediate the metabolic actions of insulin, all showed >2-fold expression increases during the transition from colostrum to milk production (15). Despite these advances, the contribution of insulin to MEC function and SA biology in humans remains unclear. Indeed, glucose uptake by the lactating mammary gland may occur in an insulin-dependent manner involving the glucose transporter type 1 (16, 17). We hypothesized that insulin upregulates mammary metabolic processes soon after childbirth and that IR leads to MEC metabolic dysfunction by impairing the expression of genes involved in mammary gland energy and substrate generation. To address this, we evaluated the impact of IR on SA and the early-postpartum MFG transcriptome in healthy breastfeeding women and in parallel characterized the effect of insulin on MEC signal transduction, gene expression dynamics, and bioenergetic processes supporting milk synthesis.

## Results

### Assessment of IR using the plasma leptin-to-adiponectin ratio.

Seventy-five breastfeeding women with healthy term pregnancies were investigated at 36 weeks’ gestation and on 1 or more days during postpartum days 1–5 (18) (Figure 1A). Participants were categorized based on their nonfasting plasma leptin-to-adiponectin ratio (LAR) measured at 36 weeks’ gestation into tertiles of *n* = 25 designated as low (LAR ≤ 2 ng/μg), medium (LAR > 2 to ≤ 5 ng/μg), or high (LAR > 5 ng/μg) degree of IR (Supplemental Figure 1 and Supplemental Table 1; supplemental material available online with this article; https://doi.org/10.1172/JCI197311DS1). LAR is reported as an IR measure in nondiabetic individuals (19), and it was validated in an additional 20 breastfeeding women who provided postpartum fasting blood samples for homeostasis model assessment (HOMA) (Supplemental Figure 1) (20, 21). This showed significant correlations between LAR and HOMA measures (Supplemental Figure 1). Plasma LAR also correlated with BMI, assessed at 8–10 weeks’ gestation, and with glucose tolerance test values measured at 24–36 weeks’ gestation during the routine care of participants with GDM risk factors (Supplemental Figure 1). Plasma LAR was not associated with parity, comorbidities, pregnancy duration, delivery mode, infant birthweight, or the type of infant feeding (Table 1). However, participants were slightly younger in the high IR group (33.2 ± 0.9 years vs. 36.0 ± 0.8 years in the low IR group, *P* < 0.05, 1-way ANOVA) (Table 1).

### IR is associated with later onset of SA and neonatal weight loss.

Daily assessments of postpartum increases in breast fullness, which indicates SA timing (10), showed that high IR participants had later onset of milk coming in (2.8 ± 0.1 days vs. 2.5 ± 0.1 days postpartum for the low IR group, *P* < 0.05, χ^2^ test) (Figure 1B). This was associated with increased neonatal weight loss during postpartum days 1–5 (–6.1% for high IR group vs. –2.7% for low IR group, *P* < 0.001, 1-way ANOVA) (Table 1).

The impact of IR on SA was further assessed using milk lactose and electrolyte measurements, as lactation onset is characterized by a rise in lactose together with decreasing Na^+^ and increasing K^+^ concentrations ≤72 hours postpartum (12). These changes in milk lactose and electrolytes, which are mediated by increasing MEC synthetic function and tight junction closure, respectively, are early indicators of SA and precede the increase in milk volume and breast fullness occurring in this period (12). The accuracy of milk lactose, Na^+^, and K^+^ measurements was assessed by spike and recovery and demonstrated 80%–111% recovery (Supplemental Table 2). High IR participants had lower milk lactose compared with the low IR group on postpartum day 1 (77 ± 18 mmol/L vs. 113 ± 17 mmol/L, *P* < 0.05, 2-way ANOVA) (Figure 1C). Moreover, milk Na^+^ was significantly elevated in the high IR group on postpartum day 1 (77 ± 14 mmol/L vs. 51 ± 7.8 mmol/L for the low IR group, *P* < 0.05, 2-way ANOVA), though no differences were observed in milk K^+^ between high and low IR groups (Figure 1, D and E). The milk Na^+^/K^+^ ratio, which is used as a SA biomarker (15, 22), was also significantly elevated in the high IR group on postpartum day 1 (5.3 ± 1.0 mmol/mmol vs. 3.3 ± 0.8 mmol/mmol for the low IR group, *P* < 0.05, 2-way ANOVA) but normalized thereafter (Figure 1F).

A longitudinal assessment of IR-related hormones showed that plasma leptin decreased in all participants postpartum, as reported (Figure 1G) (23). In contrast, plasma adiponectin showed no temporal change but was significantly lower in high IR participants at 36 weeks’ gestation and on postpartum day 1, whereas postpartum plasma LAR decreased in all groups, mirroring the leptin decrease (Figure 1, H and I). Serum nonfasted insulin was elevated in high IR participants at 36 weeks’ gestation but showed no significant postpartum change (Supplemental Figure 2). Furthermore, the later SA onset in high IR participants was not associated with alterations in serum progesterone or prolactin, which mediate lactation onset after childbirth (Supplemental Figure 2) (8, 13).

### IR is associated with mammary transcriptome dysregulation during SA.

To explore the underlying mechanisms of altered SA in insulin-resistant breastfeeding participants, we performed bulk RNA-seq on the MFG fraction from postpartum day 2–5 colostrum and milk samples donated by 71 participants (total of 106 samples) (Figure 2A and Supplemental Table 1) (15). MFG RNA quality was variable with RNA integrity number (RIN) values ranging 1.3–9.1 (Supplemental Figure 3). However, principal components analysis showed no clustering of MFG RNA-seq data according to RIN value (Supplemental Figure 3). Moreover, to account for any bias due to variable RNA quality, RIN values were used as covariates in all gene expression analyses. Samples from 19 to 37 participants (25%–49% of the cohort) were analyzed per postpartum day (Figure 2B and Supplemental Table 1).

MFG RNA-seq has been validated for assessing the lactating MEC transcriptome in different species (Supplemental Table 3), and a comparison with tissue expression profiles from the Genotype-Tissue Expression and Mouse Gene Atlas databases showed that the MFG transcriptomes exhibited the greatest similarity to human female mammary tissue and mouse lactating mammary gland (Supplemental Figure 4) (24, 25). IR did not affect total numbers of MFG transcripts, of which genes encoding milk proteins (*LALBA*, *CSN2*, *CSN1S1*, and *LTF*) were most abundant and comprised 30% of all transcripts (Supplemental Figure 4) (26). However, on postpartum day 2, 4,425 out of a total of 17,669 different transcripts (25%) were differentially expressed with a log_2_ fold-change ≥ 0.5 (meta-*q* < 0.05) when high IR (*n* = 4 participants) and low IR (*n* = 8 participants) groups were compared (Figure 2, B and C; Supplemental Figure 5; and Supplemental Table 4). Characteristics, such as maternal age, parity, BMI, delivery mode, timing of milk collection, or provision of breastfeeding support, did not differ between high and low IR groups on postpartum day 2 (Supplemental Table 5). However, high IR participants, whose MFG transcriptomes were assessed on postpartum day 2, reported later SA than low IR participants, and their infants had greater postpartum day 1–5 weight loss (Supplemental Table 5). A comparison of the medium (*n* = 7 participants) and low IR (*n* = 8 participants) groups on postpartum day 2 led to only 14 DEGs being detected, of which 13 overlapped with those identified in high IR participants (Figure 2B, Supplemental Figure 5, and Supplemental Table 6). No DEGs were detected on days 3–5 postpartum when comparing high, medium, and low IR groups, even when using a less stringent meta-*q* threshold of 0.1 (Figure 2B and Supplemental Tables 7–9). Thus, IR may affect lactation only during the colostrum stage (Figure 2, B and C). We assessed whether differences in BMI contributed to the differential gene expression observed between high and low IR groups. None of the 19 participants, whose MFG transcriptomes were assessed on postpartum day 2, were obese (Supplemental Table 5), but a comparison of participants with BMI > 25 kg/m^2^ (range 25.3–28.6 kg/m^2^, *n* = 6) and those with BMI < 25 kg/m^2^ (range 19.0–24.6 kg/m^2^, *n* = 13) yielded only 43 DEGs on postpartum day 2, of which 40 were also identified in the high IR group (Supplemental Figure 5 and Supplemental Table 10).

Genes with the greatest differential expression on postpartum day 2 showed significant correlation between their expression and third-trimester plasma LAR values (Supplemental Figure 6). The top upregulated genes included inflammatory components, such as *CXCL8* (16-fold increase, meta-*q* = 3.3 × 10^–5^), while genes encoding mitochondrial proteins, such as *ARG2* (4.7-fold decrease, meta-*q* = 4.7 × 10^–12^), were among the most downregulated (Figure 2C and Supplemental Figure 6). However, expression of these genes on postpartum days 3–5 generally showed no correlation with third-trimester plasma LAR values (Supplemental Table 11).

Gene set enrichment analysis (GSEA) demonstrated that pro-inflammatory processes were most highly upregulated in the high IR group (Figure 2D). Upregulated inflammatory genes were involved in biological processes ranging from TGF-β signaling to apoptosis (Figure 2E). This pro-inflammatory gene signature may be due to a high abundance of immune cells within the colostrum MFG fraction (15). We assessed this by flow cytometry and demonstrated that <5% of cells within the MFG fraction were of immune origin (CD45^+^), while >95% were nonimmune cells (CD45^–^), most likely from the mammary epithelium (Supplemental Figure 7). There was no association between LAR and the number of cells within the MFG fraction (Supplemental Figure 7).

Consistent with IR being associated with systemic inflammation (27), participants in the high IR group whose MFG transcriptomes were assessed on postpartum day 2 had higher third-trimester serum C-reactive protein (CRP) concentrations (7.7 ± 1.8 mg/L) than the low IR group (1.9 ± 0.4 mg/L, *P* < 0.05, Kruskal-Wallis test) (Figure 2F). All participants, irrespective of IR group, had elevated CRP on postpartum day 2, likely due to parturition-associated acute inflammation (Figure 2G) (28). To determine whether systemic inflammation may have contributed to the altered mammary gene expression on postpartum day 2, we grouped participants into tertiles according to their third-trimester CRP values (Supplemental Figure 8 and Supplemental Table 12). A comparison of postpartum day 2 MFG transcriptomes from participants in the low (CRP, 0.47–1.2 mg/L, *n* = 5) and high (CRP, 4.5–11.1 mg/L, *n* = 5) tertiles revealed 67 DEGs (Supplemental Figure 8 and Supplemental Table 13). This represented <2% of the 4,425 DEGs observed in high IR participants, indicating that inflammation alone is unlikely to account for the widespread early-postpartum altered mammary gene expression.

Protein synthesis and branched chain amino acid catabolism were the most downregulated processes in high IR participants (Figure 2D). Expression of the signaling gene, *JAK2*, which can be activated in insulin-resistant states (29), was increased in high IR participants (Figure 2H). IR was associated with lower expression of mammary insulin signaling genes (*INSR*, *IRS1*, *AKT1*), which promote metabolic processes supporting lactation, such as glucose uptake and lipid synthesis (30), and genes encoding the mTOR complex 1, which probably increase lactating MEC protein translation, also showed lower expression (Figure 2H) (31). Moreover, high IR participants had lower expression of genes coordinating milk synthesis, such as *STAT5A*, a prolactin signaling component, and mammary alveologenesis transcription factors (*EHF*, *ELF5*, *GATA3*), (Figure 2H and Supplemental Figure 6) (13, 32–34). Genes involved in de novo fatty acid synthesis (*SREBF1*, *FASN*) and the biogenesis and secretion of MFGs into milk (*PLIN3*, *CIDEA*, *XDH*, *MUC1*) also showed lower expression (Figure 2H and Supplemental Figure 6) (35–38). Furthermore, IR affected mammary protein synthesis with lower expression of ribosomal and casein genes (*CSN2*, *CSN3*) (Figure 2H). The *B4GALT1* lactose synthase subunit was also downregulated and probably contributed to the low milk lactose concentrations in high IR participants (Figure 1C and Figure 2H). In addition, expression of genes involved in parathyroid hormone–related peptide-mediated Ca^2+^ mobilization from the maternal skeleton (*PTHLH*), influx of circulating Ca^2+^ via store-operated Ca^2+^ channels into MECs (*ORAI1*), and Ca^2+^ efflux from MECs into milk (*NHERF1*) were decreased in the high IR group (Figure 2H) (39–42). Finally, genes encoding tight junction proteins (*TJP2*, *TJP3*, *CLDN8*) were downregulated (Figure 2H), which may explain the increased milk Na^+^ and milk Na^+^/K^+^ ratio in high IR participants (Figure 1, D and F).

IR was also associated with 266 nuclear encoded mitochondrial DEGs (Figure 2, H and I). To ascertain which mitochondrial processes are altered in high IR participants, DEGs were categorized according to MitoCarta3.0 pathway annotations (Supplemental Table 14) (43). This revealed downregulation of genes involved in oxidative phosphorylation (OXPHOS), mitochondrial nutrient metabolism, mitochondrial protein import, and mitochondrial genome transcription and translation (Figure 2I). In contrast, genes encoding mitochondrial proteins promoting cellular apoptosis were upregulated (Figure 2I). These findings were suggestive of IR-mediated mammary mitochondrial dysfunction. To determine if this was a consequence of defective insulin action, we proceeded to assess the effect of insulin in cultured MECs.

### Influence of insulin and the Akt signaling protein on the cultured human MEC transcriptome.

The human MCF-12A mammary cell line, which expresses β-casein in the presence of prolactin, insulin, and glucocorticoids, was used for these studies (44). RT-qPCR analysis demonstrated that MCF-12A cells express β-casein (*CSN2*), *INSR*, and *AKT1*, which encodes the Akt signaling protein (Supplemental Figure 9) (30, 45).

Cells were treated with 0, 10, or 100 nM insulin followed by bulk RNA-seq at 0, 1, 4, and 8 hours (Figure 3A). Insulin altered the MCF-12A cell transcriptome in a dose- and time-dependent manner (Figure 3B and Supplemental Figure 10). The effects of insulin peaked at 4 hours posttreatment where out of 17,708 transcripts, 4,571 (26%) and 5,207 (29%) were significantly altered with a log_2_ fold-change ≥ 0.5 (meta-*q* < 0.05) by 10 nM and 100 nM insulin, respectively (Figure 3B, Supplemental Figure 10, and Supplemental Tables 15 and 16). Insulin upregulated genes involved in lactation, including prolactin signaling (*SOCS2*) (46), secretory differentiation and SA (*HIF1A*) (47), tight junction formation and closure (*TJP2*, *TJP3*, *CLDN8*) (48), lipid droplet biogenesis (*XDH*, *CIDEA*, *PLIN3*) (38, 49), and calcium mobilization and transport (*PTHLH*, *ORAI1*) (Supplemental Tables 15 and 16) (40). The 10 and 100 nM insulin doses had concordant effects on gene expression (*r* = 0.97; *P* < 0.0001), with 4,029 out of 5,745 transcripts (70%) induced by both insulin doses being similarly altered (Figure 3C and Supplemental Figure 11).

Dynamic changes in the MCF-12A cell transcriptional response to 100 nM insulin were also assessed using time-series clustering of gene expression (TMixClust) (50). Silhouette analysis identified 2 major temporal patterns characterized by peak expression changes occurring with either 4 hours (cluster 1: transient increase, *n* = 70 genes; and cluster 2: transient decrease, *n* = 505 genes) or 8 hours of insulin treatment (cluster 3: gradual decrease, *n* = 69 genes; and cluster 4: gradual increase, *n* = 442 genes) (Figure 3D, Supplemental Figure 10, and Supplemental Table 17). Clusters 1 and 2 included lactation genes, such as *SOCS2*, *PTHLH*, and *GATA3*, whereas clusters 3 and 4 contained insulin signaling genes (*IRS2*, *FOS*) and mitochondrial genes (*MSTO1*, *MPV17L*) (Figure 3E and Supplemental Table 17). Gene Ontology analysis showed enrichment of genes with a transient change in expression (clusters 1 and 2) in ribosome biogenesis, cell-cell adhesion, and metal ion transport, whereas genes with a gradual change in expression (clusters 3 and 4) were enriched in the MAPK pathway, metabolic processes, and cell cycle (Figure 3F).

Next, we investigated the role of Akt in insulin-induced transcription by knocking down this signaling protein prior to 0 and 100 nM insulin treatment (Figure 4A). Administration of Akt-targeted siRNA decreased *AKT1* RNA expression by 83% compared with use of nontargeted (scramble) siRNA (Supplemental Figure 9). Knockdown also decreased Akt protein by 60% and attenuated phosphorylated Akt (p-Akt) following 100 nM insulin for 30 minutes (Figure 4, B and C). Furthermore, Akt knockdown mitigated the transcriptional effects of insulin. In cells treated with 100 nM insulin for 4 hours, Akt knockdown decreased the number of DEGs from 5,204 to 2,779 (Figure 4D). We modeled the interaction between insulin and Akt on gene expression at 4 hours using DESeq2 (51). This identified 2,648 genes (51% of insulin-induced genes, log_2_ fold-change > 0.5 at adjusted *P* < 0.05) that were significantly altered by insulin in an Akt-dependent manner (Figure 4E and Supplemental Table 18). Out of the 2,648 genes, Akt knockdown attenuated the expression of 2,603 genes (98% of insulin-Akt dependent genes), while 45 genes showed enhanced expression (Figure 4E and Supplemental Table 18). The attenuated genes were involved in hormone signaling (*NR3C1*, *SOCS2*), secretory differentiation and SA (*HIF1A*), and lipid droplet biogenesis (*XDH*, *PLIN2*) (Supplemental Table 18). Biological pathway analysis showed an enrichment of insulin-induced genes involved in ribosome biogenesis and RNA processing, ER stress response, and ER-to-cytosol transport (Figure 4F). Mitochondrial biogenesis processes, such as structural organization and transfer RNA processing, were also enriched after insulin (Figure 4F). However, both protein synthesis and mitochondrial processes were no longer enriched after Akt knockdown (Figure 4F).

### Influence of insulin on mitochondrial gene expression in cultured mammary cells.

In MCF-12A cells, 100 nM insulin altered the expression of 259 genes encoding mitochondrial proteins (Figure 5A and Supplemental Table 19). These genes are involved in OXPHOS, mtDNA maintenance, import of nuclear encoded proteins, and mitochondrial lipid metabolism (Figure 5B). Akt knockdown affected insulin-mediated changes in the expression of 158 out of 259 mitochondrial genes (61%) (Figure 5C).

Treatment of MCF-12A cells with 100 nM insulin for 8 hours increased the expression of 10 genes involved in mitochondrial ATP generation, thus highlighting a potential role for this hormone in promoting mammary OXPHOS (Figure 5D). We validated this using the murine HC11 cell line, an established model for studying mammary cell mitochondria (52, 53). HC11 cells were differentiated to express β-casein (54), then treated with 100 nM insulin for 8 hours prior to bulk RNA-seq (Supplemental Figure 12). Insulin altered the expression of 3,128 (27%) out of 11,791 transcripts, of which 1,589 were upregulated and 1,539 were downregulated (Figure 5E, Supplemental Figure 13, and Supplemental Table 20). Biological pathway analysis showed that the major effects of insulin in HC11 cells were on mitochondrial processes (Figure 5, F and G). This included increased expression of genes involved in OXPHOS, mitochondrial import and folding of nuclear-encoded proteins, transcription and translation of the mitochondrial genome, and structural organization mediated by the mitochondrial contact site and cristae organizing system (Supplemental Figure 14 and Supplemental Table 21) (55).

### Insulin promotes mitochondrial biogenesis and OXPHOS in cultured mammary cells.

Insulin had a pronounced effect on mitochondrial gene expression in murine HC11 cells. These cells were used to investigate whether insulin may promote mitochondrial biogenesis as assessed by changes in mitochondrial volume and morphology. Cells were treated with 0, 10, and 100 nM insulin for 24 hours and labeled with a mitochondrial probe (PK Mito Orange) (Figure 6A). This revealed that 10 and 100 nM insulin increased mitochondrial volume in a concentration-dependent manner (2.54 ± 0.16 μm^3^ [*P* < 0.05] and 2.66 ± 0.15 μm^3^ [*P* < 0.01], respectively, vs. 2.04 ± 0.07 μm^3^ for control cells, 1-way ANOVA), consistent with increased mitochondrial biogenesis (Figure 6, B and C). However, insulin did not affect mitochondrial length or the number of branches, which are indicators of fusion and fission events (Figure 6, D and E) (56). Next, we investigated the effect of hyperinsulinemia, which occurs in IR conditions, such as obesity and T2DM, by treating cells with 1,000 nM insulin, as reported (57, 58). Exposure to 1,000 nM insulin for 24 hours led to decreases in mitochondrial volume and length and number of branches, as well as increasing mitochondrial roundness and fragmentation (Figure 6, B–F). Transmission electron microscopy was used to confirm these findings. This showed that cells treated with 1,000 nM insulin for 24 hours had predominantly small, rounded mitochondria, whereas cells treated with 0, 10, or 100 nM insulin had both rounded and tubular mitochondria (Supplemental Figure 15).

We determined if insulin-mediated mitochondrial biogenesis alters bioenergetics using live cell monitoring of O_2_ and pH as indicators of OXPHOS and glycolysis, respectively (59). HC11 cells treated with 10 and 100 nM insulin showed a concentration-dependent decrease in basal intracellular O_2_ and increase in O_2_ consumption rate (OCR), consistent with increased OXPHOS (Figure 7, A and B). Insulin (100 nM) had a more pronounced effect on maximal O_2_ consumption induced by carbonylcyanide *p*-trifluoromethoxyphenylhydrazone (FCCP), a mitochondria uncoupler, and resulted in ~3-fold increase in maximal OCR compared with untreated cells (*P* < 0.01, 1-way ANOVA) (Figure 7, C and D). Furthermore, insulin caused a concentration-dependent decrease in pH and increase in the extracellular acidification rate (ECAR), consistent with increased basal glycolysis (Figure 7, E and F). Glycolytic capacity, which was calculated as the difference between oligomycin-stimulated glycolysis and glycolysis-independent extracellular acidification (60), significantly increased following 100 nM insulin (*P* < 0.001, 1-way ANOVA) (Figure 7, G and H). These findings indicate that insulin enhances both basal and maximal glycolysis and OXPHOS in HC11 cells.

### Transcriptional components mediating effects of insulin on mitochondrial gene expression.

In skeletal muscle, insulin is reported to influence mitochondrial biogenesis and cellular bioenergetics by regulating peroxisome proliferator–activated receptor γ coactivator-1-alpha (PGC-1α) expression (61, 62). PGC-1α is a transcriptional coactivator that acts via nuclear transcription factors (TFs), including nuclear respiratory factor-1 (NRF-1), to increase expression of genes mediating mitochondrial formation and function (63). However, in HC11 cells, insulin paradoxically decreased PGC-1α gene expression (Supplemental Figure 16A), despite this hormone increasing both mitochondrial gene expression and volume (Figures 5 and 6). Moreover, treatment with 10 and 100 nM insulin for 8 hours had no effect on PGC-1α protein abundance (Supplemental Figure 16, B and C). Thus, insulin may be acting by a distinct transcriptional mechanism in the mammary gland compared with skeletal muscle, which we explored using chromatin immunoprecipitation sequencing (ChIP-seq) data from the ChIP-Atlas (64). In silico analysis of TFs binding to highly upregulated mitochondrial genes in insulin-treated HC11 cells was performed using murine lactating mammary gland and skeletal muscle ChIP-seq data (Supplemental Table 22) (64). This revealed 14 TFs to bind the promoter region of these mitochondrial genes, with 9 TFs associated with mitochondrial gene loci in the lactating mammary gland but not skeletal muscle (Supplemental Figure 16D). These mammary-selective TFs include established mediators of lactation, such as STAT5A and the E74 like ETS transcription factor 5, as well as nuclear receptors binding progesterone and estradiol (Supplemental Figure 16D). In contrast, the NRF-1 TF was shown to associate with mitochondrial gene loci in skeletal muscle but not the lactating mammary gland (Supplemental Figure 16D).

## Discussion

Reported MFG transcriptomic studies have revealed mammary processes, such as prolactin signaling, and the synthesis of lactose, fatty acids, and milk proteins, to be enhanced during the shift from colostrum to transitional milk production (15, 17). Our findings indicate that insulin may contribute to such processes, as they were downregulated in participants with a higher degree of IR (Figure 2H). IR was also associated with increased mammary *JAK2* gene expression (Figure 2H). The JAK2 tyrosine kinase has been reported in IR states to impair activation of Akt, which is required for SA (29, 30). We also identified downregulated mammary mitochondrial processes in high IR participants, whereas in cultured MECs, insulin increased mitochondrial volume, gene expression, and bioenergetics, consistent with enhanced mitochondrial biogenesis (Figures 5–7). These findings support insulin regulating mammary mitochondrial function, which may promote energy generation and provide substrates for milk synthesis during SA (65).

Insulin sensitivity is commonly evaluated by HOMA (20, 21). However, obtaining fasting blood during postpartum days 1–5 for HOMA was possible in only a few participants due to the challenge of fasting while initiating breastfeeding soon after childbirth. Instead, we utilized the nonfasting LAR as an IR surrogate during late pregnancy and the early postpartum period, as LAR and HOMA similarly correlate with the insulin sensitivity index obtained from hyperinsulinemic-euglycemic clamp studies (19). Moreover, LAR is reported to correlate with HOMA during pregnancy (66), and we demonstrated a significant association between these measures during the first postpartum month (Supplemental Figure 1). As our study is the first to utilize this ratio early postpartum, further validation of LAR for grading IR in breastfeeding women is warranted.

In this study, IR was associated with a mild SA delay, with nearly all participants initiating lactation ≤72 hours postpartum (Figure 1B). Consistent with this, IR altered the MFG transcriptome on postpartum day 2 but not at later time points. This suggests that insulin may have the greatest mammary gland effects during parturition or pregnancy. Indeed, the differentiation of MECs into milk-secreting alveoli during pregnancy is a prerequisite for successful SA and reported to be regulated by insulin in mice (13, 67). Administration of high-fat diets to induce IR during pregnancy or peripartum in genetically susceptible mouse strains may be helpful to determine when IR has the greatest effect on SA. Such models could also be used to evaluate the effect of IR on MEC insulin signaling and correlations with SA timing (68).

We showed that approximately 50% of insulin-mediated transcriptional changes were attenuated upon Akt knockdown, indicating that this signaling protein is likely required for the full insulin response in MECs (Figure 4). Consistent with Akt being critical for SA (30), insulin-stimulated genes regulating prolactin signaling, secretory differentiation, and lipid droplet biogenesis showed attenuated expression upon Akt knockdown. We also found that Akt knockdown altered the expression of 158 mitochondrial genes in MECs, in keeping with Akt regulating mitochondrial function in nonmammary tissues (69).

Our study indicates that mitochondrial genes are a major insulin target in MECs. We showed that IR is associated with reduced mammary mitochondrial gene expression, consistent with IR-related conditions such as T2DM and obesity also downregulating OXPHOS genes in skeletal muscle (70). The suppression of OXPHOS genes may impair SA, as this is characterized by increased metabolic demand (71). It is notable that insulin influenced OXPHOS gene expression to a greater extent in murine HC11 cells than human MCF-12A cells (Figure 5). This may be due to species differences or a consequence of HC11 cells being derived from the pregnant mammary gland, which undergoes differentiation into a lactation-competent organ, and therefore is likely to have increased metabolic activity (72).

Insulin increased mitochondrial volume with no significant effect on morphology in cultured MECs (Figure 6). This contrasts with cardiomyocytes, where insulin altered both mitochondrial morphology and volume by promoting mitochondrial fusion and cristae remodeling (73). Our cultured MEC studies also showed that exposure to a high insulin concentration (1,000 nM) caused mitochondrial fragmentation, similar to that reported in the skeletal muscle of IR individuals (70).

Limitations include the observational nature of our clinical study, which precludes a causal link between IR and altered SA being established. We also characterized healthy breastfeeding women with very few IR-associated comorbidities (Table 1). Thus, IR is likely to be mild and may explain why only transient alterations of the MFG transcriptome were observed (Figure 2B). However, even a mild degree of IR may have clinical implications. Indeed, this was associated with a slight physiological delay in SA in our cohort but more pronounced early postpartum neonatal weight loss (Table 1). Further investigation of breastfeeding women with T2DM, GDM, PMOS, or obesity will help determine if more severe IR may be linked to prolonged derangements of MEC biology that result in lactation insufficiency and require interventions to improve milk supply. Our cohort also consisted mainly of women aged ≥30 years, who all had healthy term infants. Further work is required to understand the impact of IR on younger women and those with preterm or small-for-gestational-age infants. In addition, most participants were expressing small quantities of milk during the first few days after childbirth. This limited the longitudinal nature of this study with <50% of participants providing samples of sufficient volume for biochemical and RNA-seq analyses on each postpartum day. We were also unable to obtain sufficient colostrum volumes within 24 hours postpartum for MFG RNA-seq, and we could not determine whether IR altered mammary gene expression immediately after childbirth. Furthermore, variations in MFG RNA quality represent a potential source of bias, and therefore, we included RIN values as a covariate during RNA-seq data analysis. Moreover, the cell models used are relevant for characterizing secretory differentiation and milk protein expression (67) but have limited utility for in vitro assessment of SA.

In summary, these clinical and cellular studies support a metabolic role of insulin in lactating MECs and highlight that delayed SA in women with IR may be due to aberrant insulin action and mitochondrial dysfunction.

## Methods

### Sex as a biological variable.

Our lactation study involved women only.

### Participant recruitment.

Women with singleton pregnancies aged ≥18 years and intending to breastfeed were recruited at 36 weeks’ gestation as part of the Investigating hormones triggering the onset of sustained lactation (INSIGHT) study at the John Radcliffe Hospital (Supplemental Methods) (18). All study visits were undertaken by research practitioners experienced with providing breastfeeding support. Participants were recruited April 2023–December 2025.

### Study visits and sample collection.

Baseline maternal characteristics were obtained during participant interviews and from health records at the time of recruitment. Participant BMI was assessed as part of routine care at 8–10 weeks’ gestation during the initial antenatal clinic visit. This involved measurement of weight and height using calibrated electronic scales and a wall-mounted stadiometer. Baseline blood samples were obtained from pregnant women at 36 weeks’ gestation. After childbirth, all breastfeeding participants had 1 or more study visits during days 1–5 postpartum (Supplemental Table 1). At each study visit, nonfasting serum and lithium heparin plasma was obtained ≥2 hours after the last breastfeed and immediately prior to the next breastfeed. Colostrum and milk samples were collected using a wearable electric breast pump (Elvie) or by hand expression. Samples were transported chilled to the laboratory and processed <2 hours after collection. Serum samples were centrifuged at 1,600*g* for 10 minutes at room temperature and used freshly for the analysis of prolactin, progesterone, and insulin. Lithium heparin samples underwent centrifugation at 1,600*g* for 10 minutes at 4°C, and plasma was stored at –80°C prior to further analyses. Colostrum and milk samples were either used freshly for electrolyte measurements and isolation of MFG RNA (details below) or stored at –80°C prior to further analyses.

### Breast fullness assessments.

Participants recorded their breast fullness daily during postpartum days 1–5 as an indicator of lactation onset after childbirth. Breast fullness was assessed using a validated scale of 1–5, where 1 = no change following delivery, 3 = noticeably fuller, and 5 = uncomfortably full (10, 18).

### Hormone and biochemical analyses.

Serum prolactin, progesterone, insulin, and C-peptide were analyzed by immunoassay on the Architect platform (Abbott Laboratories); plasma glucose was analyzed by hexokinase enzymatic assay on the Architect platform (Abbott Laboratories); and serum high-sensitivity CRP was analyzed by immunoturbidimetric assay on the Alinity ci-series platform (Abbott Laboratories) at the Department of Clinical Biochemistry, John Radcliffe Hospital. Plasma leptin and adiponectin were analyzed by immunoassay on the AutoDELFIA analyzer (Revvity) at the Core Biochemical Assay Laboratory, Cambridge Biomedical Research Centre. Sodium and potassium concentrations were assayed in freshly collected colostrum and milk using LAQUAtwin Na^+^ and K^+^ ion meters (Horiba). Lactose was analyzed in stored colostrum and milk using the K-LACGAR enzymatic assay (Megazyme), as reported (74). Spike and recovery studies involved dissolving lactose, NaCl, and KCl (all from Sigma-Aldrich) in distilled H_2_O prior to addition to early postpartum milk samples to generate spike concentrations of 50, 100, and 150 mmol/L for lactose; 16, 32, and 64 mmol/L for Na^+^; and 10, 20, and 40 mmol/L for K^+^. Lactose, Na^+^, and K^+^ were assayed in triplicate in neat and spiked milk samples. The following equation was used to calculate recovery at each spike concentration: %recovery = ([spiked sample] – [neat sample]/[added analyte]) × 100.

### HOMA.

The HOMA2 (v2.2.4) calculator was used to assess β cell function, insulin sensitivity, and IR (20, 21). Fasting plasma glucose and serum C-peptide values measured during postpartum days 3–28 were used to calculate these indices.

### MFG RNA extraction for transcriptomic analysis.

Colostrum and milk (0.5–2 mL volume samples) were centrifuged at 16,000*g* for 10 minutes at 4°C to obtain the lipid fraction and remove cells (15). The lipid fraction was transferred to a 1.5 mL RNase-free tube and stored in QIAzol Lysis Reagent (QIAGEN) at –80°C until extraction using the RNeasy Plus Universal Mini Kit (QIAGEN).

### Milk lipid cell isolation for flow cytometry.

Colostrum and milk (0.5–2 mL volume samples) were centrifuged at 16,000*g* for 10 minutes at 4°C to obtain the MFG fraction (15). This was transferred to a 1.5 mL tube containing PBS and centrifuged at 400*g* for 5 minutes at 4°C to obtain a cell pellet. Cells were resuspended in flow cytometry buffer (PBS with 0.5% BSA) containing 1:10,000 DRAQ5 (Thermo Fisher Scientific) to label nuclei and 1:100 anti-CD45 monoclonal antibody conjugated to eFluor450 (HI30 48-0459-42; Thermo Fisher Scientific) to label CD45^+^ immune cells. Cells were incubated in this solution for 40 minutes on ice, washed once with PBS, and then fixed with 4% formaldehyde for 10 minutes on ice. Cells were resuspended in 1 mL of flow cytometry buffer following a PBS wash and stored at 4°C prior to flow cytometry (Supplemental Methods).

### Mammary cell culture.

The human MCF-12A cell line (ATCC) was maintained in DMEM/F12 medium (Life Technologies) supplemented with 5% horse serum (Life Technologies), 1.72 μM insulin (Sigma), 3.2 nM EGF (Sigma), 1.4 μM hydrocortisone (STEMCELL Technologies), 1.2 nM cholera toxin (Sigma), and 100 U/mL penicillin/streptomycin (Bio-Techne), referred to as complete medium (44). The murine HC11 cell line (ATCC) was maintained in RPMI 1640 medium (ATCC modification, Life Technologies) supplemented with 10% FBS (Sigma), 860 nM insulin (Sigma), 1.65 nM EGF (Sigma), and 100 U/mL penicillin/streptomycin (Bio-Techne), referred to as complete medium. Cells were tested for mycoplasma infection and used for experiments at passage number ≤ 6.

### Insulin stimulation and transcriptomic analysis of cultured mammary cells.

MCF-12A and HC11 cells were cultured in differentiation media on Matrigel-coated and uncoated Transwell inserts, respectively, to induce β-casein expression (Supplemental Methods). Cells were then cultured overnight in insulin-free differentiation media. MCF-12A cells were treated with 0, 10, or 100 nM insulin for 1, 4, and 8 hours; MCF-12A cells transfected with Akt siRNA (Supplemental Methods) were treated with 0 or 100 nM insulin for 1, 4, and 8 hours; and HC11 cells were treated with 0 or 100 nM insulin for 8 hours. Following insulin treatment, cells were washed with ice-cold PBS, and total RNA was extracted using the RNeasy Micro Kit (QIAGEN) for bulk RNA-seq (Supplemental Methods). Transcriptomic studies involved *n* = 3–5 biological replicates (3–5 individual wells) per treatment and time point.

### Bulk RNA-seq.

RNA-seq was performed by Azenta Life Sciences (Supplemental Methods). Sequencing data were analyzed using Omics Playground (BigOmics Analytics) (Supplemental Methods).

### Insulin-Akt interaction model.

DESeq2 was used to model the insulin-Akt interaction in MCF-12A cells using the formula: ~ Akt + insulin + Akt:insulin, which denotes that gene expression is modeled as a function of Akt, insulin, and their interaction (51). Genes significant for this interaction were categorized as enhanced or attenuated. Enhanced genes were defined as those in which Akt knockdown amplified the effect of insulin, with fold-changes in the same direction (adjusted *P* < 0.05). Attenuated genes were defined as those in which Akt knockdown reduced the effect of insulin or reversed the direction of the fold-change (adjusted *P* < 0.05).

### Cluster analysis of DEGs in MCF-12A cells.

The temporal expression trajectories of 2,367 DEGs with log_2_ fold-change (insulin vs. control) > 1 at any time point were clustered using the R package TMixClust (Supplemental Methods) (50).

### Gene network analysis.

TFs interacting with mitochondrial genes were determined in silico using the ChIP-Atlas (64) and curated ChIP-seq data from lactating mouse mammary gland (Gene Expression Omnibus [GEO] accessions GSM7282350, GSM2577617, GSE231439) and mouse skeletal muscle (gastrocnemius and soleus) (GEO accessions GSE230547, GSE104155, GSM6766609) (Supplemental Methods).

A mammary gland–specific interaction network of inflammatory genes was generated using HumanBase (Supplemental Methods) (75).

### RNA extraction for RT-qPCR.

RNA was extracted using the RNeasy Micro Kit (QIAGEN). RNA concentration and purity were measured using a NanoDrop 1000 spectrophotometer (LabTech International), and samples with A260/A280 ratio 1.7–2.0 were selected for analysis. Total RNA (1 μg) was reverse-transcribed into cDNA using the High-Capacity cDNA Reverse Transcription Kit (Thermo Fisher Scientific). Synthesized cDNA was either stored at –20°C or diluted (1:15) in RNase-free water for amplification. Gene expression was assessed by qPCR using predesigned primers (Supplemental Methods). The qPCR studies involved *n* = 3–5 biological replicates (3–5 individual wells). Samples were analyzed in triplicate (*n* = 3 technical replicates) (Supplemental Figure 17).

### Insulin-stimulated Akt phosphorylation.

Cells were seeded onto Transwell inserts in a 6- or 12-well plate and differentiated to β-casein–expressing cells (Supplemental Methods). Cells were then cultured overnight in insulin-free differentiation media and refreshed with the same medium 2 hours prior to insulin treatment to remove autocrine factors that may induce Akt phosphorylation. Cells were treated with 0, 10, or 100 nM insulin for up to 8 hours; washed with ice-cold PBS; and lysed with RIPA buffer (150 mM NaCl, 1.0% IGEPAL CA-630, 0.5% sodium deoxycholate, 0.1% SDS, 50 mM Tris, pH 8.0, Bio-Techne) containing protease inhibitors (Thermo Fisher Scientific) and phosphatase inhibitors (Stratech Scientific). Cells were lysed on ice for 30 minutes with vortexing every 10 minutes, followed by centrifugation at 16,000*g* for 20 minutes at 4°C to remove insoluble debris. Protein concentrations were determined by Pierce rapid gold BCA protein assay kit (Thermo Fisher Scientific). The Akt phosphorylation studies involved *n* = 3–6 biological replicates (3–6 individual wells) per treatment and time point. Samples were analyzed in duplicate (*n* = 2 technical replicates) (Supplemental Figure 17).

### Mitochondrial imaging.

Confocal imaging involved HC11 cells being seeded in 8-well chamber microscopy slides (Ibidi) at a density of 20,000 cells/well. Cells were grown in insulin-free medium containing 0.1 μM dexamethasone and 1 μg/mL prolactin for 48 hours to near confluence, then treated with insulin (0, 10, 100, 1,000 nM) for 24 hours, labeled with PK Mito Orange (Spirochrome) diluted in treatment media (1:1,000), and incubated for 30 minutes at 37°C and 5% CO_2_ prior to imaging (Supplemental Methods). Mitochondrial morphology and volume analysis was performed using the ImageJ plug-in Mitochondria Analyzer (Supplemental Methods). Transmission electron microscopy mitochondrial imaging involved HC11 cells being seeded onto poly-l-lysine–coated sapphire discs in 24-well plates (Supplemental Methods).

### Cell respiration.

OCR and ECAR were assessed using MitoXpress Intra Intracellular oxygen assay and pH-Xtra Glycolysis Assay, respectively (Agilent Technologies) (Supplemental Methods).

### Statistics.

Analysis of experimental data, with the exception of the transcriptomic datasets, was conducted using Prism (v.10.4.2) (GraphPad Software). Participant categorical variables (e.g., parity, comorbidities, delivery mode, and type of infant feeding) were assessed using χ^2^ test and participant continuous variables (e.g., age, BMI, and infant birthweight) by 1-way ANOVA with Dunnett’s multiple comparisons test. All hormone, biochemical, and cellular data were assessed by unpaired 2-tailed *t* test, 1-way or 2-way ANOVA involving Dunnett’s or Tukey’s multiple comparisons tests, respectively, and also using Kruskal-Wallis test for nonparametric data. Associations between continuous variables was evaluated using simple linear regression. Data for continuous variables are shown as mean ± SEM. Data for categorical variables are shown as numbers and percentages. A *P* value less than 0.05 was considered significant for all analyses.

### Study approval.

Ethical approval was granted by the East of England — Cambridgeshire and Hertfordshire Research Ethics Committee, United Kingdom (REC No. 20/EE/0172). Written informed consent was received from all participants.

### Data availability.

The raw data for the main figure and supplemental figures and tables are provided in the accompanying Supporting Data Values file. All RNA-seq data are deposited in the GEO database (accession GSE342640).

## Author contributions

FMH, XM, TE, and HR designed experiments. XM, TE, HR, MM, AF, BRD, GP, and ICF conducted experiments. XM, TE, HR, MM, AF, BRD, IRH, and KEJ collected and analyzed data. CLD and JFMC advised on data analyses. FMH, LB, and KEJ supervised data analysis. FMH, XM, TE, HR, and RVT wrote the manuscript, integrating feedback from all coauthors.

## Conflict of interest

See supplemental materials for conflicts of interest.

## Funding support

- Larsson-Rosenquist Foundation Oxford Centre for the Endocrinology of Human Lactation (LRF-OCEHL) and the UC San Diego Mother-Milk-Infant Center of Research Excellence (MOMI CORE) by the Family Larsson-Rosenquist Foundation (FLRF).
- BRD by an FLRF doctoral fellowship.

## Supplementary Material

Supplemental data

Unedited blot and gel images

Supplemental tables 1-22

Supporting data values

## Figures and Tables

**Figure 1:**
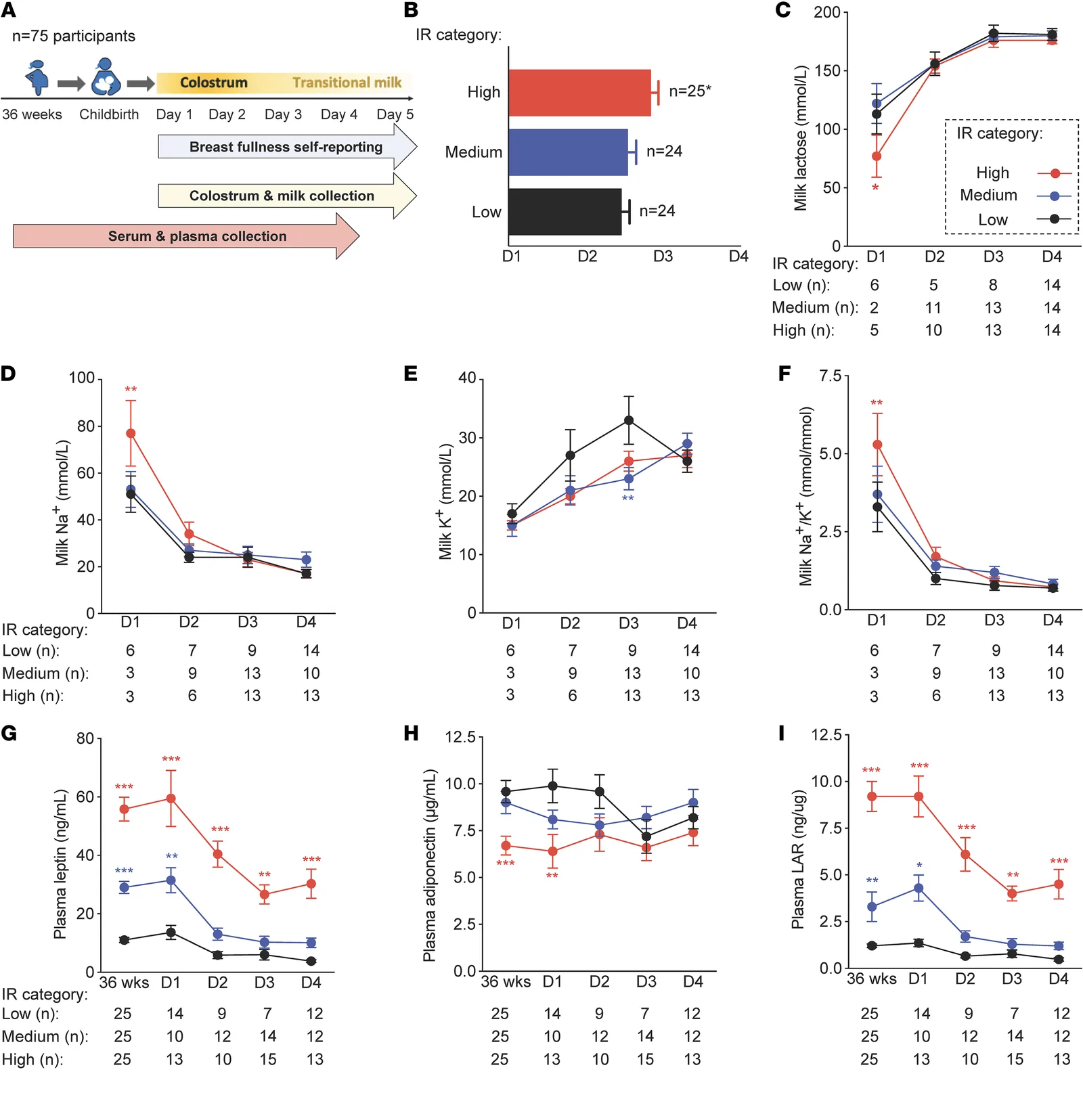
Association of IR with the timing of SA and circulating hormone concentrations. (**A**) Study protocol. (**B**) Timing of SA as assessed by participant daily self-reporting of breast fullness. *P < 0.05 compared with medium and low IR groups (χ^2^ test). (**C**–**E**) Milk concentrations of (**C**) lactose, (**D**) Na^+^, and (**E**) K^+^. (**F**) Milk Na^+^/K^+^ ratio. *P < 0.05, **P < 0.01 (2-way ANOVA). (**G**–**I**) Plasma concentrations of (**G**) leptin and (**H**) adiponectin and (**I**) leptin/adiponectin ratio (LAR). *P < 0.05, **P < 0.01, ***P < 0.001 (2-way ANOVA). All participants were stratified into low (black), medium (blue), or high IR (red) categories based on plasma LAR values. Sample sizes are shown below graphs in **C**–**I**. Day 1 samples were collected at 21 ± 1.0 hours postpartum, day 2 samples at 46 ± 1.0 hours postpartum, day 3 samples at 71 ± 1.0 hours postpartum, and day 4 samples at 96 ± 1.0 hours postpartum. No significant differences in sample collection time were observed between low, medium, and high IR groups (Kruskal-Wallis test). Red and blue asterisks represent *P* values for high vs. low IR group comparisons and medium vs. low IR group comparisons, respectively. D, postpartum day.

**Figure 2:**
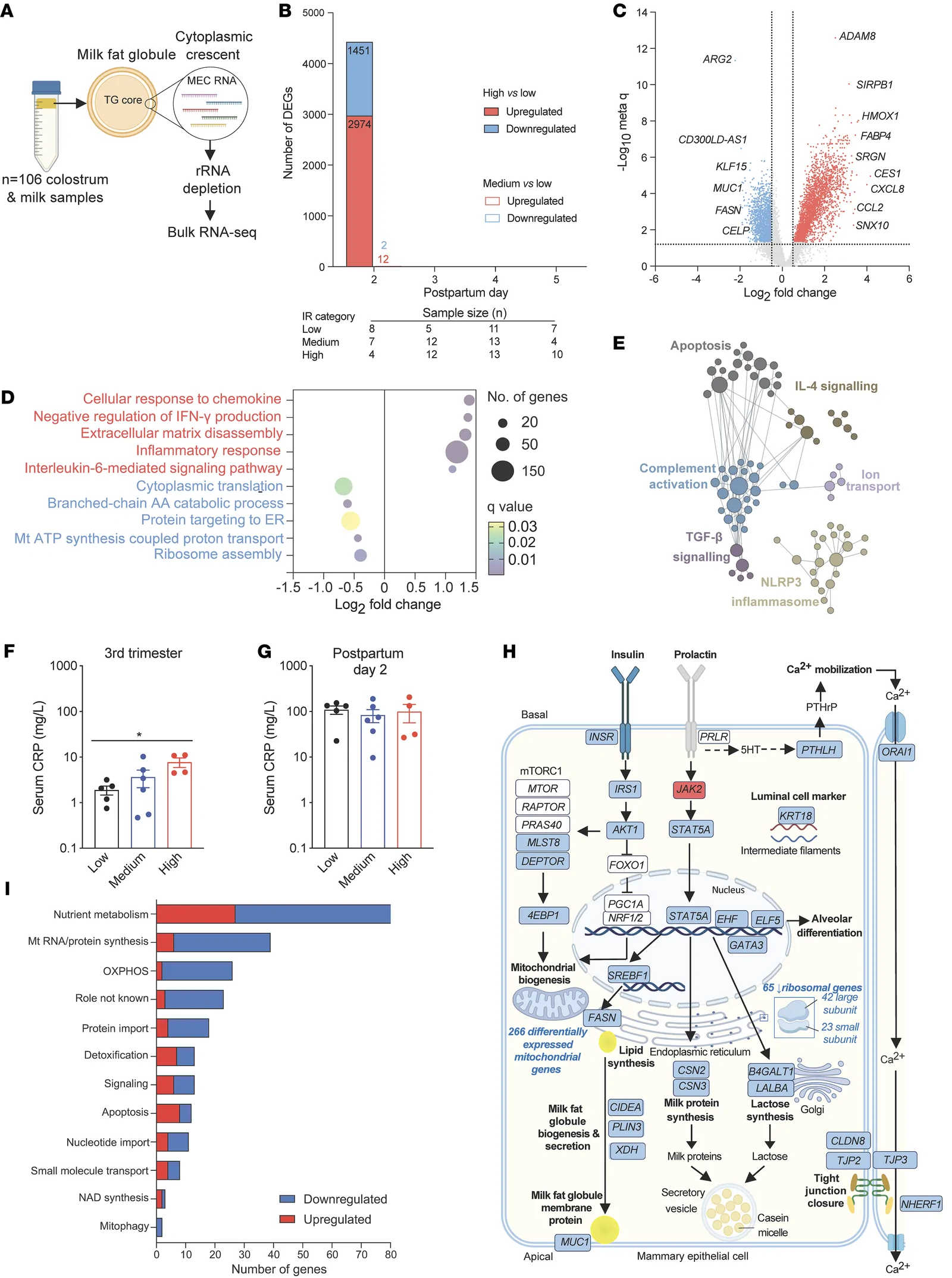
Association of IR with the MFG transcriptome during SA. (**A**) MFG RNA-seq protocol. TG, triglyceride; MEC, mammary epithelial cell. (**B**) Number of differentially expressed genes (DEGs) from high vs. low and medium vs. low IR groups on postpartum days 2–5. Day 2 samples were collected at 45 ± 1.5 hours postpartum, day 3 samples at 72 ± 1.3 hours postpartum, day 4 samples at 96 ± 1.2 hours postpartum, and day 5 samples at 120 ± 1.4 hours postpartum. No significant differences in sample collection time were observed between low, medium, and high IR groups (Kruskal-Wallis test). (**C**) Volcano plot showing upregulated (red) and downregulated (blue) genes from high vs. low IR groups on postpartum day 2. Horizontal and vertical dashed lines indicate meta-*q* = 0.05 and log_2_ expression fold-change = 0.5, respectively. (**D**) GSEA showing top upregulated (red) and downregulated (blue) processes from high vs. low IR groups on postpartum day 2. Circle size represents number of genes. Circle color represents FDR-adjusted *P* value (meta-*q*) obtained from fast GSEA (FGSEA) and single-sample GSEA (ssGSEA). (**E**) Inflammatory gene network upregulated in the high IR group on postpartum day 2. Network comprises nodes representing genes and edges representing functional gene interactions. Node size corresponds to number of connections to other nodes. (**F** and **G**) Serum C-reactive protein (CRP) measured at (**F**) 36 weeks’ gestation and (**G**) postpartum day 2 in low, medium, and high IR groups. *P < 0.05 (1-way ANOVA). (**H**) Schematic of lactating MEC showing processes altered in the high IR group on postpartum day 2. Upregulated (red) and downregulated (blue) genes and proteins are shown. Unaltered genes and proteins are shown in white. (**I**) Mitochondrial processes altered in the high IR group on postpartum day 2. Numbers of upregulated and downregulated genes are shown in red and blue, respectively.

**Figure 3:**
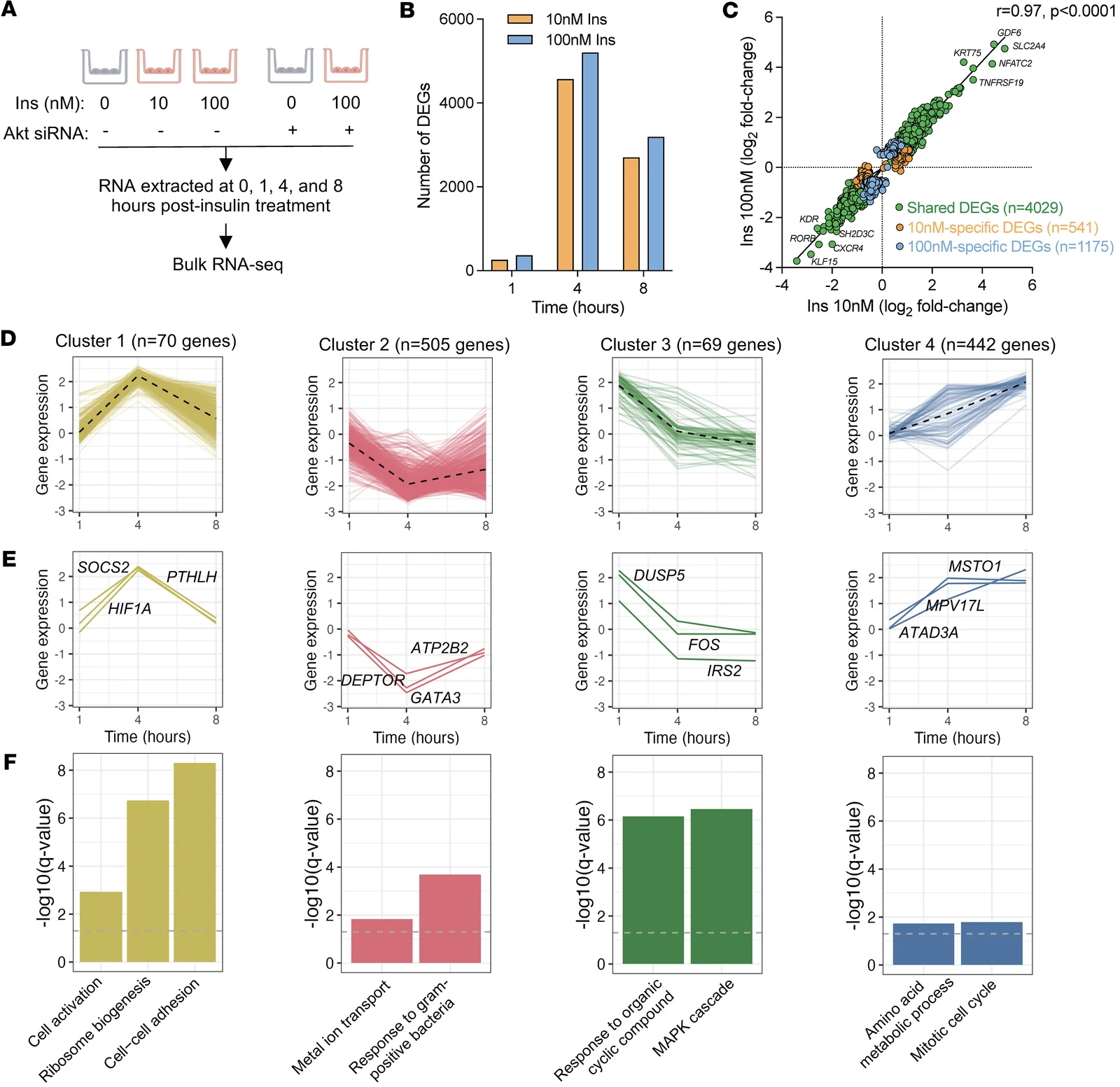
Effect of insulin on the MCF-12A cell transcriptome. (**A**) RNA-seq protocol. (**B**) Number of DEGs at 1, 4, and 8 hours following 10 nM and 100 nM insulin (*n* = 3–5 biological replicates). (**C**) Expression fold-change correlation between DEGs from 10 nM and 100 nM insulin treatments. (**D**) Time-series analysis of 4 gene clusters characterized by transient increase (yellow), transient decrease (red), gradual decrease (green), or gradual increase (blue) in expression following 100 nM insulin treatment. Black dashed lines indicate mean expression. (**E**) Examples of temporal expression trajectories. (**F**) Most significantly enriched Gene Ontology terms in each cluster.

**Figure 4:**
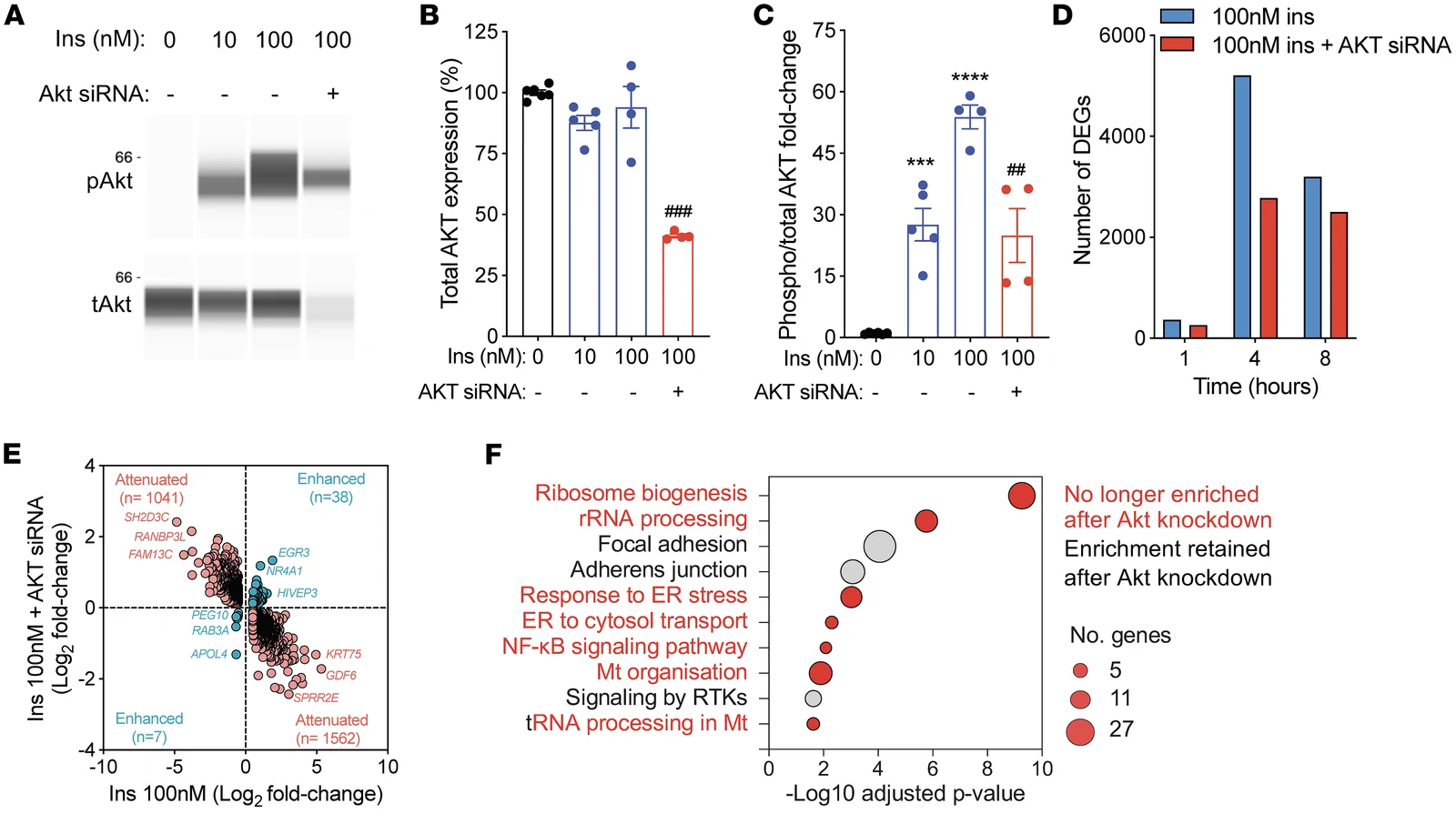
Effect of Akt knockdown on insulin-mediated differential gene expression. (**A**) Capillary protein electrophoresis blot showing phosphorylated and total Akt in MCF-12A cells following 30 minutes of 0, 10, or 100 nM insulin and 100 nM insulin with Akt-targeted siRNA treatments. (**B** and **C**) Quantitative analysis of (**B**) total Akt shown as percentage compared with control (0 nM insulin) and (**C**) p-Akt normalized to total Akt and shown as a fold-change compared with control (0 nM insulin). Data shown as mean ± SEM (*n* = 4–6 biological replicates). ***P < 0.001 and ****P < 0.0001 compared with untreated cells. ^##^*P* < 0.01 and ^###^*P* < 0.001 in cells treated with 100 nM insulin and Akt-targeted siRNA compared with cells treated with 100 nM insulin alone (1-way ANOVA). (**D**) Number of DEGs at 1, 4, and 8 hours following 100 nM insulin ± Akt-targeted siRNA. (**E**) Interaction analysis between Akt expression and 100 nM insulin at 4 hours. Scatterplot shows the relationship between the expression of insulin-induced DEGs that are attenuated (red dots) or enhanced (blue dots) by Akt knockdown. *X* axis shows log_2_ expression fold-change from 100 nM insulin alone, and the *y* axis shows the log_2_ expression fold-change from 100 nM insulin + Akt-targeted siRNA. (**F**) Top enriched biological processes following 100 nM insulin ± Akt-targeted siRNA treatment. Circle size represents number of genes involved per process. Processes no longer enriched after Akt knockdown are shown in red.

**Figure 5:**
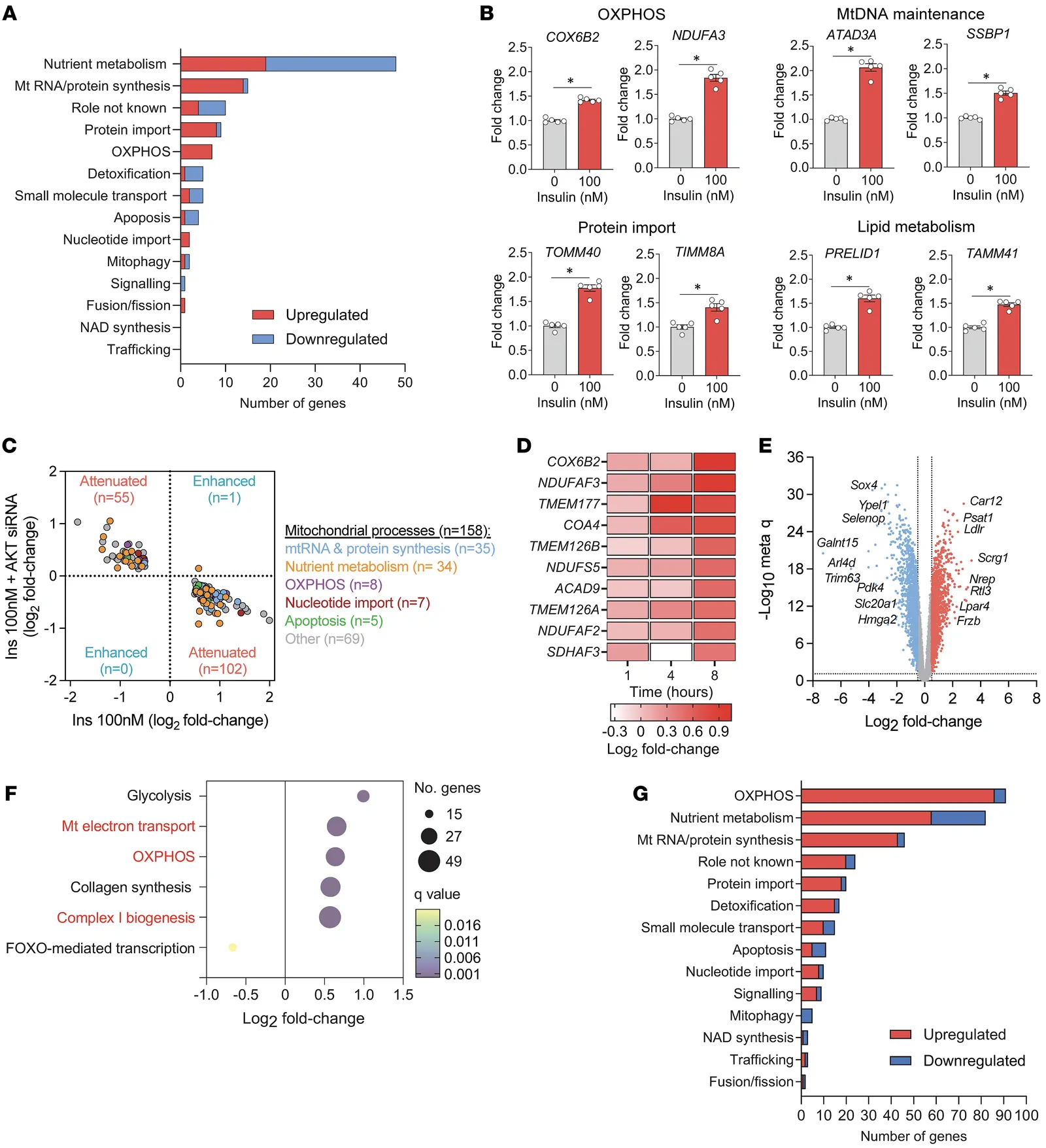
Effect of insulin on mitochondrial gene expression in cultured mammary cells. (**A**) Major mitochondrial processes altered in MCF-12A cells following 100 nM insulin. Numbers of up- and downregulated genes are shown in red and blue, respectively. (**B**) Selected mitochondrial genes altered in MCF-12A cells after 0 nM (gray) or 100 nM (red) insulin. Data shown as mean ± SEM (*n* = 5 biological replicates). *Meta-*q* < 0.05 from DEG analysis. (**C**) Interaction analysis between Akt expression and 100 nM insulin, showing mitochondrial genes with either attenuated or enhanced expression following Akt knockdown in MCF-12A cells treated with 100 nM insulin. Numbers of genes categorized according to pathway annotations are shown (43). Other mitochondrial processes include detoxification, NAD metabolism, dynamics, signaling, mitophagy, and small molecule transport. (**D**) Heatmap of OXPHOS genes upregulated by 100 nM insulin. Each cell represents mean log_2_ fold-change from *n* = 5 biological replicates. (**E**) Volcano plot showing up- (red) and downregulated (blue) genes following 100 nM insulin in HC11 cells. (**F**) Enriched biological processes following 100 nM insulin in HC11 cells. Mitochondrial processes are shown in red. Circle color represents FDR-adjusted *P* value (meta-*q*) obtained from FGSEA and ssGSEA. (**G**) Mitochondrial processes altered in HC11 cells following 100 nM insulin.

**Figure 6:**
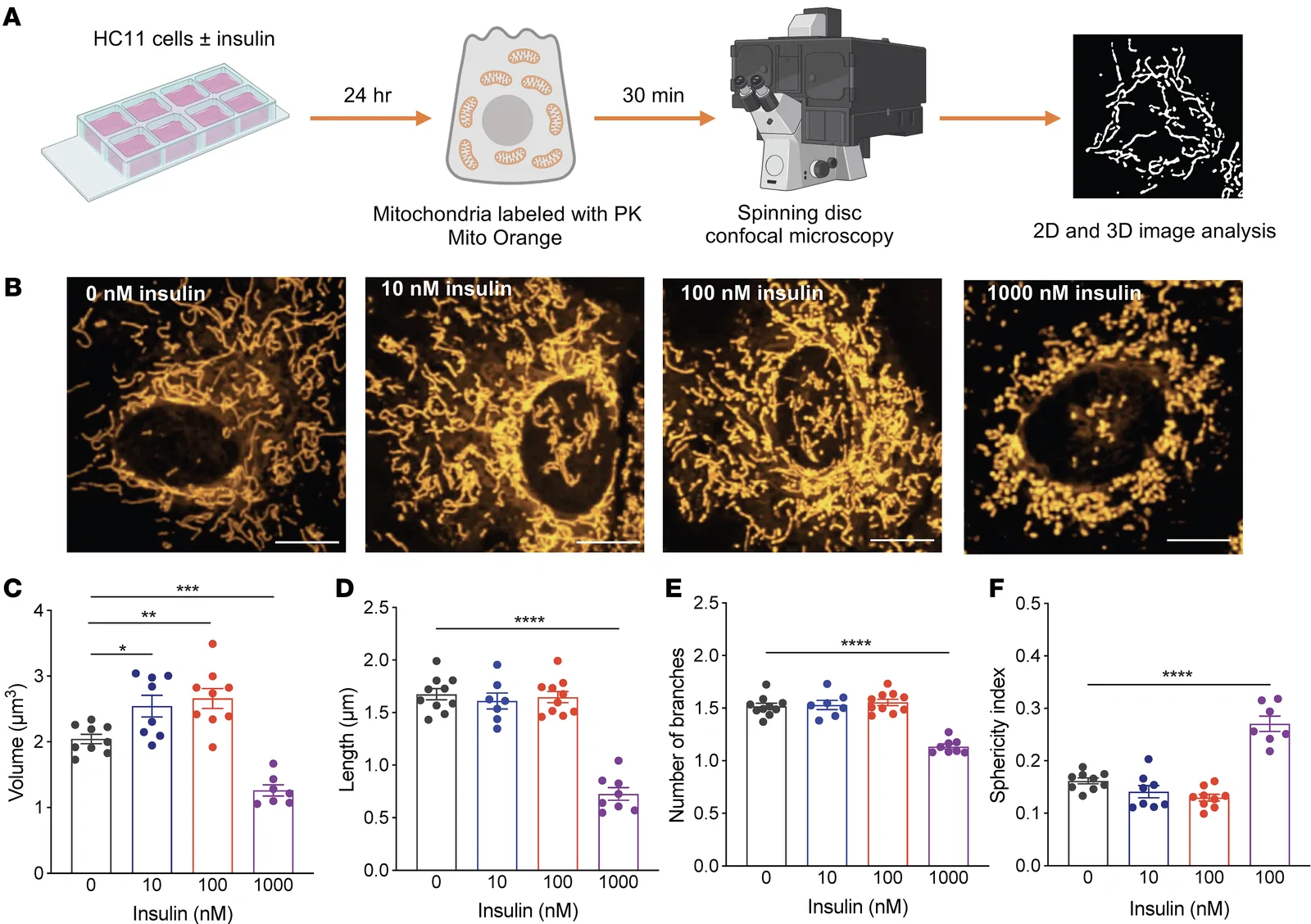
Effect of insulin on mitochondrial volume and morphology in cultured mammary cells. (**A**) Protocol for mitochondrial volume and morphology analysis in HC11 cells. (**B**) Representative images of mitochondria in cells treated with insulin (0, 10, 100, or 1,000 nM) for 24 hours, shown as maximum *z* projections. Scale bar = 10 μm. (**C**–**F**) Quantitative analysis of mitochondrial parameters: (**C**) volume, (**D**) length, (**E**) number of branches, and (**F**) sphericity in insulin-treated cells. On average, 7,000–10,000 mitochondria were analyzed per image from each condition (*n* = 5–10 images per condition). Data in **C**–**F** are shown as mean ± SEM (*n* = 7–10 biological replicates). *P < 0.05, **P < 0.01, ***P < 0.001, ****P < 0.0001 (1-way ANOVA).

**Figure 7:**
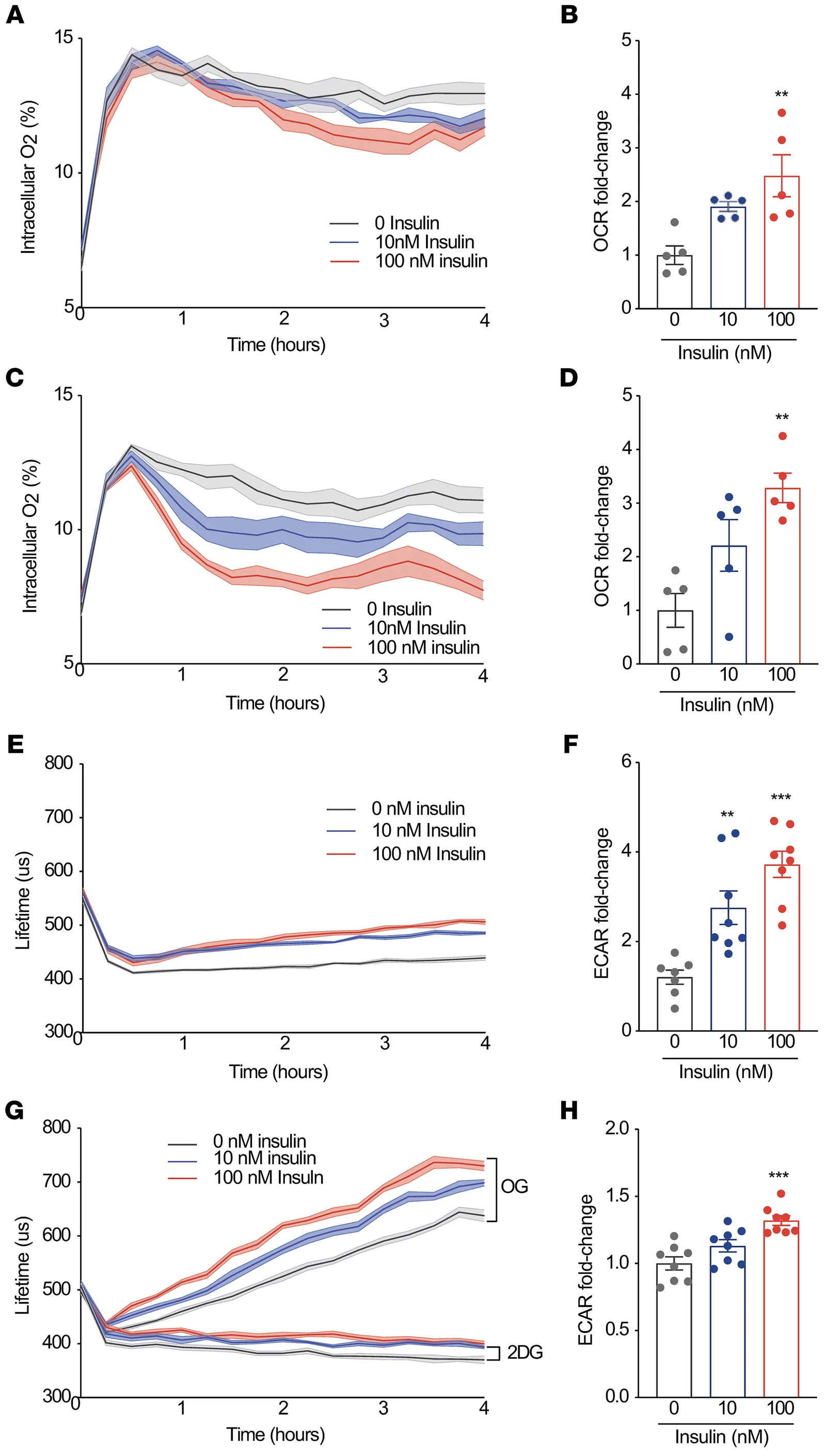
Bioenergetic effects of insulin in cultured mammary cells. (**A**) Changes in basal intracellular O_2_ in response to insulin. (**B**) Basal OCR shown as a fold-change compared with HC11 cells without insulin. (**C**) Changes in intracellular O_2_ in response to insulin and FCCP-induced O_2_ consumption. (**D**) FCCP-induced OCR shown as a fold-change compared with cells without insulin. (**E**) Changes in basal pH in response to insulin, expressed as fluorescence lifetime. (**F**) Basal extracellular acidification rate (ECAR) shown as a fold-change compared with cells without insulin. (**G**) Changes in pH in response to insulin and stimulation or inhibition of glycolysis with oligomycin (OG) or 2-deoxyglucose (2DG), respectively. (**H**) Glycolytic capacity calculated by ECAR after oligomycin addition minus ECAR after 2DG addition. Data in **B**, **D**, **F**, and **H** are shown as mean ± SEM (*n* = 5–8 biological replicates). **P < 0.01, ***P < 0.001 compared with untreated HC11 cells (1-way ANOVA).

**Table 1:**
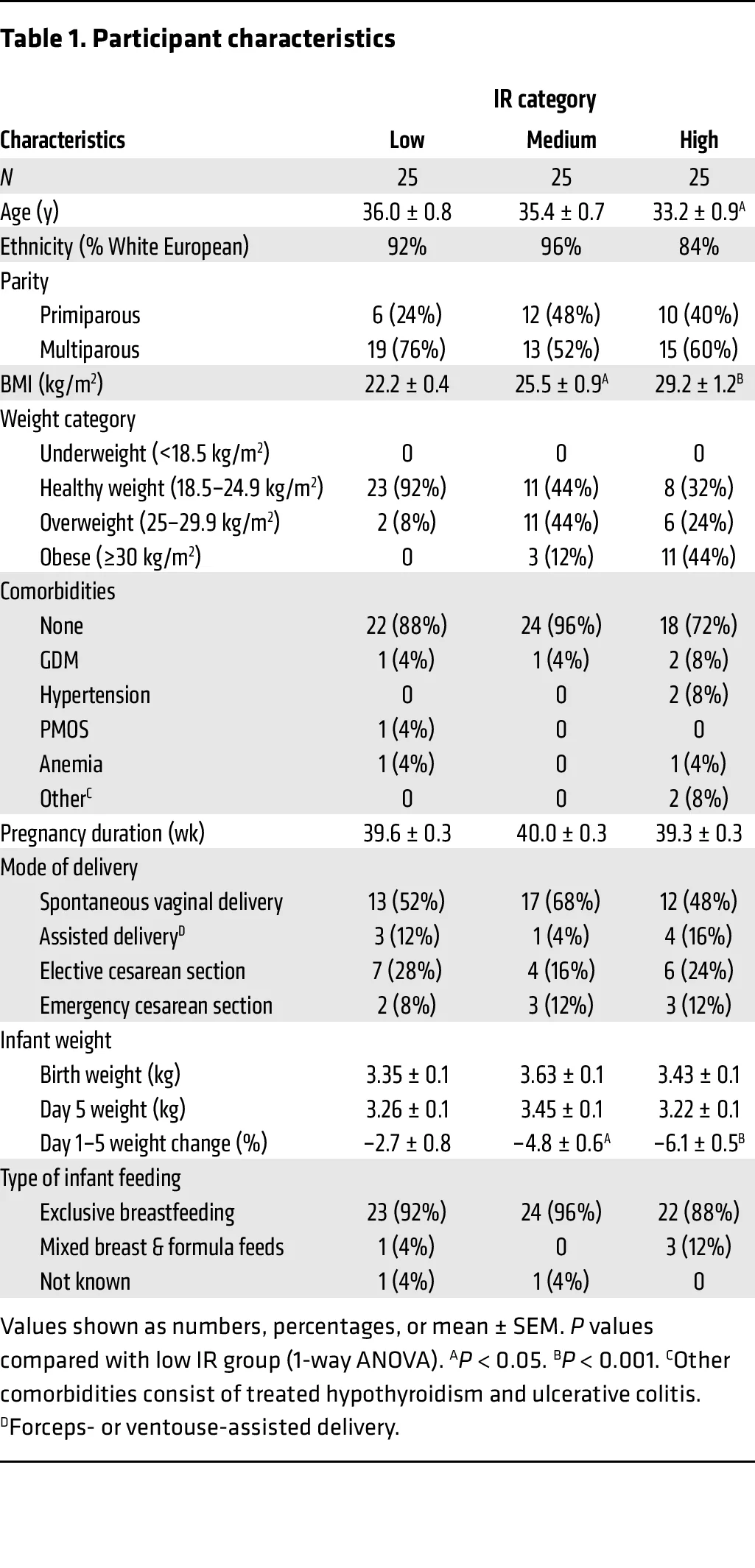
Participant characteristics

## Version 1 08/13/2026

In-Press Preview

## Version 2 10/15/2026

Electronic publication
