# Supplementary material for: Insulin resistance is associated with mammary mitochondrial dysfunction at the onset of human lactation
Source: J Clin Invest. 2026 Aug 13;136(20):e197311. doi: 10.1172/JCI197311 (PMC13668628; doi:10.1172/JCI197311)
Supplement: Supplementary file 1 [file jci-136-197311-s263.pdf]

Supplementary materials:

Supplemental Figures 1-17

Supplemental Methods

Supplemental References

Conflict of Interest: Patent Information

Supplementary Figure 1. Leptin: adiponectin ratio: Cut-off values and association with postpartum homeostasis model assessment, pregnancy BMI and glucose tolerance test

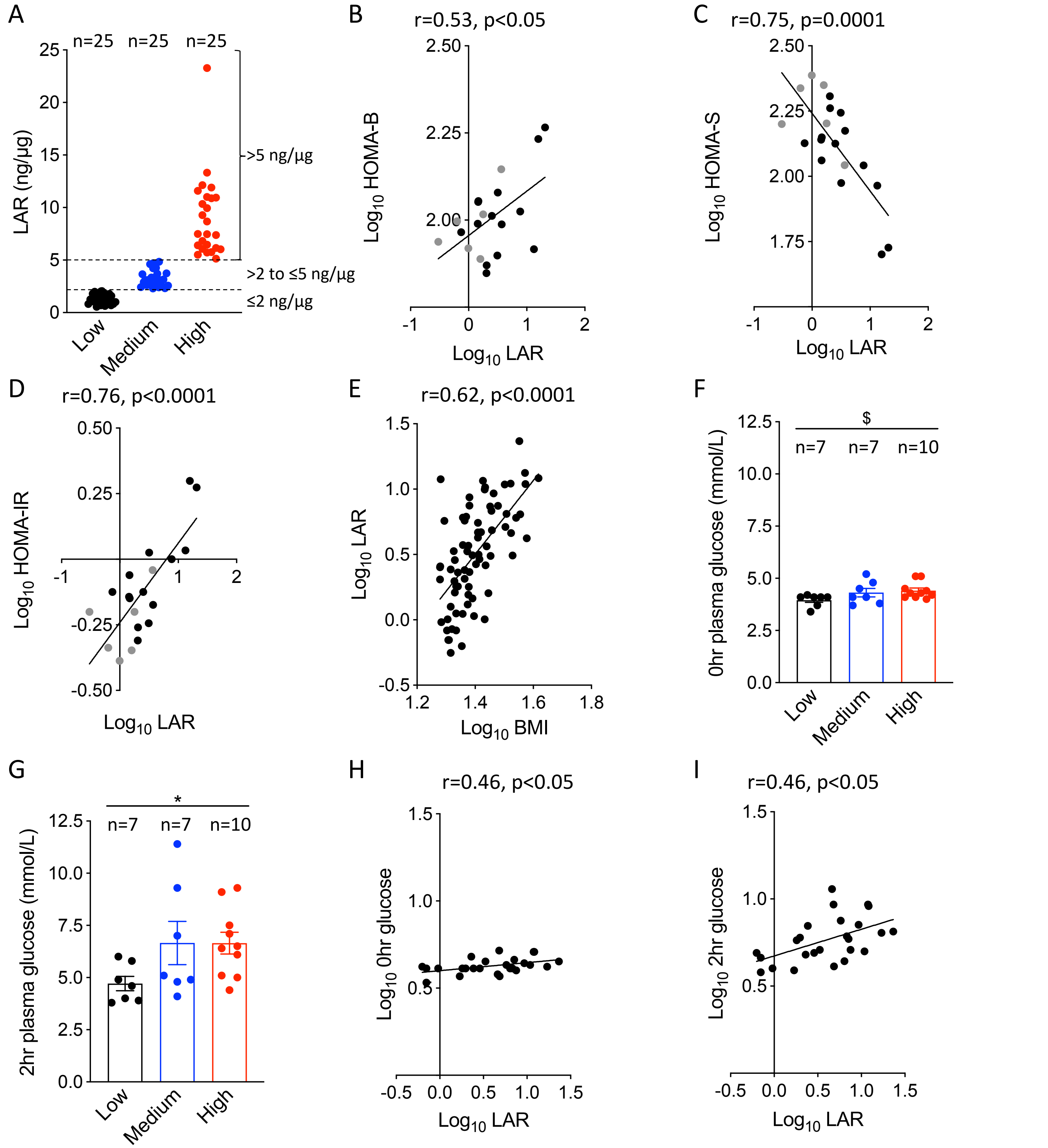

Supplementary Figure 1. (A) Participants (n=75) were divided into low, medium and high insulin resistance (IR) tertiles (n=25 per tertile) using plasma leptin: adiponectin ratio (LAR) cut-off values of: ≤2ng/µg; >2 to ≤ 5ng/µg; and >5ng/µg. **(B-D)** Association of LAR with postpartum homeostasis model assessment (HOMA) for **(B)** β-cell function (HOMA-B); **(C)** insulin sensitivity (HOMA-S); and **(D)** IR (HOMA-IR) in n=20 breastfeeding participants. Grey and black dots indicate HOMA values assessed during postpartum days 3-4 or days 15-28, respectively. **(E)** Association of BMI assessed at 8-10 weeks' gestation with LAR in n=75 breastfeeding participants. **(F-G)** Oral glucose tolerance test plasma glucose values obtained during 24-36 weeks' gestation at **(F)** 0hr and **(G)** 2hr in n=24 breastfeeding participants in low, medium and high IR categories. **(H-I)** Association of LAR with **(H)** 0hr and **(I)** 2hr plasma glucose values. $p=0.06, *p<0.05. Kruskal-Wallis test used for comparison of low, medium and high IR categories.

Supplementary Figure 2. Association of insulin resistance with serum insulin, progesterone and prolactin concentrations.

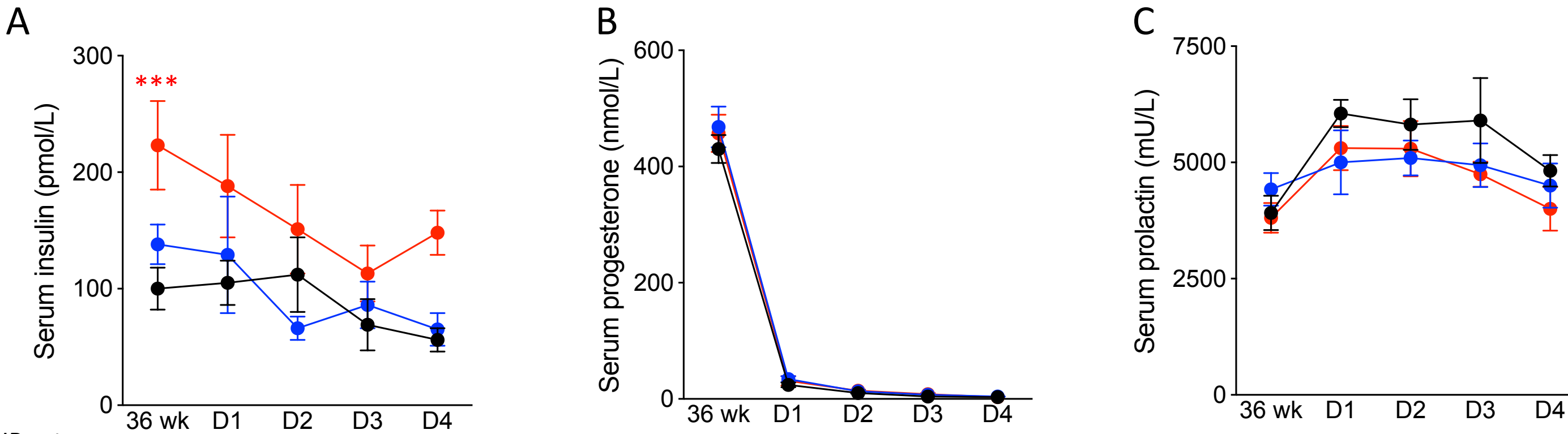

IR category:	A 36 wk	A D1	A D2	A D3	A D4	B 36 wk	B D1	B D2	B D3	B D4	C 36 wk	C D1	C D2	C D3	C D4
Low (n):	22	12	10	8	8	21	11	9	9	8	23	11	11	7	9
Medium (n):	24	6	10	14	8	23	8	11	11	6	24	7	10	15	8
High (n):	25	13	8	15	10	23	13	8	17	11	25	14	9	14	12

Supplementary Figure 2. Change in serum concentrations of: **(A)** insulin; **(B)** progesterone; and **(B)** prolactin during postpartum days 1-4. ***p<0.001 (two-way ANOVA). All participants were stratified into low (black), medium (blue) or high insulin resistance (IR, red) categories based on plasma leptin: adiponectin ratio values. Sample sizes are shown below graphs.

Supplementary Figure 3. Milk fat globule RNA quality

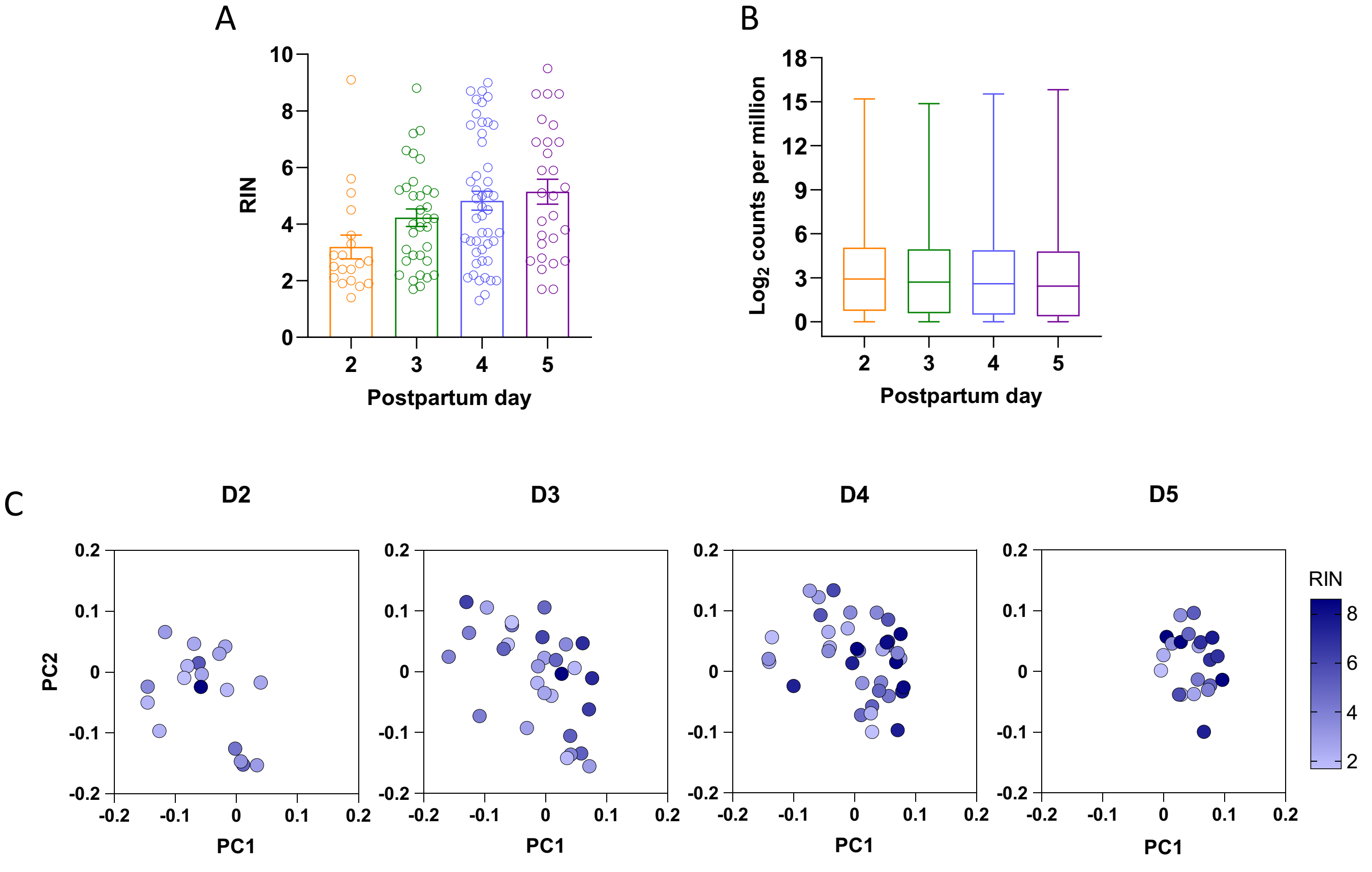

Supplementary Figure 3. (A), Integrity of milk fat globule (MFG) RNA obtained from postpartum days 2-5. RIN, RNA integrity number. Data shown as mean±SEM. **(B)** Sample counts obtained from MFG transcriptomes. Raw counts were normalised for sequencing depth and expressed as $\log_2$ counts per million showing uniform distribution between samples from postpartum days 2-5. Data shown as box plots. **(C)** Principal components analysis of MFG transcriptomes from postpartum days 2-5 according to RIN value. Each dot represents one MFG sample. D, postpartum day.

Supplementary Figure 4. Comparison of milk fat globule gene expression with human and mouse tissues, and assessment of transcript abundance

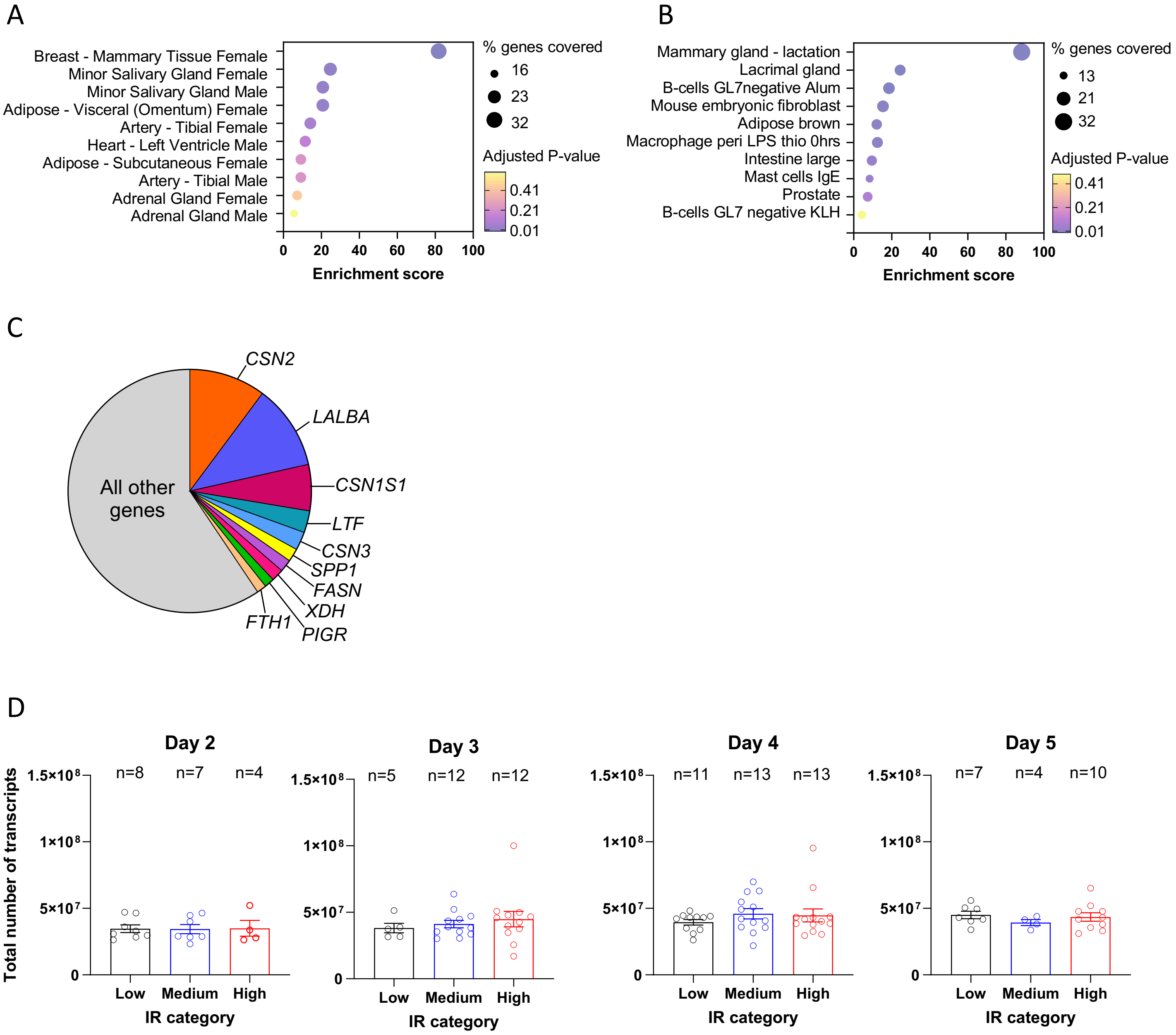

Supplementary Figure 4. (A-B) Enrichment analysis of the 10 most highly expressed genes in the milk fat globule (MFG) transcriptome from days 2-5 postpartum compared with human and mouse tissues from **(A)** Genotype-Tissue Expression (GTEx) and **(B)** Mouse Gene Atlas databases. **(C)** Pie chart showing relative transcript abundance of the 10 most highly expressed MFG genes. **(D)** Total number of MFG transcripts in high (red), medium (blue) and low (black) insulin resistance (IR) groups according to postpartum day.

Supplementary Figure 5. Association of insulin resistance and body mass index with milk fat globule gene expression

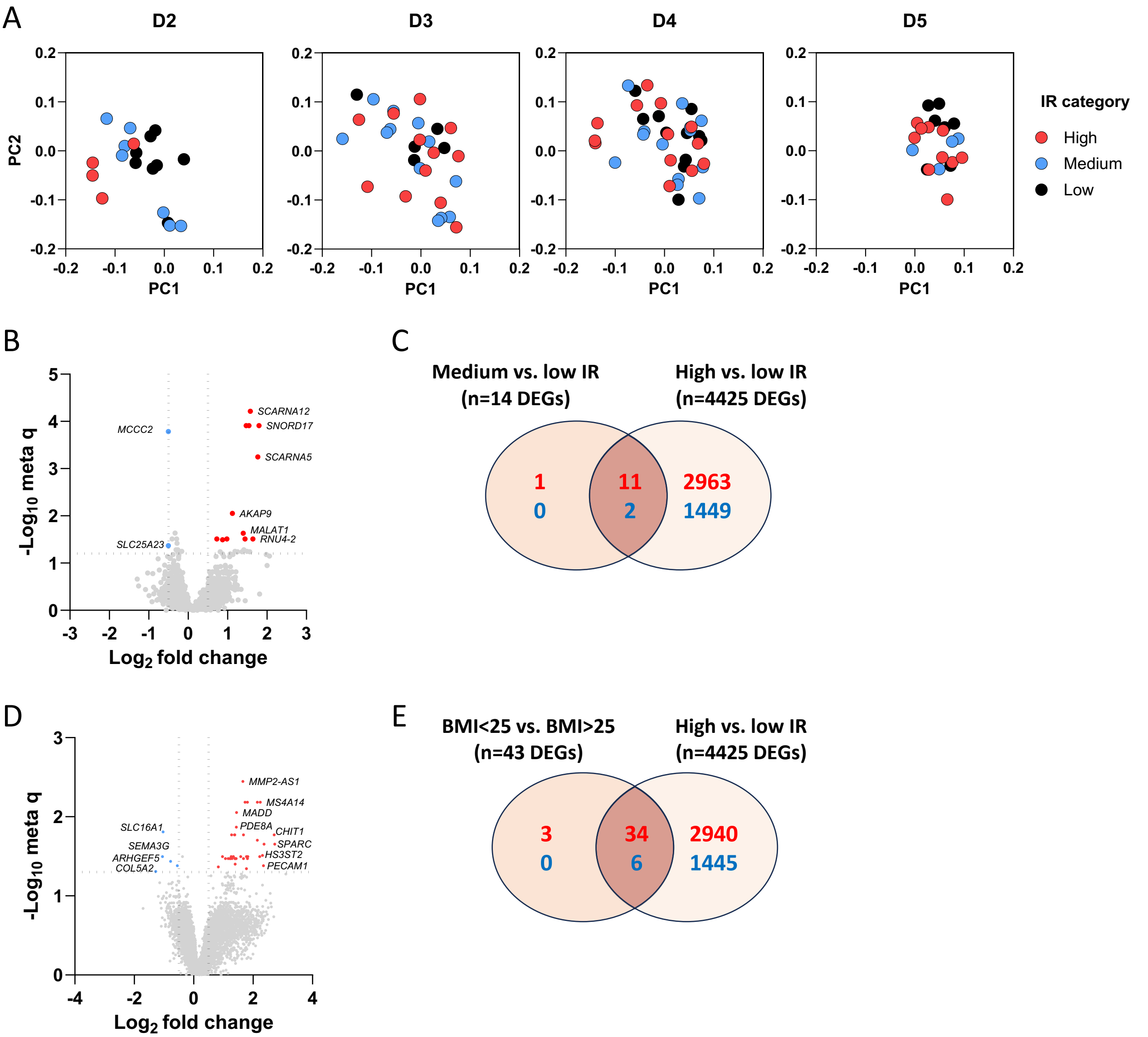

Supplementary Figure 5. (A) Principal components analysis of MFG transcriptomes from postpartum days 2-5. show clustering of samples according to insulin resistance (IR) category on day 2. Each dot represents one MFG sample. **(B)** Volcano plot showing differentially expressed genes (DEGs) obtained from a comparison of medium vs. low IR groups. **(C)** Venn diagram showing the number of overlapping genes obtained from comparison of medium vs. low IR groups with high vs. low IR groups. **(D)** Volcano plot showing DEGs obtained from a comparison of body mass index (BMI) <25 kg/m^2 vs. BMI >25 kg/m^2. **(E)** Venn diagram showing the number of overlapping genes obtained from comparison of BMI <25 kg/m^2 vs. BMI >25 kg/m^2 with high vs. low IR groups. Numbers of upregulated and downregulated genes are shown in red and blue, respectively.

Supplementary Figure 6. Correlations between milk fat globule gene expression and the plasma leptin: adiponectin ratio

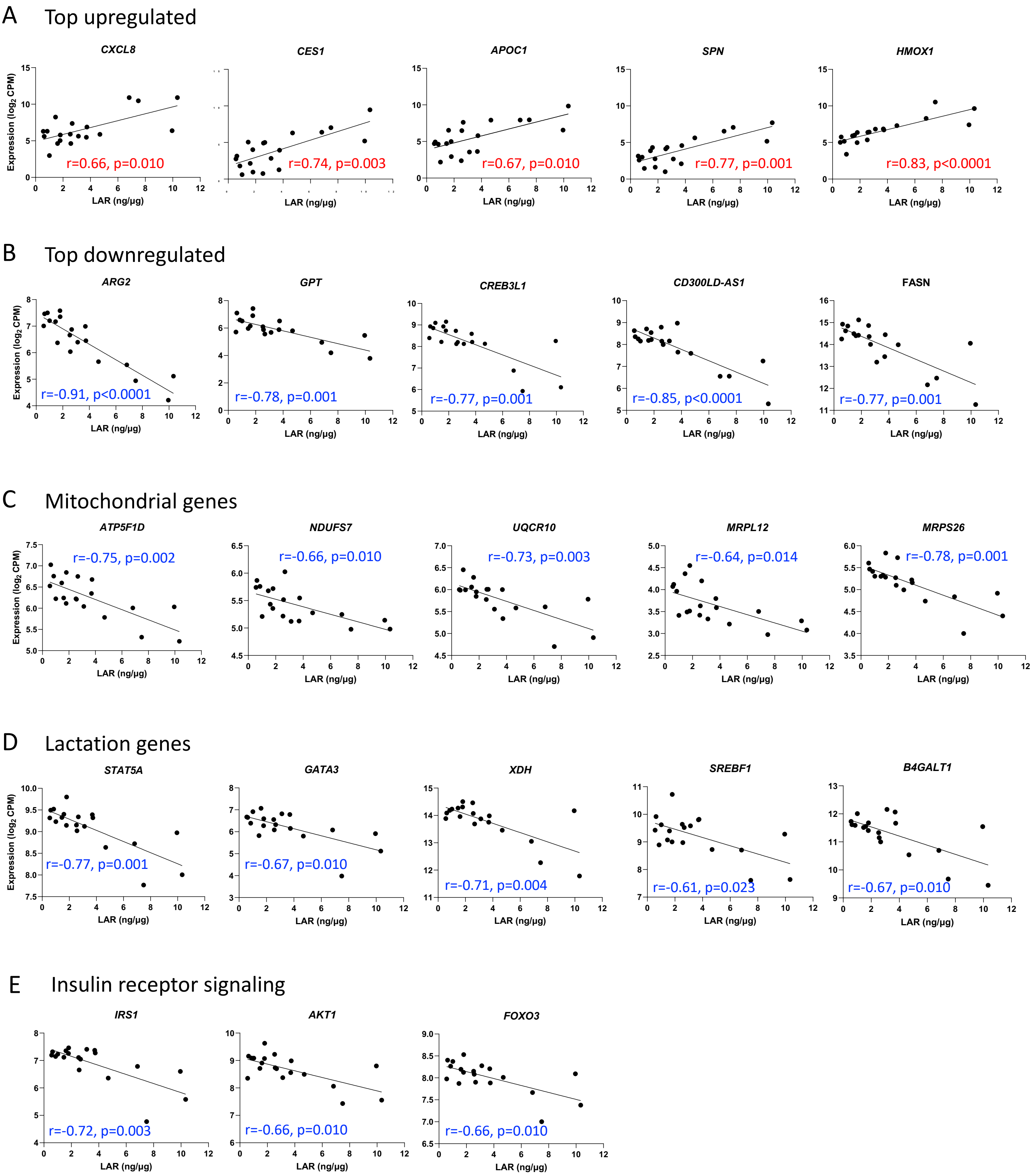

Supplementary Figure 6. Differentially expressed genes are shown from a comparison of high vs. low insulin resistance groups on postpartum day 2. Genes are grouped into the following categories: **(A)** Top upregulated genes; **(B)** Top downregulated genes; **(C)** Mitochondrial genes; **(D)** Lactation genes; and **(E)** Insulin receptor signaling genes. Pearson correlation coefficient (r) and associated p values are shown in each graph. CPM, counts per million.

Supplementary Figure 7. Association of plasma leptin: adiponectin ratio with number of cells in the milk fat globule fraction

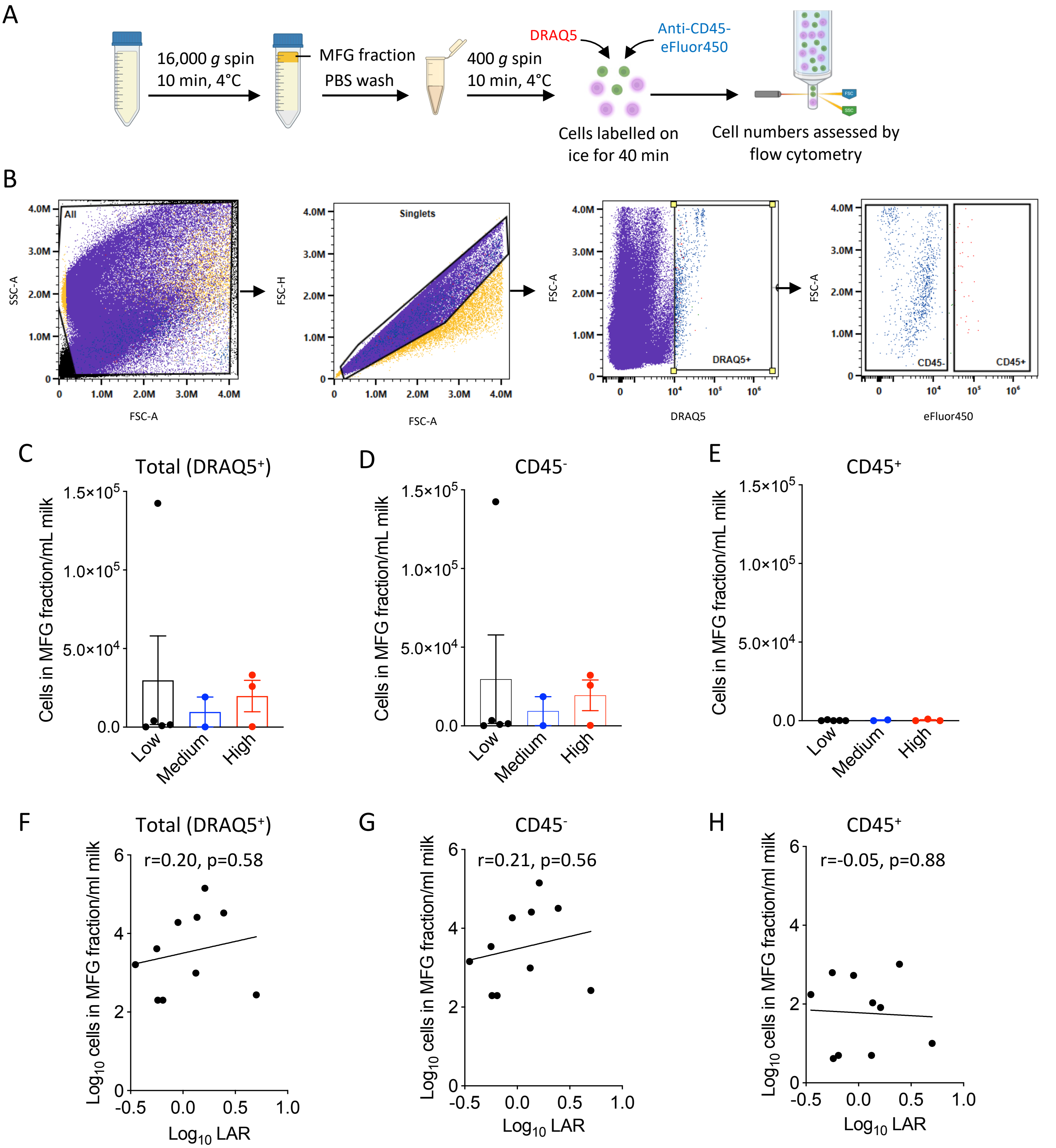

Supplementary Figure 7. (A) Milk processing and flow cytometry protocol. **(B)** Representative dot plots showing flow cytometry gating of cells derived from the milk fat globule (MFG) fraction. FSC-A, forward scatter area; SSC-A, side scatter area; FSC-H, forward scatter height. **(C-E)** Quantification of **(C)** total cells (DRAQ5$^+$), **(D)** CD45$^-$, and **(E)** CD45$^+$ cells obtained from the MFG fraction per mL of milk. **(F-H)** Correlation between leptin-adiponectin ratio (LAR) and **(F)** number of total cells (DRAQ5$^+$), **(G)** CD45$^-$ or **(H)** CD45$^+$ cells. The Pearson correlation coefficient (r) and associated p values are shown.

Supplementary Figure 8. Association of serum C-reactive protein at 36 weeks' gestation with milk fat globule gene expression on postpartum day 2

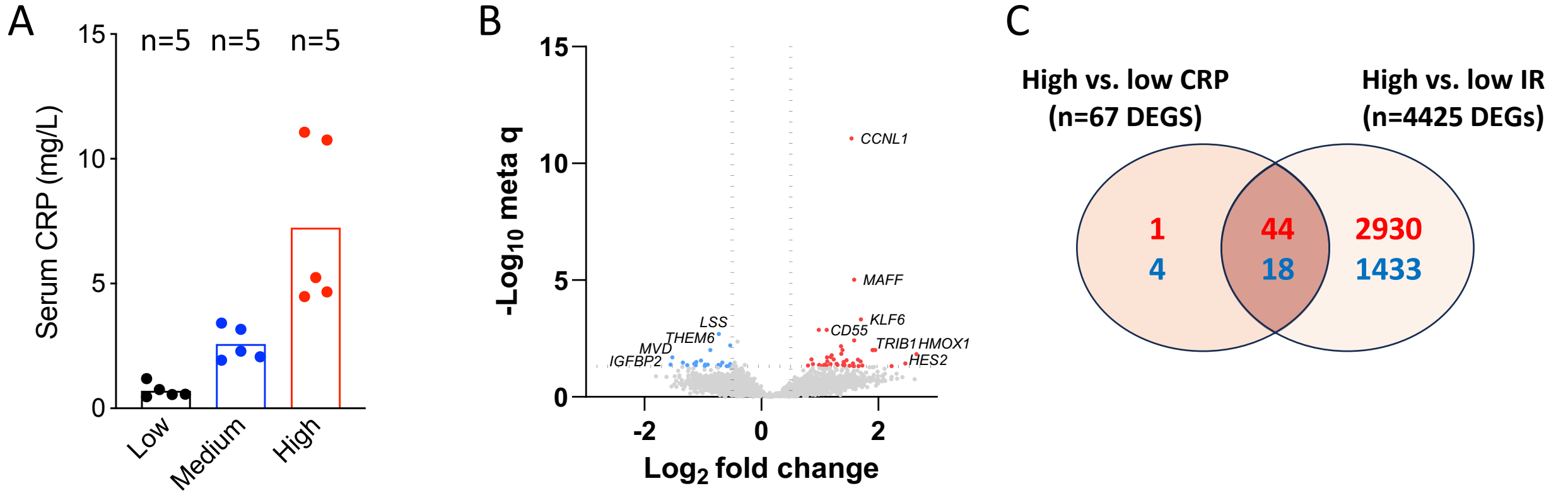

Supplementary Figure 8. (A) Participants (n=15) were divided into low, medium and high tertiles (n=5 per tertile) based on serum CRP values measured at 36 weeks' gestation. **(B)** Volcano plot showing differentially expressed genes (DEGs) obtained from a comparison of postpartum day 2 MFG transcriptomes from participants in high vs. low CRP tertiles. DEGs with log_2 fold-change of ≥0.5 and meta q value of <0.05 are shown in red (upregulated) or blue (downregulated). Grey dots represent genes not meeting the fold-change or significance thresholds. **(C)** Venn diagram showing the number of overlapping genes between participants in the high IR and high CRP groups. Numbers of upregulated and downregulated genes are shown in red and blue, respectively.

Supplementary Figure 9. Effect of Akt knockdown on the RNA expression of AKT1-3 isoforms, beta-casein and insulin receptor in MCF-12A cells

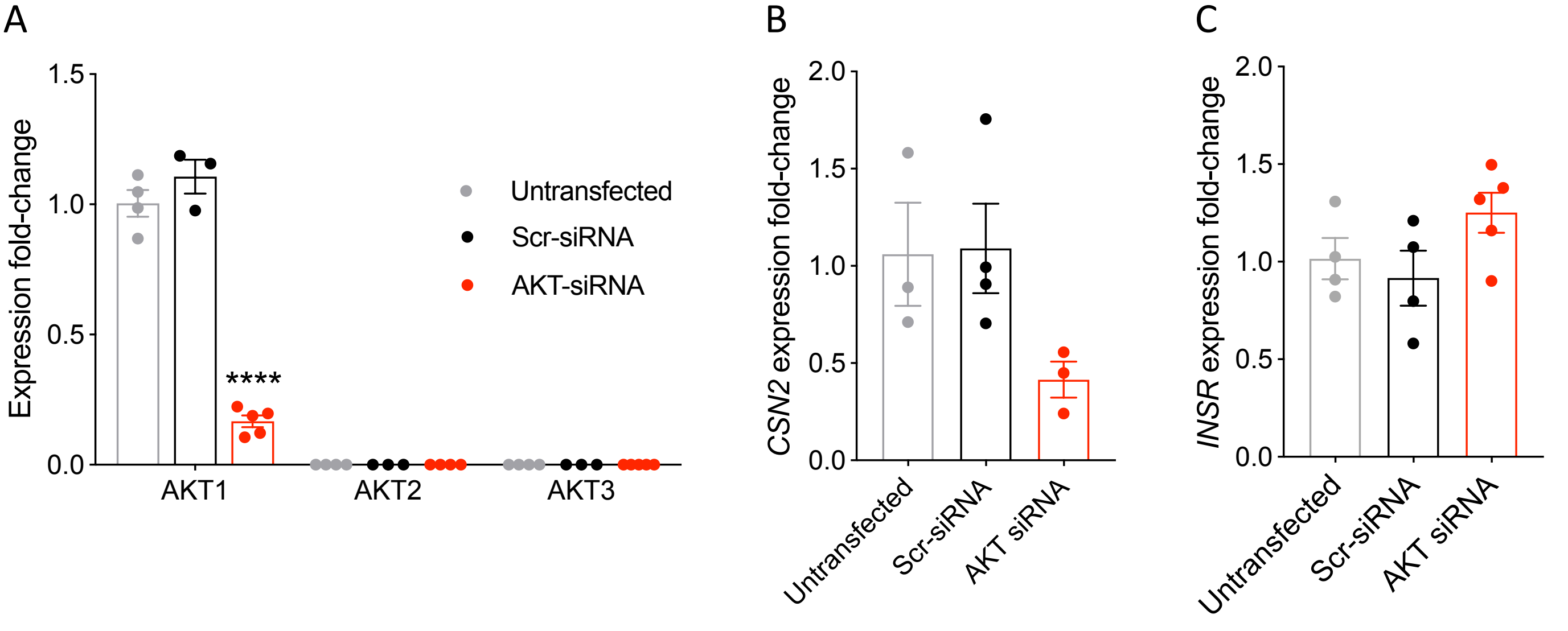

Supplementary Figure 9. RNA expression of **(A)** AKT1-3 isoforms, (B) beta-casein (*CSN2*), and **(C)** insulin receptor *(INSR)* in untransfected MCF-12A cells (grey) or cells transfected with scrambled siRNA (Scr-siRNA, black) or Akt-targeted siRNA (AKT-siRNA, red). Each dot represents an individual well of MCF-12A cells. RNA expression is shown as mean±SEM fold-change compared to untransfected cells. ****p<0.0001. One-way ANOVA was used to compare groups.

Supplementary Figure 10. RNA quality assessment, principal components analysis, and clustering of MCF-12A transcriptomic data

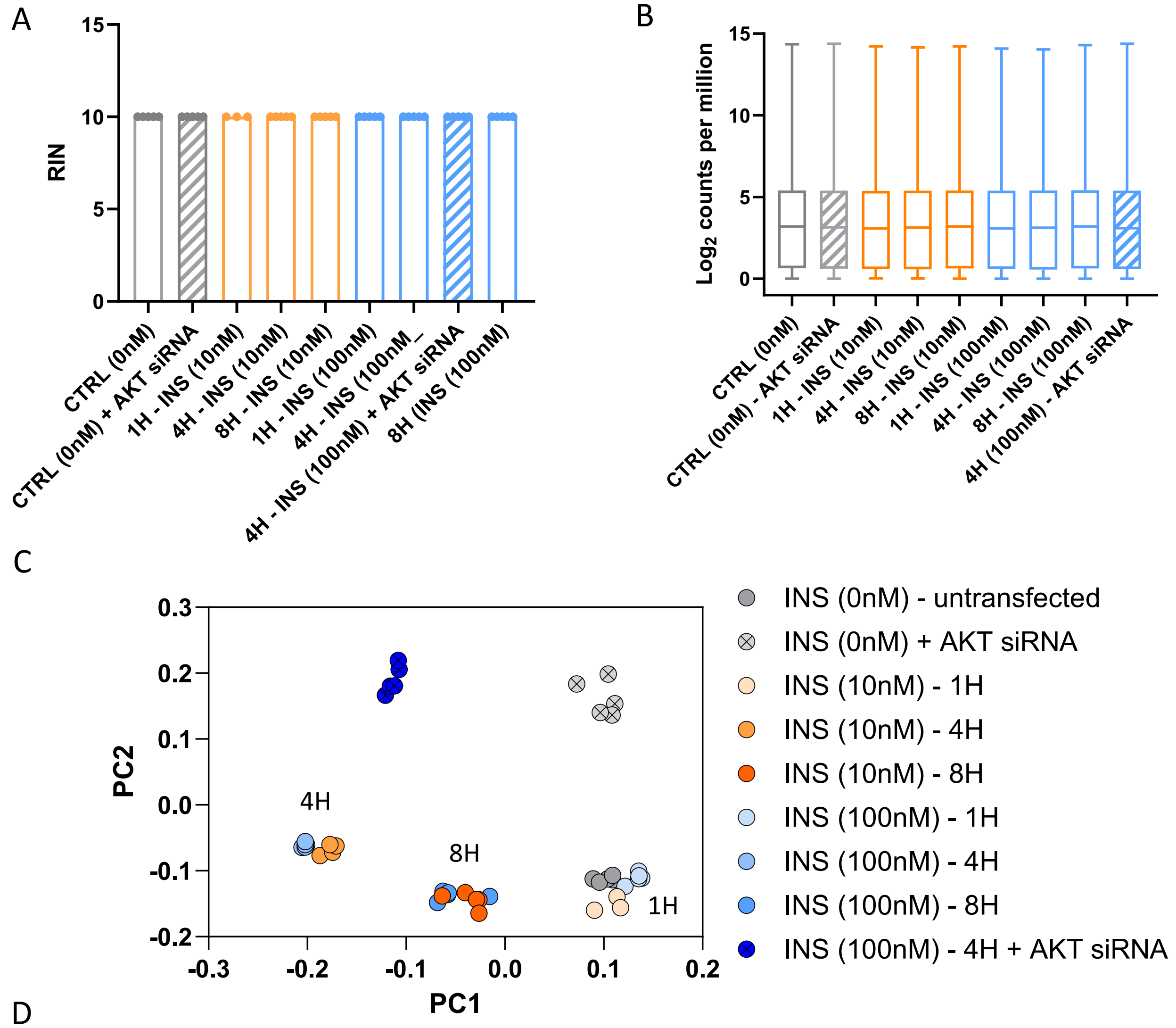

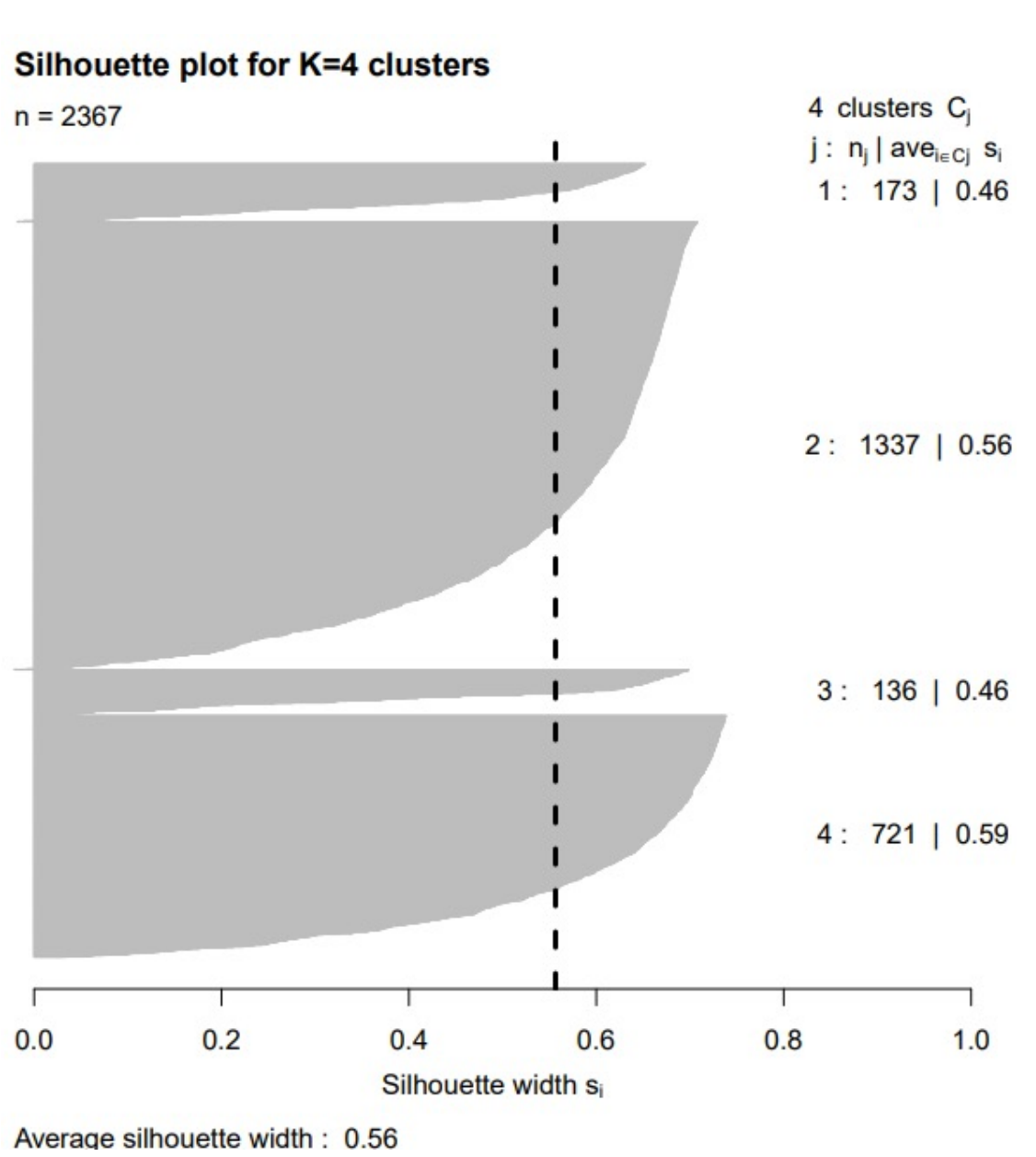

Supplementary Figure 10. (A) RNA integrity number (RIN) analysis of control and insulin-treated cells (n=3-5 replicates per condition). Data shown as mean ±SEM. **(B)** Sample counts obtained from MCF-12A transcriptomes. Raw counts were normalised for sequencing depth and expressed as log2 counts per million showing uniform distribution between samples. Data shown as box plots. **(C)** Principal components analysis of untransfected and AKT siRNA-transfected cells with or without insulin. Biological replicates (n=5) from each condition cluster together with clear separation of control and insulin groups. **(D)** Silhouette analysis showing grouping of genes into K=4 clusters based on temporal changes in expression. Average silhouette width (aveiÎCj s_i) indicates degree or quality of clustering. j =cluster, i = gene, n_j = number of genes assigned to a cluster.

Supplementary Figure 11. Number of overlapping differentially expressed genes in MCF-12A cells treated with 10 nM and 100 nM insulin

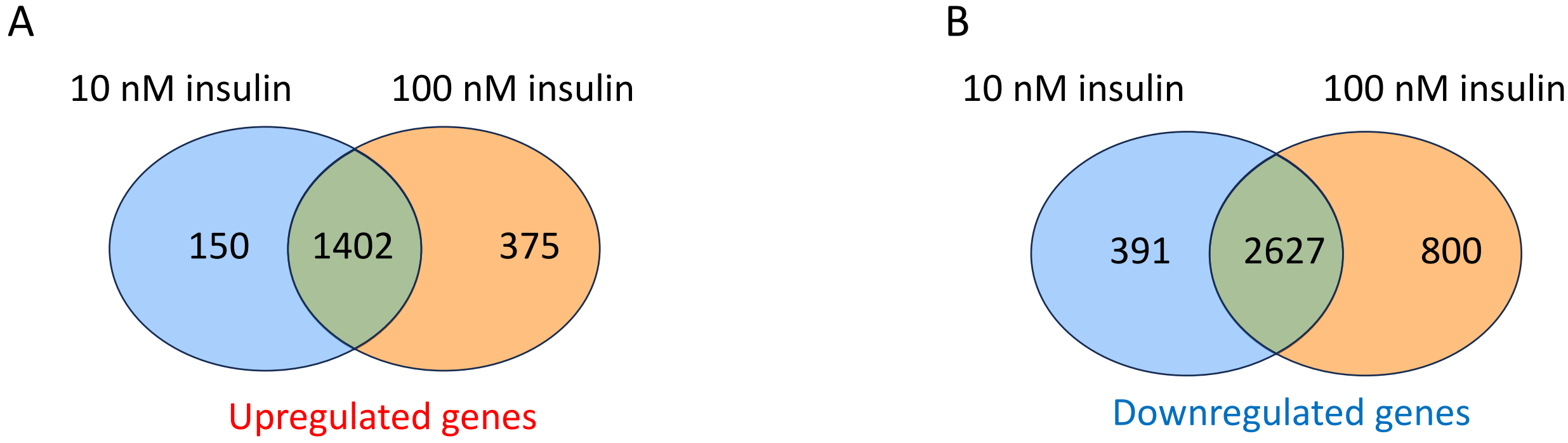

Supplementary Figure 11. Venn diagrams showing the number of overlapping **(A)** upregulated and **(B)** downregulated genes between MCF-12A cells treated with from 10 nM or 100 nM insulin.

Supplementary Figure 12. Characterization of beta-casein, insulin receptor and insulin-mediated Akt signalling in HC11 cells

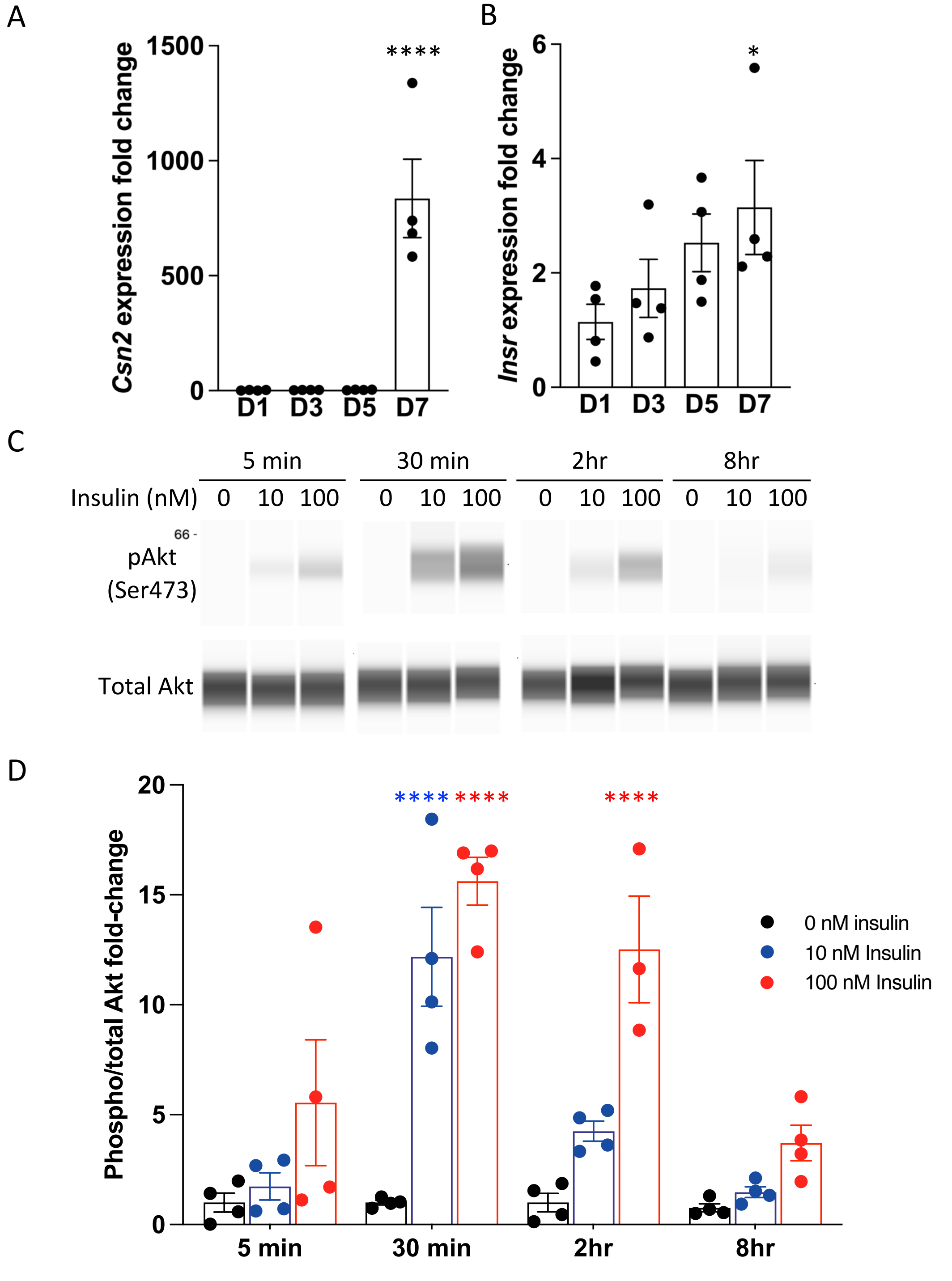

Supplementary Figure 12. (**A**) *Csn2* and (**B**) *Insr* expression in HC11 cells were longitudinally assessed during cell differentiation over 7 days. Fold-changes are shown in comparison to day 1 expression values. D day. *p<0.05 and ****p<0.0001 compared to cells at day 1. (**C**) Virtual capillary protein electrophoresis blots showing phospho- and total-Akt responses at 5 min, 30 min, 2 hours and 8 hours in cells treated with 0, 10 or 100 nM insulin. (**E**) Quantitative analysis of pAkt expression normalised to total Akt expression (n=4 biological replicates). Data shown as mean±SEM. ****p<0.0001 compared to untreated HC11 cells.

Supplementary Figure 13. RNA quality assessment, principal components analysis, and clustering of HC11 transcriptomic data

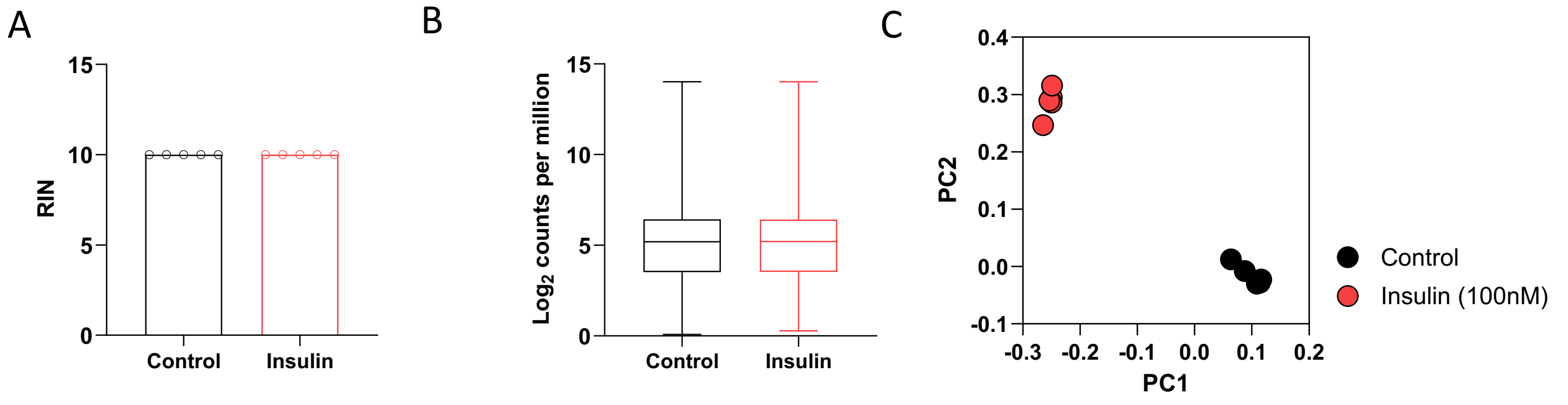

Supplementary Figure 13. (A) RNA integrity number (RIN) analysis of control and insulin-treated cells (n=5 replicates per condition). Data shown as mean ± SEM. (**B)** Sample counts obtained from HC11 transcriptomes. Raw counts were normalized for sequencing depth and expressed as log_2 counts per million showing uniform distribution between samples. Data shown as box plots. (**C)** Principal components analysis of control cells and cells treated with insulin for 8 hours. Biological replicates (n=5) from each condition cluster together with clear separation of control (black) and insulin (red) groups.

Supplementary Figure 14. Mitochondrial processes influenced by insulin in HC11 cells

A

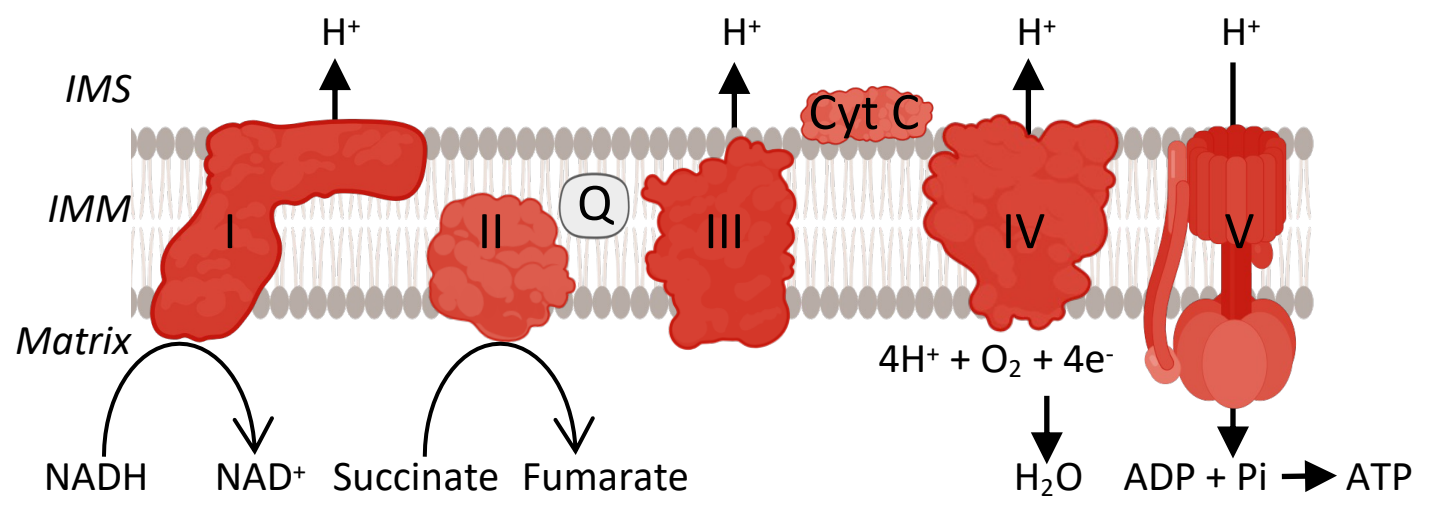

B

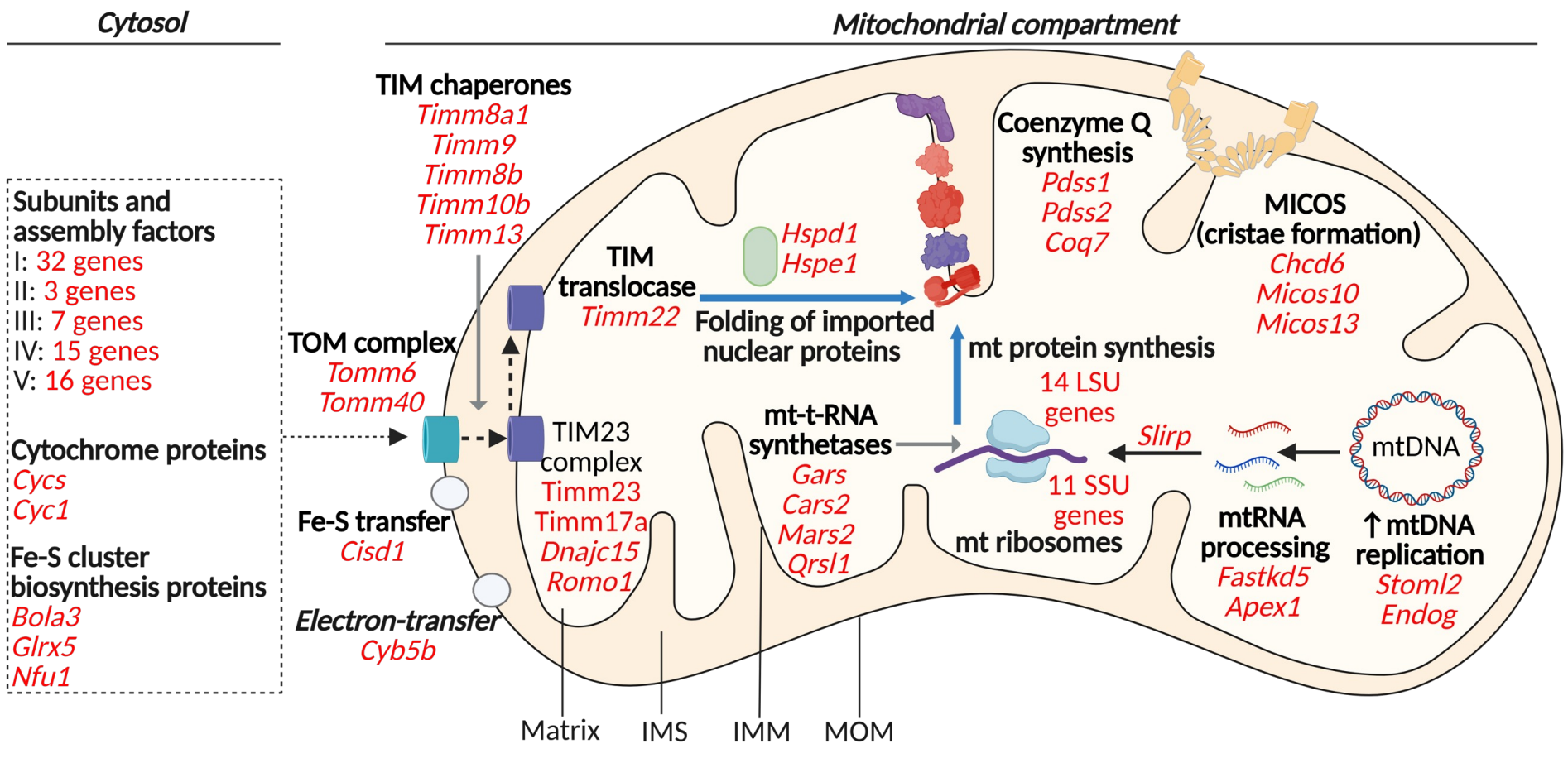

Supplementary Figure 14. (A) Schematic of electron transport chain showing complexes encoded by upregulated genes in HC11 cells (red). Q, co-enzyme Q; Cyt C, cytochrome C. **(B)** Schematic showing involvement of upregulated genes (red) in mitochondrial biogenesis processes in HC11 cells. IMS, inter-mitochondrial membrane space; IMM, inner mitochondrial membrane; MOM, mitochondrial outer membrane.

Supplementary Figure 15. Transmission electron microscopy of mitochondria in HC11 cells

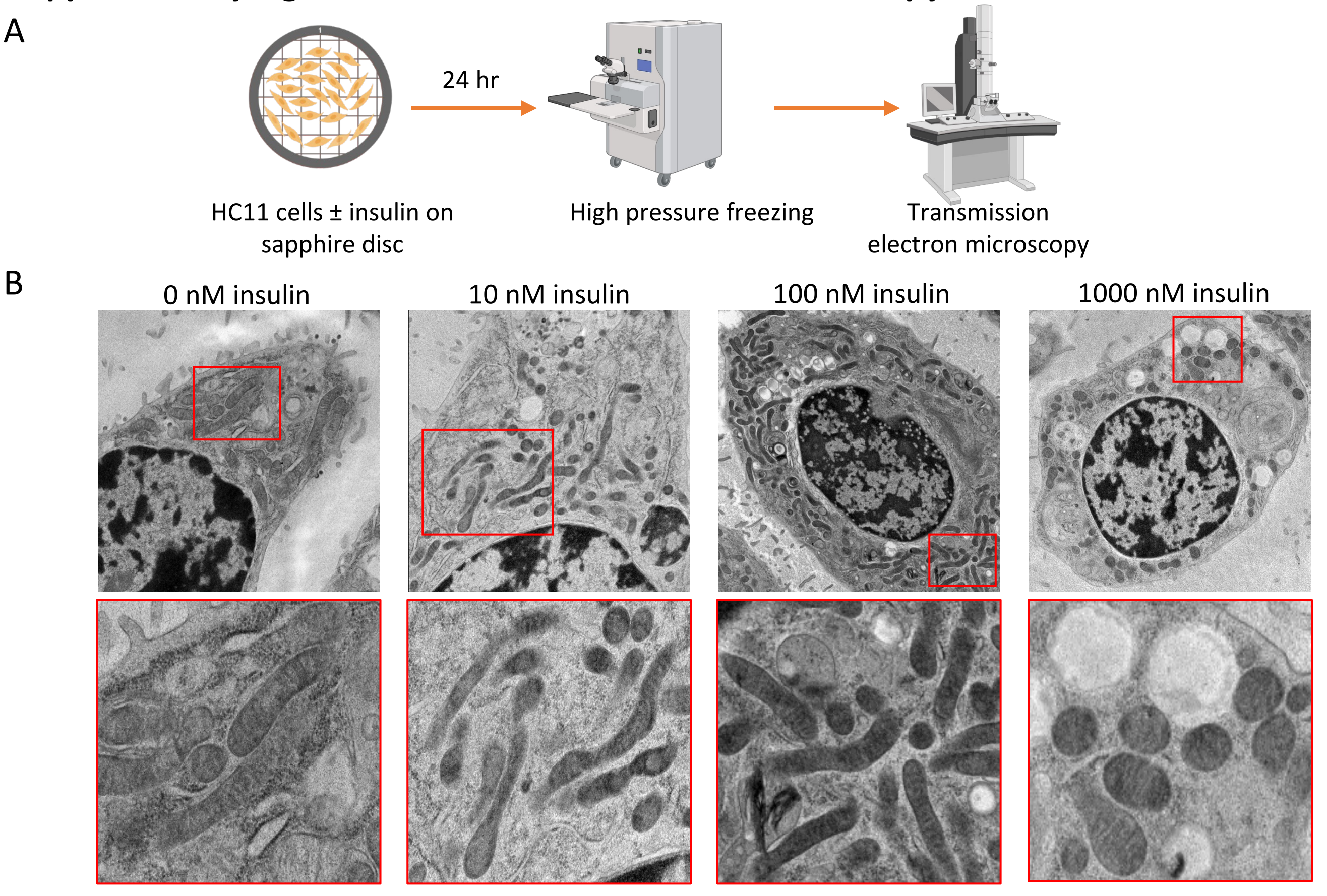

Supplementary Figure 15. (A) Protocol for transmission electron microscopy involving high pressure freezing of cells treated with insulin for 24 hours.**(B)** Representative mitochondrial images are shown for each condition, with zoomed-in images shown at the bottom.

Supplementary Figure 16. PGC-1α expression in response to insulin treatment and evaluation of transcription factors involved in insulin-mediated mitochondrial biogenesis

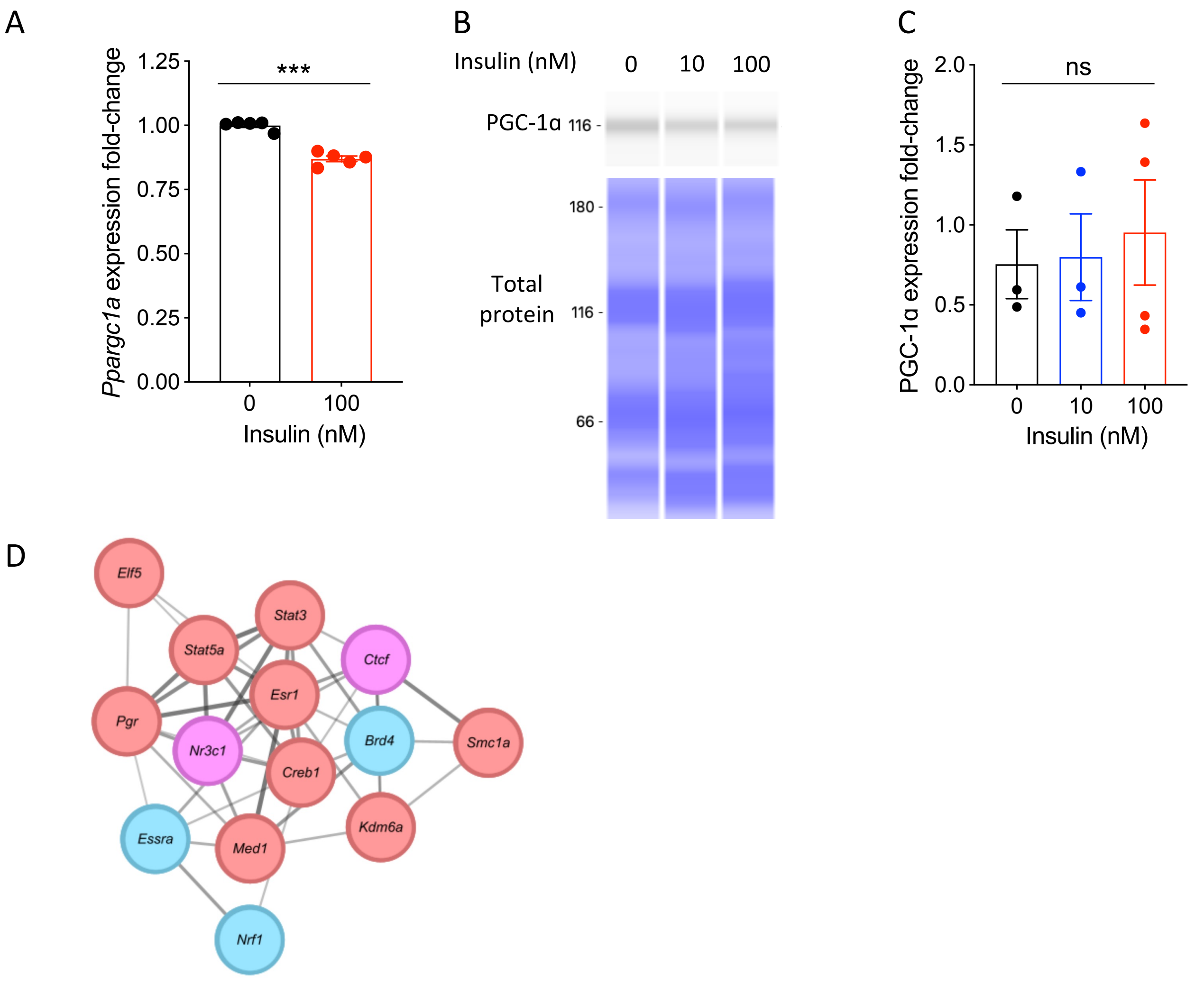

Supplementary Figure 16. (A) PGC-1α RNA expression in HC11 cells treated with 0 or 100 nM insulin for 8 hours. Data shown as mean±SEM. ***p<0.0001 (unpaired t-test). **(B)** Capillary protein electrophoresis blots showing PGC-1α protein expression and total protein in HC11 cells treated with 0, 10 or 100 nM insulin for 8 hours. **(C)** Quantitative analysis of PGC-1α protein expression normalised to total protein (n=3-4 biological replicates). Data shown as mean±SEM. **(D)** STRING network analysis of transcription factors predicted to interact with differentially expressed mitochondrial genes identified in insulin-treated cells. Nodes represent genes and edge thickness indicate interaction confidence level. Orange and blues nodes represent transcription factors identified in either lactating mammary gland or skeletal muscle, respectively. Pink nodes represent transcription factors identified in both lactating mammary gland and skeletal muscle.

Supplementary Figure 17. Examples of protein blot and qPCR technical replicates

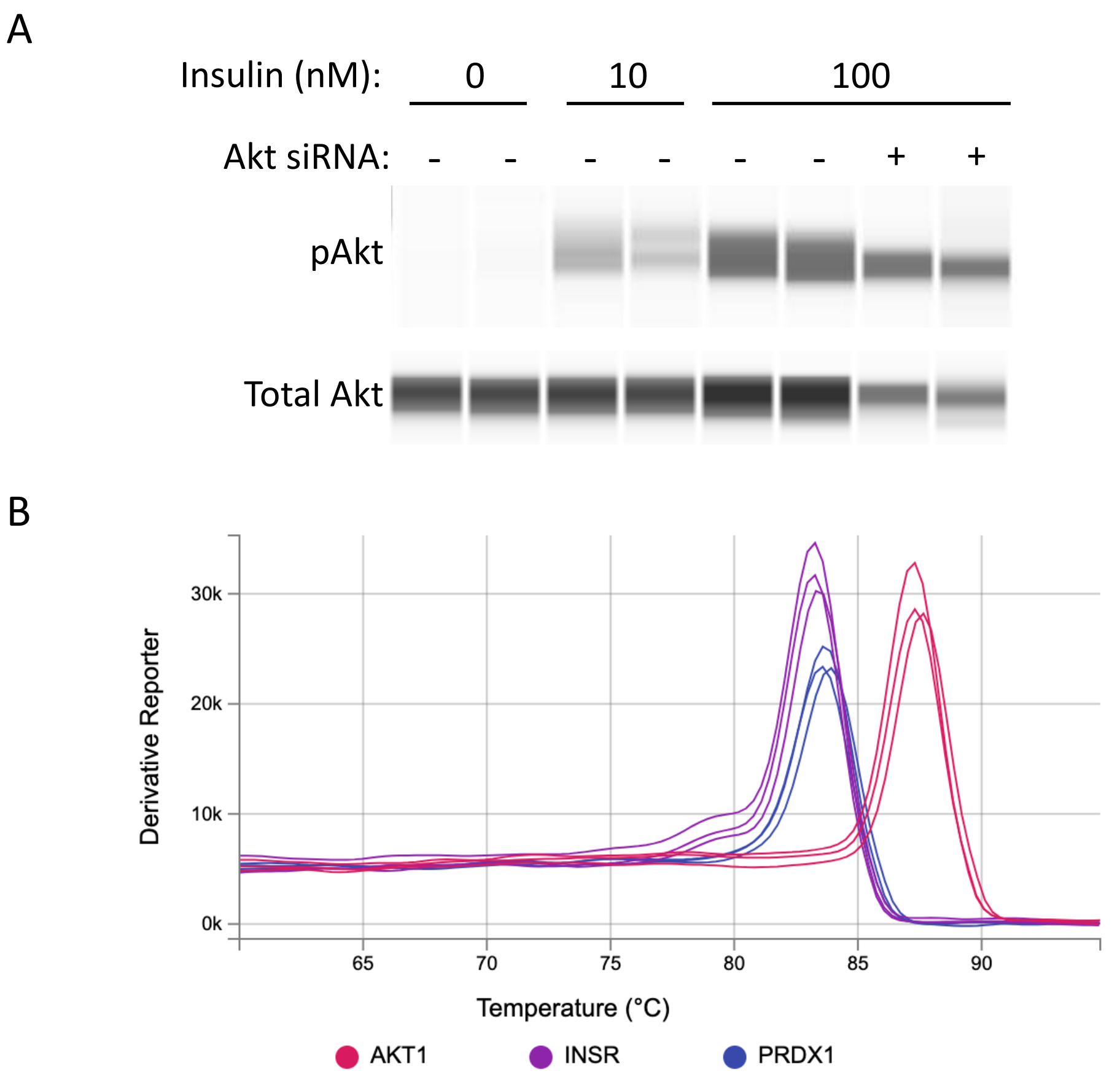

Supplementary Figure 17. (A) Representative protein blots showing duplicate analyses of phospho-Akt (pAkt) and total Akt proteins (n=2 technical replicates). **(B)** Representative qPCR melt curve plots showing expression analysis of target genes (*AKT1, INSR* and *PRDX1*) performed in triplicate (n=3 technical replicates).

Supplemental Methods

Study site

This single centre study was undertaken at the John Radcliffe Hospital, Oxford, UK; which is a tertiary referral center with a maternity unit and infant feeding team. Although not Baby Friendly Initiative (BFI) accredited, all mothers receive guidance on breastfeeding during antenatal visits. Early postpartum care includes mothers rooming-in with their infants, and encouragement to initiate skin-to-skin contact as soon as possible after birth and commence breastfeeding within the first postpartum hour.

Participant exclusion criteria

Participants were excluded if there was a history of prior breast surgery, presence of severe maternal illness including postpartum depression or psychosis, infant prematurity or severe infant illness including major congenital abnormalities, or if there was prolonged separation of infant from mother such as admission to the neonatal unit (1). No participants had a prior diagnosis of breast hypoplasia.

Flow cytometry analysis of the milk fat globule fraction

Flow cytometry was performed with a Cytek® Northern Lights three-laser flow cytometer (Cytek Biosciences, USA), and spectral profiles of each fluorophore were unmixed using SpectroFlo® software (Cytek Biosciences, USA), and appropriate single-stain and unstained controls used to account for autofluorescence. Instrument startup and quality control was performed prior to acquisition according to the manufacturer's instructions. Volumetric counting was undertaken on sample volumes of 200 µL to obtain absolute quantification of $CD45^+$ and $CD45^-$ cells in the MFG fraction. Manual gating was applied to identify singlet nucleated structures, as reported (2, 3). The number of cells in the MFG fraction obtained from 1mL of milk was calculated.

Induction of β-casein expression in cultured mammary cells

Differentiation of the human MCF-12A cell line to β-casein expressing cells was performed by culturing cells on Transwell inserts with 0.4 µm pore size (Sarstedt, Germany) coated with growth-factor-reduced Matrigel (1:4 dilution, CORNING, USA), as reported (4). The differentiation protocol involved cells being cultured in complete medium for one day, followed by 3 days in differentiation media consisting of DMEM/F12 with 5% horse serum, 1.4 µM hydrocortisone, 0.1 µg/mL human prolactin (Thermo Fisher Scientific, USA), and 10 nM insulin (Sigma, USA).

Differentiation of the murine HC11 cell line to β-casein expressing cells was performed by growing cells in complete medium until confluency, followed by 2 days of EGF withdrawal and then culturing for 2 days in in differentiation media consisting of RPMI 1640 medium with 0.1 µM dexamethasone (Sigma, USA), 1 µg/mL prolactin (Thermo Fisher Scientific, USA), and 10 nM insulin (Sigma, USA) (5).

siRNA transfection

MCF-12A cells were seeded onto Matrigel-coated transwell inserts at a density of $3x10^5$ cells/insert in a 6-well plate. Upon reaching 70-80% confluency in complete medium without penicillin/streptomycin, cells were transfected with either Silencer® siRNA targeting Akt (Gene ID: 103719) or a non-targeting Silencer™ select siRNA (scramble) (ThermoFisher Scientific, USA). Transfection was performed using Lipofectamine RNAiMAX (ThermoFisher Scientific, USA) in penicillin/streptomycin-free differentiation medium. In brief, siRNA-lipid complexes were prepared in Opti-MEM® I reduced serum medium (Thermo Fisher Scientific, USA), and following 20 min incubation, were added to cells to achieve an siRNA concentration of 50 nM. Akt RNA and protein expression were assessed at 48 hours and 72-96 hours, respectively, following siRNA transfection.

RNA sequencing

RNA samples were quantified using Qubit 4.0 Fluorometer (Life Technologies, USA) and RNA integrity assessed using the Agilent 5300 Fragment Analyzer (Agilent Technologies, USA). For MFG samples, ribosomal RNA depletion was used for sequencing library preparation. For intact mammary

cell line RNA (RIN>6), poly-A selection with Oligo (dT) beads was used. RNA libraries were prepared using the NEBNext Ultra II RNA Library Prep Kit for Illumina (NEB, USA). Sequencing was undertaken using the Illumina NovaSeq 6000 with 2x150 paired-end reads and a sequencing depth of 50 million reads per sample. Raw sequence data was converted into fastq files and de-multiplexed using Illumina bcl2fastq program version 2.20. Sequences were aligned to the mouse (mm9, NCBI Build 37) or human (hg19, GRCh37) genomes to generate read count tables.

Differential gene expression analysis

Sequencing data was analysed using Omics Playground (BigOmics Analytics, Switzerland). Pre-processing of raw counts included normalization to sequencing depth and log transformation to yield $\log_2$ counts per million (CPM). Filtering was applied so that transcripts with CPM≥1 in at least k samples were kept, where k = size of smallest experimental group. To account for differences in RNA integrity in the MFG dataset, we used Omics Playground built-in surrogate variable analysis (SVA) method using RIN values as covariates. To increase statistical reliability, DEG analysis was performed using limma, edgeR, and DESeq2 for cultured cell data; whereas DESeq2 (Wald and likelihood ratio tests) was used to assess MFG DEGs. FDR-adjusted p-values (q) were combined where meta-q for a gene represents the lowest q-value among the methods. Significant DEGs were based on $\log_2$ fold change values >0.5 for upregulated genes and <-0.5 for downregulated genes, with meta-q threshold of 0.05. For exploratory analysis of MFG data, the meta-q threshold was lowered to 0.1.

Biological pathway enrichment and gene-set enrichment analysis (GSEA) was undertaken using GO, WikiPathways, Reactome, BioPlanet, InterPro, HumanCyc datasets, with statistical analysis involving Fisher's exact test, single-sample GSEA (ssGSEA), and gene set variation analysis (GSVA). Statistical significance was determined based on mean $\log_2$ fold change within each gene set with threshold values >0.5 for upregulation and <-0.5 for downregulation, and using q- or meta-q<0.05.

Comparison of MFG transcriptome with other tissues

The Enrichr web-based tool was to compare the MFG transcriptome with other tissues (6). The most highly expressed MFG genes (top 10%) on days 2-5 postpartum were assessed for enrichment using human GTEX and Mouse Gene Atlas (7-9). Enrichment scores were calculated based on the adjusted p-value (Fisher's exact test with Benjamini-Hochberg correction for multiple hypotheses) and odds ratio calculated as: odds ratio = (1.0*a*d)/Max(1.0*b*c, 1), where: *a* represents the number of genes shared between the input and annotated gene sets; *b* and *c* are the numbers of genes unique to the annotated set and the input set, respectively; and *d* corresponds to the number of genes in the background that are present in neither the input nor the annotated set. Enrichment was considered significant at adjusted $p<0.05$.

Analysis of temporal gene expression using TMixClust

TMixClust utilised transcript per million (TPM) values with expression values for each gene normalized to mean of 0 and standard deviation of 1. The mean expression value for control and treatment groups were calculated at each study time-point, followed by subtraction of the control group mean value from the treatment group mean value. This procedure resulted in a 2,367 x 4 matrix (rows = genes, columns = time points) for input into TMixClust. Analysis of this dataset using a range of K=2 to 8 clusters was performed, with 10 clustering runs and the iteration with the highest likelihood selected per value of K. Silhouette plots generated by TMixClust were used to select the value of K with the best clustering cohesion, with K=4 providing the best solution and larger values of K overfitting the data. For cluster GSEA, filtering was applied after clustering to only include genes that were also expressed in human MFG transcriptomes (with median TPM >0.5). This resulted in 1,086 genes from the MCF-12A dataset being used for cluster GSEA.

In silico transcription factor analysis

Transcription factor identification involved significance thresholds being set to an FDR-adjusted p value of 10^{-50} calculated from Model-based Analysis of ChIP-Seq (MACS) computational method (10). We used the STRING database (https://string-db.org/) to generate an interaction network for transcription factors binding to mitochondrial genes. The minimum required interaction score was set

to 0.4 and singletons excluded. The network diagram was then modified using Cytoscape (3.10.3) (https://cytoscape.org/).

In silico inflammatory gene analysis

HumanBase was used to generate a mammary gland-specific interaction network of inflammatory genes (11). Briefly, inflammatory genes were determined by cross-referencing MFG DEGs with the inflammatory response gene set (Hallmark dataset) (12). Genes with shared functions based on GO terms were clustered together in functional modules (13). Nodes and edges represent genes and functional interactions between genes, respectively. Node size corresponds to the number of direct connections to other nodes within the network.

Quantitative PCR analysis of cultured mammary cells

MCF-12A gene expression was assessed by qPCR using predesigned human primers (Sigma, USA): *CSN2* (Forward: 5′-GCTCAACCAAGAACTTCTAC, Reverse: 5′-CACTAATGGGGTTATGAACTG). *INSR* (Forward: 5′- GATCCAATCTCAGTGTCTAAC, Reverse: 5′-CCTTTGAGGCAATAATCCAG), *AKT1* (Forward: 5′-AAGTACTCTTTCCAGACCC, Reverse: 5′- TTCTCCAGCTTGAGGTC), *AKT2* (Forward: 5′- CACCATGAATGAGGTGAATAC, Reverse: 5′-CTACGGAGAAGTTGTTTAAGG), *AKT3* (Forward: 5′- TTCTTCTCTGGAGTAAACTGG, Reverse: 5′- TTGCTGACATTTTTCAGGTG). Housekeeping genes included *FAM190B* ((Forward: 5′- GGAGTGTGACAATATGAACC, Reverse: 5′- TCTGCTCAAGTCACTTTTTC), *YWHA2* (Forward: 5′- AACTTGACATTGTGGACATC, Reverse: 5′- AAAACTATTTGTGGGACAGC), *SYMPK* (Forward: 5′-ATGAGGACAAAGACTTGGAG, Reverse: 5′-GACCAGATTAGCCACATTATC).

HC11 gene expression was assessed by qPCR using predesigned mouse primers (Sigma, USA): *Csn2* (Forward: 5′-GTATTTCCAGTGAGGAATCTG, Reverse: 5′-ATTGCAAGAGATGGTTTGAG) and *Insr* (Forward: 5′-AAGACCTTGGTTACCTTCTC, Reverse: 5′-GGATTAGTGGCATCTGTTTG). Housekeeping genes included *Actb* (Forward: 5′-

GATGTATGAAGGCTTTGGTC, Reverse: 5′-TGTGCACTTTTATTGGTCTC), *B2m* (Forward: 5′-CGAACATACTGAACTGCTAC, Reverse: 5′-AGCCAGGATATAGAAAGACC), and *Prdx1* (Forward: 5′-CATGGATTACCATAGTTGGC, Reverse: 5′-CCTAGTCTACAGAGTTCCAG). Stock primers (100 µM) were diluted to 10 µM in RNase-free water and stored at -20°C until further use. Each qPCR reaction was prepared with PowerUP SYBR Green PCR master mix (Applied Biosystems, USA) in a final volume of 10 µL and conducted on the QuantStudio 3 real-time PCR system (Applied Biosystems, USA). Samples were analysed in triplicate. Threshold cycle (Ct) values were determined using Design and Analysis software (Thermo Fisher Scientific, USA). Mean Ct values for each sample were normalized to the housekeeping gene geometric mean and expressed as fold-change relative to control using the $2^{\wedge-\Delta\Delta Ct}$ method.

Capillary-based protein electrophoresis of cultured mammary cells

Phospho- and total protein quantification was performed using capillary-based electrophoresis (JESS, Simple Western™ instrument, Bio-Techne, USA). Protein lysates were diluted in 0.1x sample buffer and 1x fluorescent master mix to achieve a final concentration of 0.3-0.6 µg/µL in the presence of molecular weight markers and 400 mM dithiothreitol (SimpleWestern, Bio-Techne, USA). This preparation was denatured at 95°C for 5 min and a total of 4 µL was loaded into capillaries in the 12–230 kDa JESS separation module (SimpleWestern, Bio-Techne, USA) along with antibodies and chemiluminescence reagents, described as follows: primary antibodies were purchased from Cell Signalling Technology (USA) and prepared in antibody diluent (SimpleWestern, Bio-Techne, USA), including: phospho-Akt (S473) (D9E #4060, 1:100 dilution), total Akt (E7J2C #58295, 1:100 dilution), and PGC-1ɑ (3G6 #2178, 1:50 dilution); chemiluminescence detection was conducted using peroxide/luminol-S and anti-rabbit (#042-206) or anti-mouse (#042-205) HRP-secondary antibodies (#DM-001 and #DM-002, SimpleWestern, Bio-Techne, USA). Chemiluminescence intensity plotted against the migration distance corresponding to protein molecular weight were used to generate electropherograms using the Compass for SimpleWestern analysis software. The protein detection module (#DM-TP01, SimpleWestern, Bio-Techne, USA) was used to quantify lysate total protein.

Target proteins were quantified based on area-under-the-curve of the target peak and visualized as a virtual blot. Target protein expression was normalized to total protein or total Akt.

Confocal mitochondrial imaging

Images were acquired using an Olympus IXplore Spin-SR, using a 50 µm pinhole confocal spinning disk and a Hamamatsu ORCA Fusion BT camera. Imaging was performed using a 60x silicone oil lens with a numerical aperture of 1.3. Z-stacks were acquired spanning a total depth of 11.34 µm with z-spacing of 0.42 µm. Cells were imaged in a 37°C and 5% CO_2 environmental chamber. This study involved n=2 biological replicates (2 individual wells) per treatment. Around 7,000-10,000 mitochondria were imaged from 5-10 images per treatment group obtained from n=2 individual wells.

Mitochondrial morphology and volume analysis

Analysis was performed using the ImageJ plug-in Mitochondria Analyzer. Images were converted into 8-bit and transformed into binary images using adaptive thresholding (block size: 1.45 µm, C-value: 13). For 2D analysis, maximum intensity projections were generated from z-stacks, thresholded, and analysed using the 2D pipeline (14). Measurements of mitochondrial length and number of branches were obtained from each image. For 3D analysis, z-stacks comprised of 27 slices were thresholded and analysed using the 3D pipeline (14) to assess mitochondrial volume, and the sphericity index, which indicates how closely each mitochondrion resembles a perfect sphere calculated as $36\pi(V^2/S^3)$, where V is volume and S is surface area.

Transmission electron microscopy mitochondrial imaging

HC11 cells were seeded onto poly-l-lysine-coated sapphire discs in 24-well plates and underwent differentiation to β-casein expressing cells over 7 days. Insulin was removed overnight followed by insulin treatment (0, 10, 100 and 1000 nM) for 24 hours. High-pressure freezing (EM PACT2, Leica, Germany) was used to preserve native cell structures. Samples were maintained at −90°C in an AFS2 (Leica, Germany) freeze substitution device for 44 hours in a solution of 0.1% uranyl acetate, 1% osmium tetroxide (TAAB Laboratory Equipment, UK), and 0.5% anhydrous glutaraldehyde (Agar

Scientific, UK). The temperature was then raised to 4°C over a period of 25 hours through a series of controlled warming phases. Samples were washed with acetone (Sigma, USA), infiltrated with low viscosity resin (TAAB Laboratory Equipment, USA), and polymerized at 60°C. Sections of 90 nm were collected on EM grids and imaged using a 120KV transmission electron microscope (JEOL 1400Flash TEM). This study involved n=2 biological replicates (2 individual wells) per treatment.

Oxygen consumption rate and extracellular acidication rate assays

Cell respiration was assessed using MitoXpress Intra Intracellular oxygen assay and pH-Xtra Glycolysis Assay, respectively (Agilent Technologies, USA). HC11 cells were seeded in 96-well plates (10^4 cells per well), underwent differentiation to β-casein expressing cells, and incubated overnight in insulin-free RPMI 1640 medium containing 0.1 µM dexamethasone and 1 µg/ml prolactin prior to OCR and ECAR measurements. For OCR measurement, cells were treated for 24 hours with 0, 10, or 100 nM insulin prepared in insulin-free medium containing 0.1 µM dexamethasone, 1 µg/ml prolactin, and 5% MitoXpress Intra (Agilent Technologies, USA). Where indicated, 0.1 uM FCCP (Sigma, USA) was used to measure maximal OCR following insulin treatment (15). For ECAR measurement, cells received the same insulin treatment, followed by CO_2-depletion for 2 hours. Cells were then washed with respiration buffer (1mM K-phosphate, 20 mM Glucose, 70 mM NaCl, 50 mM KCl, 0.8 mM $MgSO_4$, 2.4 mM $CaCl_2$) and 10% pH-Xtra (Agilent Technologies, USA) was added. Where indicated, 25 mM 2-deoxyglucose (Sigma, USA) and 1 µM oligomycin (Sigma, USA) were added to measure glycolytic capacity. Measurements were performed on the CLARIOstar plate reader (BMG Labtech, Germany) at 37°C and recorded every 15 min for 4 hours at ~21% O_2. Fluorescence intensities of MitoXpress and pH-Xtra, were measured in dual-read time-resolved fluorescence (TR-F) mode (Agilent Technologies, USA). OCR was calculated as the rate of decline in media O_2 during the period where O_2 concentration showed a linear decline. ECAR was calculated as the change in pH-Xtra probe lifetime per min (µs/min), during the period where lifetime showed a linear change. This study involved n=5-8 biological replicates (5-8 individual wells) per treatment.

Supplemental References

1. Rostom H, Meng X, Price H, Fry A, Elajnaf T, Humphrey R, et al. Protocol for an observational study investigating hormones triggering the onset of sustained lactation: the INSIGHT study. *BMJ Open.* 2022;12(8):e062478.
2. Schultz-Pernice I, Engelbrecht LK, Petricca S, Scheel CH, Twigger A-J, Schultz-Pernice I, et al. Morphological Analysis of Human Milk Membrane Enclosed Structures Reveals Diverse Cells and Cell-like Milk Fat Globules. *Journal of Mammary Gland Biology and Neoplasia 2020 25:4.* 2021-01-04;25(4).
3. Twigger A-J, Engelbrecht LK, Bach K, Schultz-Pernice I, Pensa S, Stenning J, et al. Transcriptional changes in the mammary gland during lactation revealed by single cell sequencing of cells from human milk. *Nature Communications 2022 13:1.* 2022-01-28;13(1).
4. Chiba T, Kimura S, Takahashi K, Morimoto Y, Sanbe A, Ueda H, et al. Serotonin suppresses beta-casein expression via inhibition of the signal transducer and activator of transcription 5 (STAT5) protein phosphorylation in human mammary epithelial cells MCF-12A. *Biol Pharm Bull.* 2014;37(8):1336-40.
5. Berlato C, and Doppler W. Selective response to insulin versus insulin-like growth factor-I and -II and up-regulation of insulin receptor splice variant B in the differentiated mouse mammary epithelium. *Endocrinology.* 2009;150(6):2924-33.
6. Chen EY, Tan CM, Kou Y, Duan Q, Wang Z, Meirelles GV, et al. Enrichr: interactive and collaborative HTML5 gene list enrichment analysis tool. *BMC Bioinformatics.* 2013;14:128.
7. Consortium GT. The GTEx Consortium atlas of genetic regulatory effects across human tissues. *Science.* 2020;369(6509):1318-30.
8. Su AI, Wiltshire T, Batalov S, Lapp H, Ching KA, Block D, et al. A gene atlas of the mouse and human protein-encoding transcriptomes. *Proc Natl Acad Sci U S A.* 2004;101(16):6062-7.
9. Wei J, Ramanathan P, Martin IC, Moran C, Taylor RM, and Williamson P. Identification of gene sets and pathways associated with lactation performance in mice. *Physiol Genomics.* 2013;45(5):171-81.
10. Feng J, Liu T, Qin B, Zhang Y, and Liu XS. Identifying ChIP-seq enrichment using MACS. *Nature protocols.* 2012 Aug 30;7(9).
11. Greene CS, Krishnan A, Wong AK, Ricciotti E, Zelaya RA, Himmelstein DS, et al. Understanding multicellular function and disease with human tissue-specific networks. *Nature Genetics 2015 47:6.* 2015-04-27;47(6).
12. Liberzon A, Birger C, Thorvaldsdóttir H, Ghandi M, Mesirov Jill P, and Tamayo P. The Molecular Signatures Database Hallmark Gene Set Collection. *Cell Systems.* 2015/12/23;1(6).
13. Gene Ontology C, Aleksander SA, Balhoff J, Carbon S, Cherry JM, Drabkin HJ, et al. The Gene Ontology knowledgebase in 2023. *Genetics.* 2023;224(1).
14. Chaudhry A, Shi R, and Luciani DS. A pipeline for multidimensional confocal analysis of mitochondrial morphology, function, and dynamics in pancreatic β-cells. *American Journal of Physiology-Endocrinology and Metabolism.* 2020 Jan 21.
15. Samartsev VN, Semenova AA, and Dubinin MV. A comparative study of the action of protonophore uncouplers and decoupling agents as inducers of free respiration in mitochondria in states 3 and 4: theoretical and experimental approaches. *Cell Biochemistry and Biophysics.* 2020;78:203-16.

Conflict of Interest: Patent Information

Pending patent applications:

U.S. application no.	Publication no.	Title	Co-inventors / notes
**18/830,190**	**US 2025/0000885 A1**	**Composition Comprising Two or More Oligosaccharides**	Michael I. Goran, Bradley Scott Peterson, Lars Bode, Ravi Bansal; Children's Hospital Los Angeles. Google Patents currently lists it as **Pending**. (Google Patents)
**18/111,103**	**US 2023/0201228 A1**	**Immunomodulatory Oligosaccharides**	Lars Bode, Philip Gordts; UC San Diego. **Pending**. (Google Patents)
**18/107,936**	**US 2023/0181609 A1**	**Immunomodulatory Oligosaccharides**	Lars Bode, Philip Gordts; UC San Diego. **Pending**. (Google Patents)
**18/100,204**	**US 2023/0149433 A1**	**Immunomodulatory Oligosaccharides**	Lars Bode, Philip Gordts; UC San Diego. **Pending**. This application is related to the two applications immediately above. (Google Patents)
**17/584,129**	**US 2022/0143056 A1**	**Methods and Therapies for Treating Atherosclerosis and/or Hyperlipidemia Using Oligosaccharides**	Philip Gordts, Ariane Pessentheiner, Lars Bode; UC San Diego. **Pending**. (Google Patents)

Issued Patent Applications:

U.S. patent no.	Title	Inventors
**US 11,896,603 B2**	**Oligosaccharide as Therapeutic Agent for Alcohol Associated Liver Disease**	Lars Bode, Bernd Schnabl. Issued Feb. 13, 2024. (Justia Patents)
**US 11,234,992 B2**	**Methods and Therapies for Treating Atherosclerosis and/or Hyperlipidemia Using Oligosaccharides**	Philip Gordts, Ariane Pessentheiner, Lars Bode. Issued Feb. 1, 2022. (Google Patents)
**US 10,160,986 B2**	**Chemoenzymatic Synthesis of Heparin and Heparan Sulfate Analogs**	Xi Chen, Hai Yu, Yanhong Li, Yi Chen, Jingyao Qu, Musleh M. Muthana, Kam Lau, Lars Bode. Issued Dec. 25, 2018. (Google Patents)

Conflict of Interest: Patent Information

**US 9,675,649 B2**	**Disialyllacto-N-Tetraose (DSLNT) or Variants, Isomers, Analogs and Derivatives Thereof to Prevent or Inhibit Bowel Disease**	Lars Bode. Issued June 13, 2017. (Google Patents)
**US 9,290,530 B2**	**Chemoenzymatic Synthesis of Heparin and Heparan Sulfate Analogs**	Xi Chen, Hai Yu, Kam Lau, Lars Bode et al. Issued March 22, 2016. This is in the same general patent family as US 10,160,986. (Google Patents)

For international patents/applications (in addition to US), see the list below:

**Patent family / title**	**International / foreign numbers I could verify**	**Status / notes**
**Immunomodulatory Oligosaccharides**	**PCT/US2018/054433**; **WO2019071021A2 / A3**	PCT filed Oct. 4, 2018; Lars Bode & Philip Gordts. The international PCT phase has ceased because the application proceeded into national phases. (Google Patents)
	**CA3076920A1**	Canadian application; currently shown as **pending**. (Google Patents)
	**MX2020004133A**	Mexican national-stage application. (Google Patents)
**Methods and Therapies for Treating Atherosclerosis and/or Hyperlipidemia Using Oligosaccharides**	International/national family corresponding to priority beginning **Feb. 28, 2018**	Inventors Philip Gordts, Ariane Pessentheiner & Lars Bode; UC San Diego. The U.S. family produced US11,234,992 and the pending continuation US20220143056. (Google Patents)

**Disialyllacto-N-tetraose (DSLNT) or Variants, Isomers, Analogs and Derivatives Thereof to Prevent or Inhibit Bowel Disease**	**WO2012106665A2 / A3**	Major international HMO/NEC patent family; PCT filed Feb. 3, 2012. Lars Bode is the inventor. (Google Patents)
	**EP2670399A2 / A4**; application **EP12742191.5**	European national/regional phase. (Google Patents)
	**CA2824960A1**	Canada
	**CN201280007735.6 / CN103442696B**	China; Chinese patent granted.
	**NZ613432 / NZ613432B2**	New Zealand; granted patent
	**MX2013008500A**	Mexico
	**SG2013058185A / SG192234A1**	Singapore
	**PH1/2013/501481 / PH12013501481A1**	Philippines (Google Patents)
	**BR112013019522A / A2**	Brazil
	**IL227398A**	Israel. These national-stage filings are identified in the WO family record. (Google Patents)
**New Agents to Treat/Prevent Amoebiasis**	**PCT/US2012/023863**; **WO2012106662A2 / A3**	Lars Bode, Tineke Lauwaet, Evelyn Jantscher-Krenn & Frances Gillin; UC Berkeley/UC San Diego. PCT filed Feb. 3, 2012; international phase ceased. (Google Patents)
**Chemoenzymatic Synthesis of Heparin and Heparan Sulfate Analogs**	**PCT/US2012/047875**; **WO2013013244A2**	International family underlying the U.S. patents. Priority July 21, 2011; Lars Bode is one of multiple inventors. (Google Patents)
