# Supplementary material for: Insulin resistance is associated with mammary mitochondrial dysfunction at the onset of human lactation
Source: J Clin Invest. 2026 Aug 13;136(20):e197311. doi: 10.1172/JCI197311 (PMC13668628; doi:10.1172/JCI197311)

Full unedited blot for **Figure 4 A (upper panel).** Blots shown in Figure 4a are highlighted in the red squares

Antibody: Phospho-Akt (Ser473) (D9E) #4060 (Cell Signaling Technology)

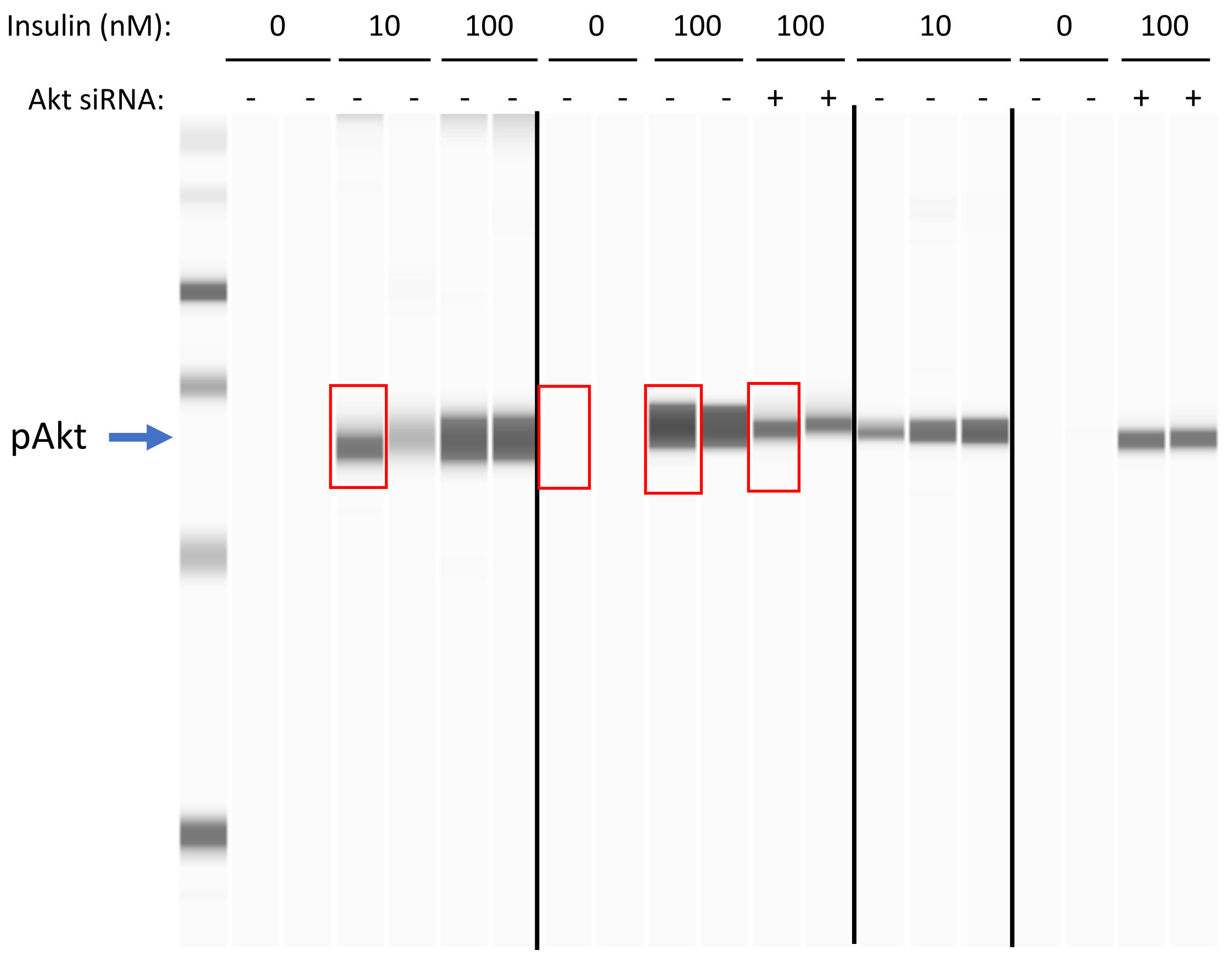

Full unedited blot for **Figure 4A (lower panel).** Blots shown in Figure 4a are highlighted in the red squares

Antibody: Total Akt (E7J2C) #58295 (Cell Signaling Technology)

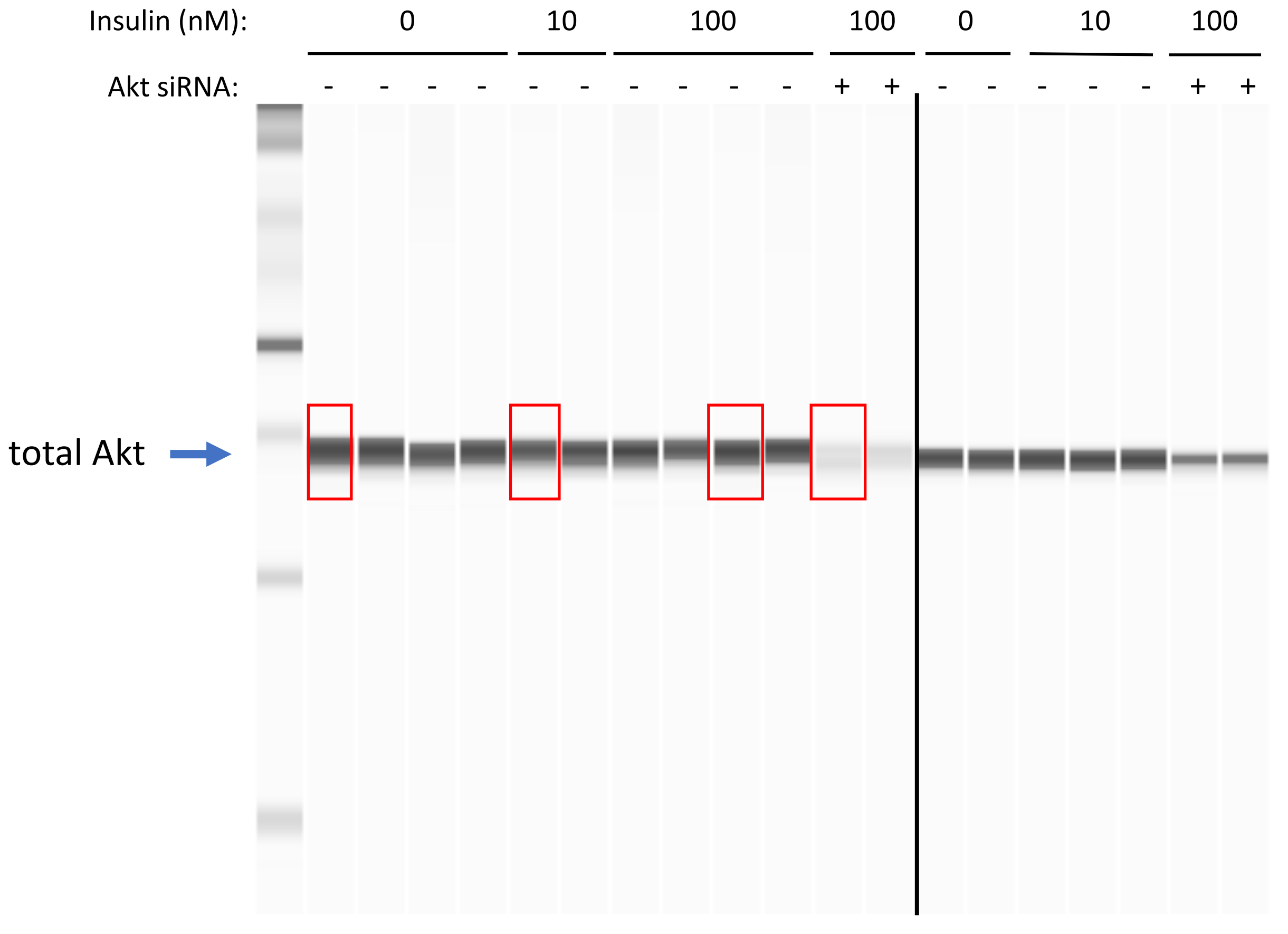

Full unedited blot for **Supplementary Figure 12C (upper panel).** Blots shown in Figure S12C are highlighted in the red squares

Antibody: Phospho-Akt (Ser473) (D9E) #4060 (Cell Signaling Technology)

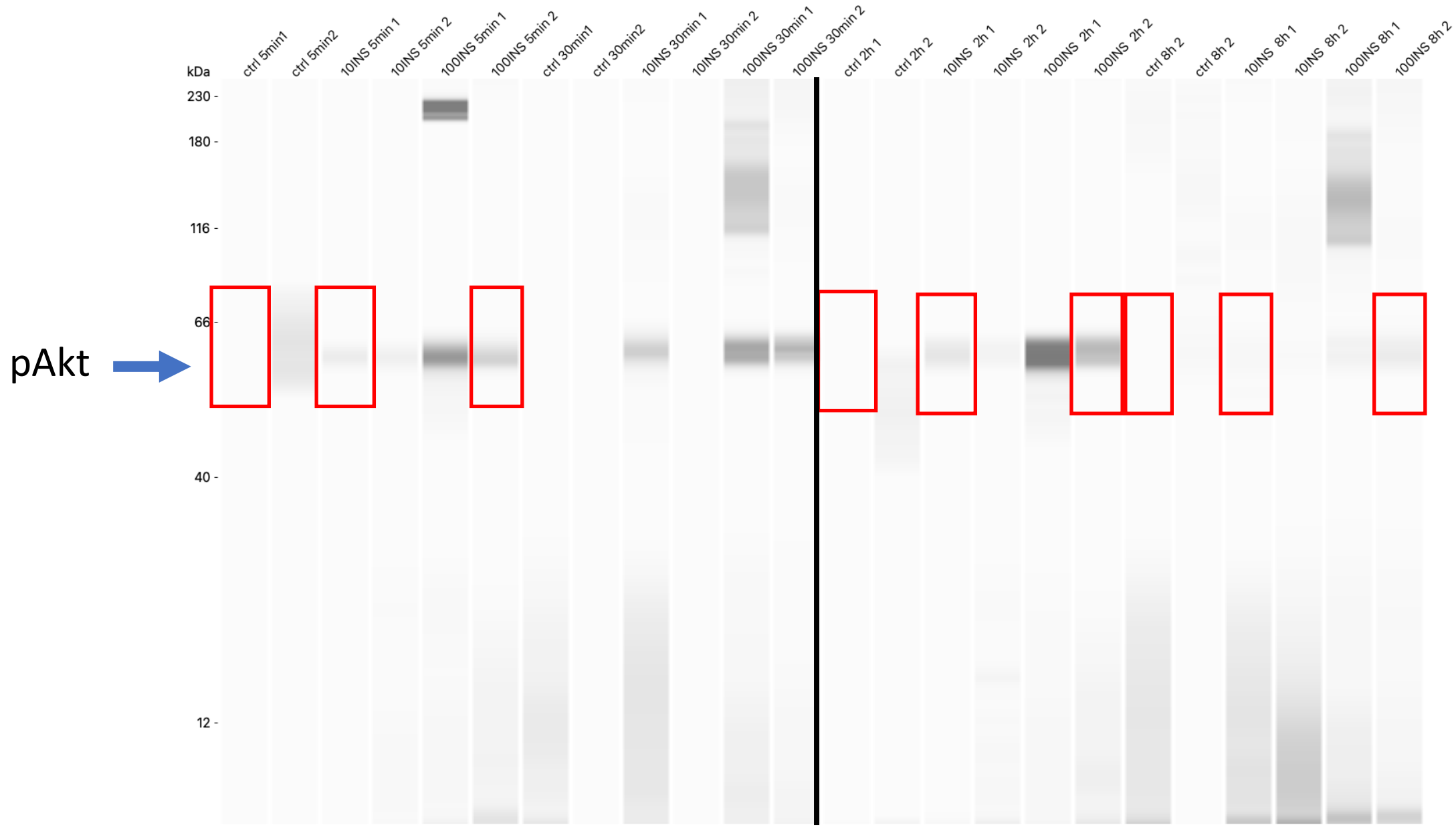

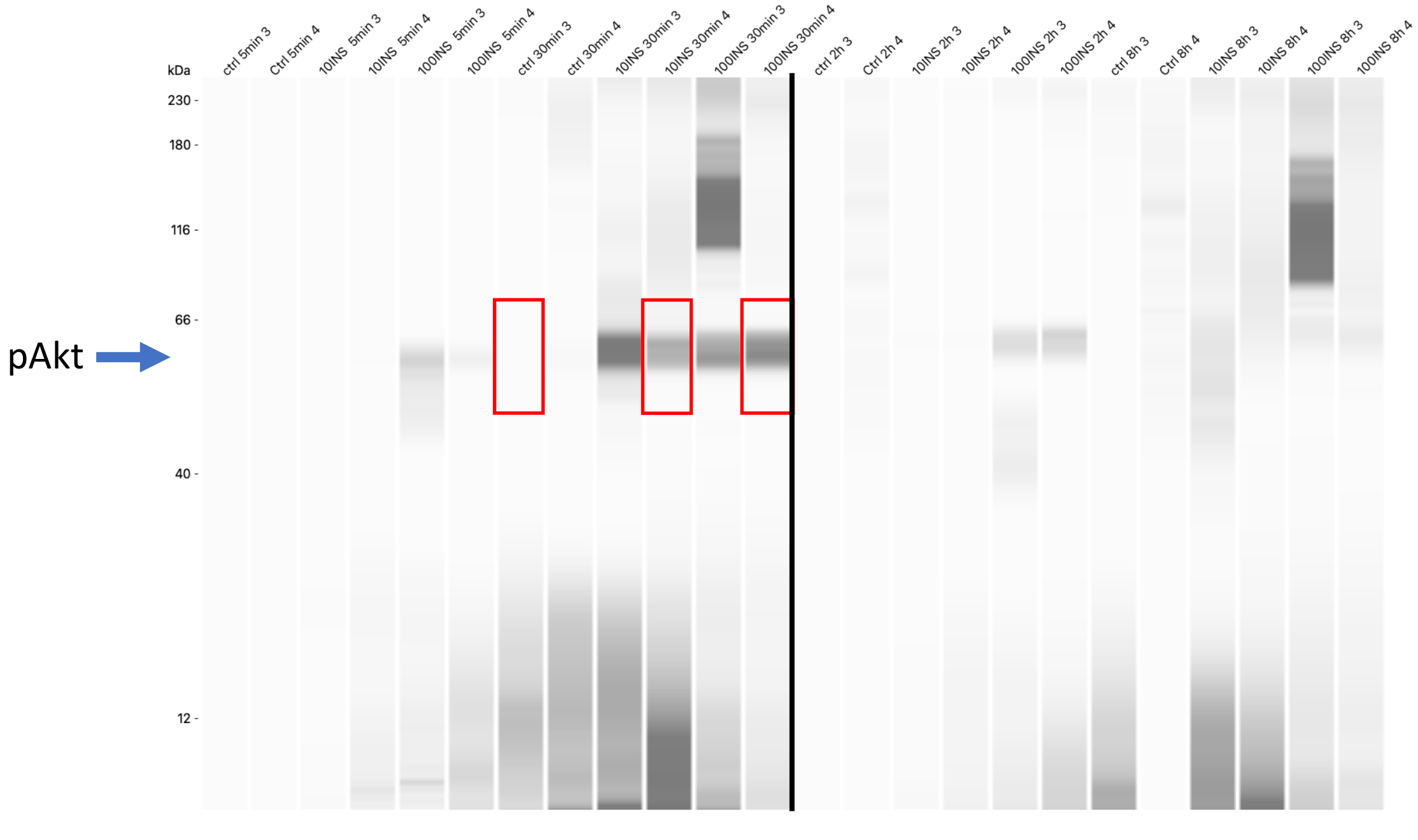

Full unedited blot for **Supplementary Figure 12C (lower panel).** Blots shown in Figure S12C are highlighted in the red squares

Antibody: Total Akt (E7J2C) #58295 (Cell Signaling Technology)

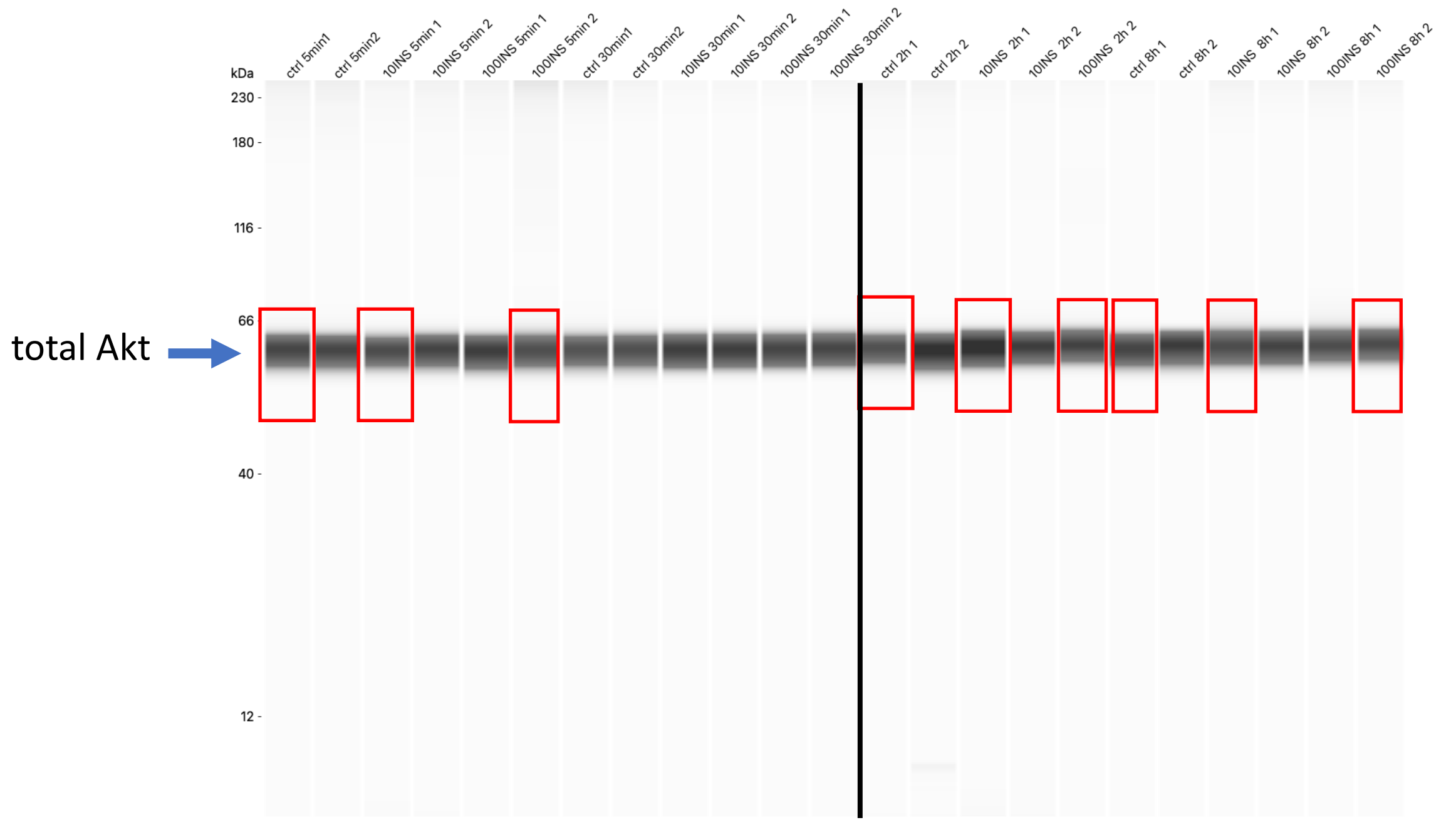

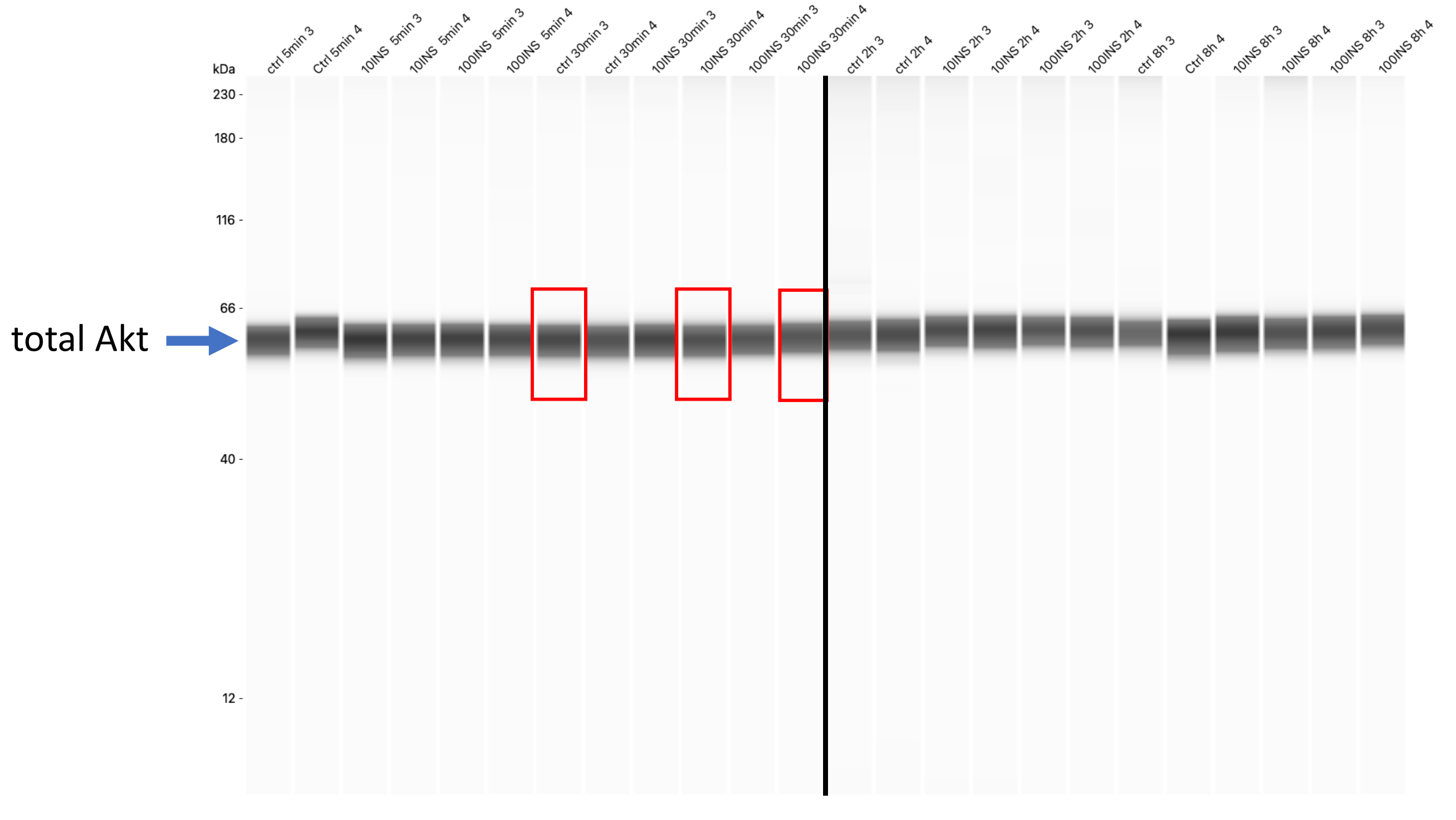

Full unedited blot for **Supplementary Figure 16B (upper panel).** Blots shown in Figure S16B are highlighted in the red squares

Antibody: PGC-1 alpha (3G6) #2178 (Cell Signaling Technology)

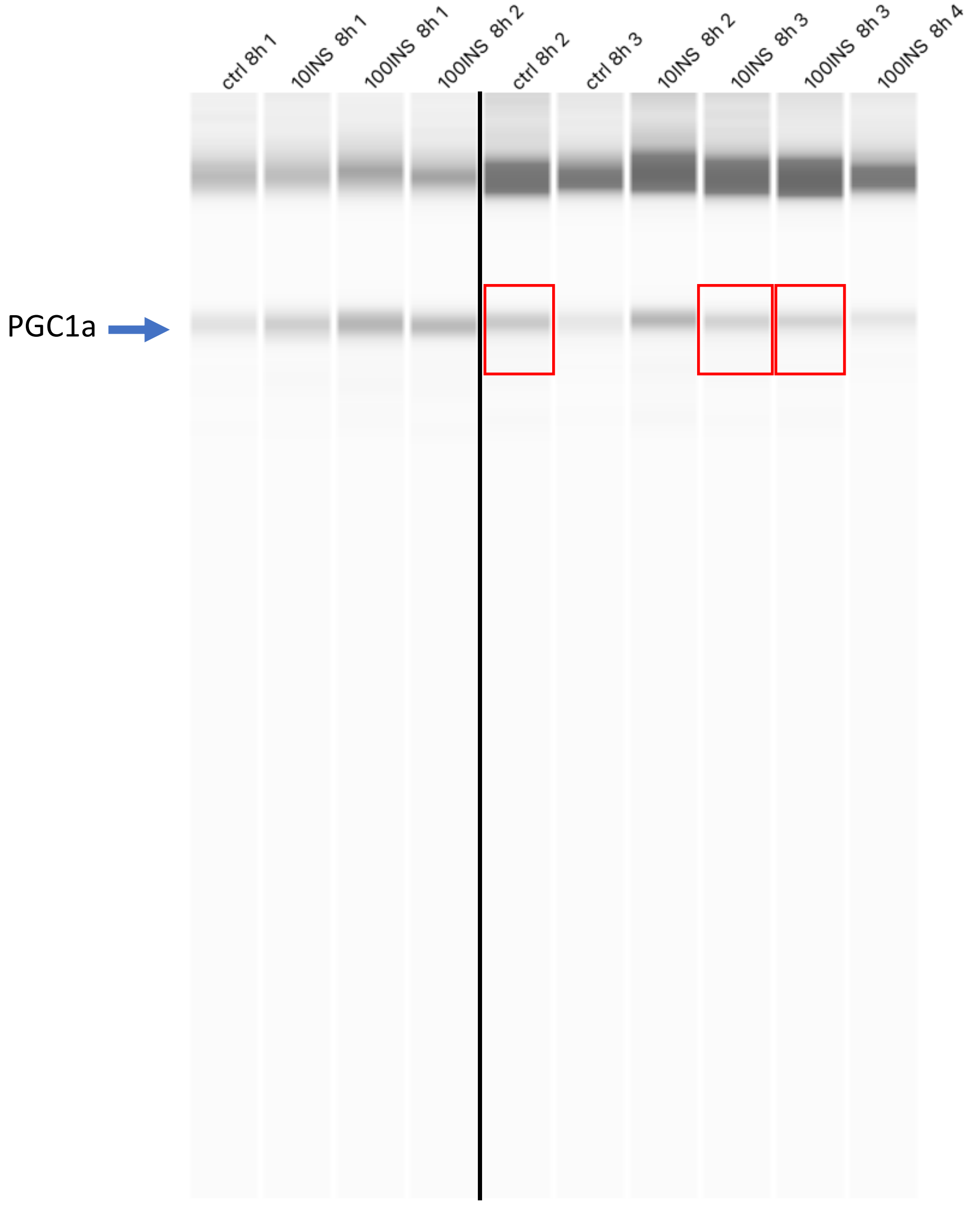

Full unedited blot for **Supplementary Figure 16B (lower panel).**
Blots shown in Figure S16B are highlighted in the red squares

Total protein blots

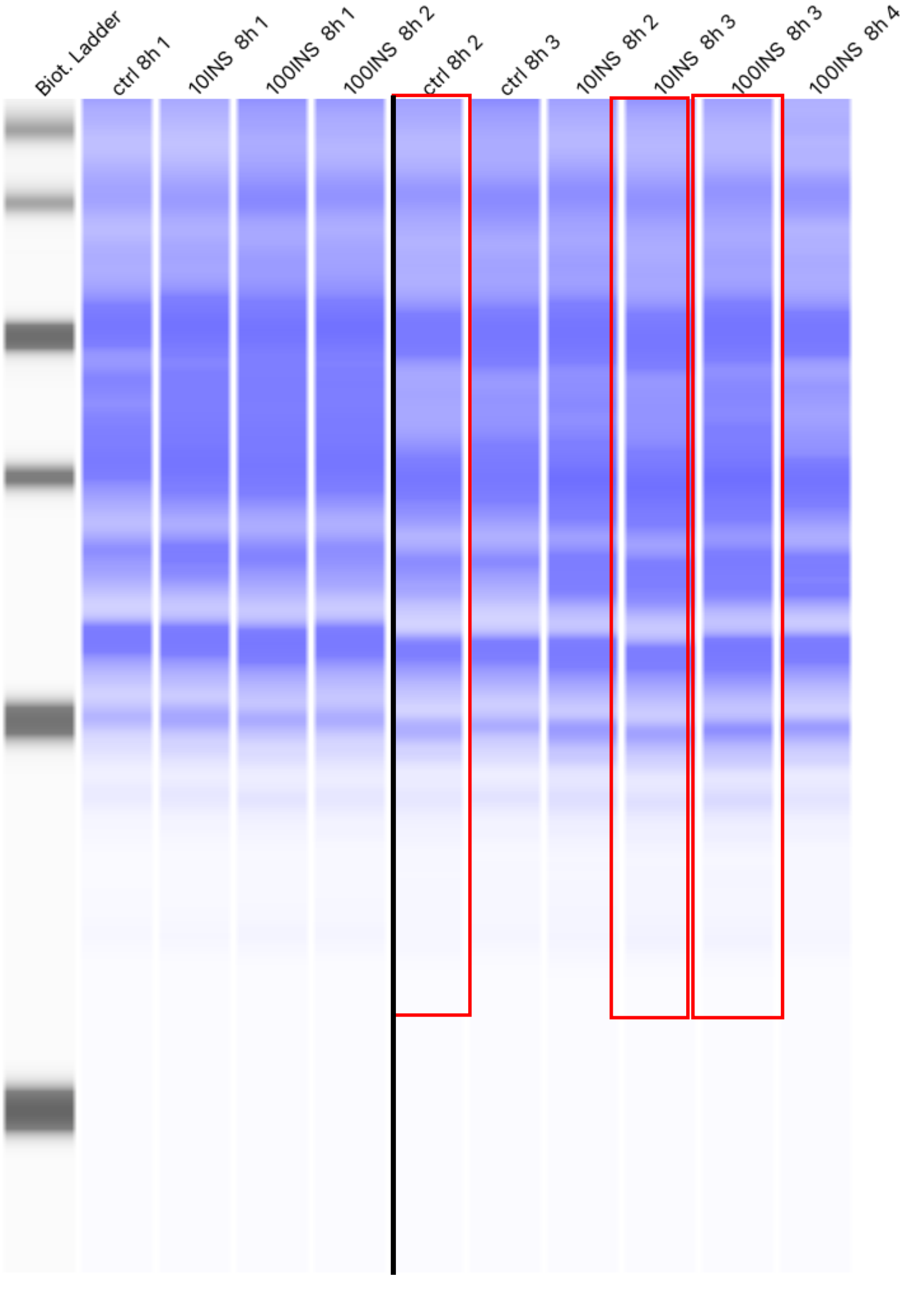

Supplement: Supplementary file 2 [file jci-136-197311-s264.pdf]
